# Arachidonic acid activates NLRP3 inflammasome in MDSCs via FATP2 to promote post-transplant tumour recurrence in steatotic liver grafts

**DOI:** 10.1016/j.jhepr.2023.100895

**Published:** 2023-08-22

**Authors:** Hui Liu, Wai Ho Oscar Yeung, Li Pang, Jiang Liu, Xiao Bing Liu, Kevin Tak Pan Ng, Qingmei Zhang, Wen Qi Qiu, Yueqin Zhu, Tao Ding, Zhe Wang, Ji Ye Zhu, Chung Mau Lo, Kwan Man

**Affiliations:** 1 Department of Surgery, School of Clinical Medicine, HKU-SZH and LKS Faculty of Medicine, The University of Hong Kong, Hong Kong, China; 2 Department of Pathophysiology, Key Laboratory of Cell Differentiation and Apoptosis of the Chinese Ministry of Education, Shanghai Jiao Tong University School of Medicine, Shanghai, China

**Keywords:** Steatotic liver graft, Tumour recurrence, MDSC, Inflammasome, Lipid metabolism

## Abstract

**Background & Aims:**

The steatotic grafts have been applied in liver transplantation frequently owing to the high incidence of non-alcoholic fatty liver disease. However, fatty livers are vulnerable to graft injury. Myeloid-derived suppressor cell (MDSC) recruitment during liver graft injury promotes tumour recurrence. Lipid metabolism exerts the immunological influence on MDSCs in tumour progression. Here, we aimed to explore the role and mechanism of inflammasome activation in MDSCs induced by lipid metabolism during fatty liver graft injury and the subsequent effects on tumour recurrence.

**Methods:**

MDSC populations and nucleotide-binding oligomerisation domain-like receptor family pyrin domain containing 3 (NLRP3) inflammasome levels were investigated in a clinical cohort and a rat liver transplantation model. The mechanism of NLRP3 activation by specific fatty acids was explored in mouse hepatic ischaemia/reperfusion injury (IRI) with tumour recurrence model and *in vitro* studies.

**Results:**

MDSC populations and NLRP3 levels were increased with higher tumour recurrent rate in patients using steatotic grafts. NLRP3 was upregulated in MDSCs with lipid accumulation post mouse fatty liver IRI. Mechanistically, arachidonic acid was discovered to activate NLRP3 inflammasome in MDSCs through fatty acid transport protein 2 (FATP2), which was identified by screening lipid uptake receptors. The mitochondrial dysfunction with enhanced reactive oxygen species bridged arachidonic acid uptake and NLRP3 activation in MDSCs, which subsequently stimulated CD4^+^ T cells producing more IL-17 in fatty liver IRI. Blockade of FATP2 inhibited NLRP3 activation in MDSCs, IL-17 production in CD4^+^ T cells, and the tumour recurrence post fatty liver IRI.

**Conclusions:**

During fatty liver graft injury, arachidonic acid activated NLRP3 inflammasome in MDSCs through FATP2, which subsequently stimulated CD4^+^ T cells producing IL-17 to promote tumour recurrence post transplantation.

**Impact and implications:**

The high incidence of non-alcoholic fatty liver disease resulted in the frequent application of steatotic donors in liver transplantation. Our data showed that the patients who underwent liver transplantation using fatty grafts experienced higher tumour recurrence. We found that arachidonic acid activated NLRP3 inflammasome in MDSCs through FATP2 during fatty liver graft injury, which led to more IL-17 secretion of CD4^+^ T cells and promoted tumour recurrence post transplantation. The inflammasome activation by aberrant fatty acid metabolism in MDSCs bridged the acute-phase fatty liver graft injury and liver tumour recurrence.

## Highlights

• NLRP3 and MDSCs were increased in fatty liver grafts associated with a higher rate of tumour recurrence.
• Arachidonic acid activated NLRP3 inflammasome in MDSCs through FATP2.
• NLRP3 and MDSCs were increased in fatty liver grafts associated with a higher rate of tumour recurrence.
• Targeting FATP2 inhibited tumour recurrence post liver transplantation.

## Graphical abstract

**Figure:**
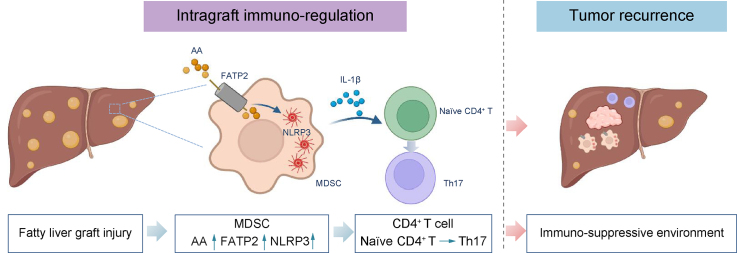

## Introduction

The drastic donor shortage is still a huge problem for liver transplantation. The high incidence of non-alcoholic fatty liver disease resulted in the frequent application of steatotic donor livers.[1], [2], [3] However, fatty donor livers are vulnerable to graft injury.^4^^,^^5^ For transplant recipients with hepatocellular carcinoma (HCC), graft injury may reshape the graft immune microenvironment through recruiting several immunosuppressive cells, such as regulatory T cells and myeloid-derived suppressor cells (MDSCs), which subsequently promote tumour recurrence.[5], [6], [7], [8] Our previous study found the tendency of poor recurrence-free survival in patients who received graft with >10% steatosis after living-donor liver transplantation.^4^ Nevertheless, the responses of immune cells to steatotic liver graft injury and their roles and mechanisms for liver tumour recurrence have not been well illustrated.

MDSCs represent a heterogeneous population of myeloid progenitor cells that not only disrupt the surveillance of tumours by crosstalking with other immune cells[9], [10], [11], [12] but also directly promote cancer stemness, angiogenesis, and metastasis.^13^ Recent studies reported that the immune function of MDSCs could be modulated by lipid metabolism. The uptake and oxidation of fatty acid increases in tumour-infiltrating MDSCs, and the inhibition of fatty acid oxidation enhances the cancer therapies.^14^ Moreover, the tumour-derived factors facilitate lipid uptake, oxidative metabolism, and tolerogenic functional reprogramming of MDSCs.^15^ In patients with cancer, the specific marker of granulocytic MDSCs (G-MDSCs) is associated with endoplasmic reticulum stress and lipid metabolism.^16^ G-MDSCs acquired immunosuppressive activity mediated by fatty acid transport protein 2 (FATP2).^17^ Our previous findings indicated that MDSCs were recruited into liver graft during acute-phase injury, promoting tumour recurrence post transplantation.^18^ However, the roles and mechanisms of lipid metabolism in MDSCs in steatotic liver graft injury have never been explored.

As pattern recognition receptors to sense the danger signals, inflammasomes process the release of bioactive IL-1β and IL-18 via caspase-1 activation and induce cell pyroptosis.^19^ Inflammasome activation plays critical roles in liver diseases.^20^ In non-alcoholic steatohepatitis, saturate fatty acids activate the inflammasomes in hepatocytes, and the danger signals released by hepatocytes could further trigger the inflammasome activation in immune cells.^21^ Blockade of nucleotide-binding oligomerisation domain-like receptor family pyrin domain containing 3 (NLRP3) inflammasome reduces the liver inflammation and fibrosis in a mouse non-alcoholic steatohepatitis model.^22^ We reported that NLRP3 inflammasome activation in neutrophils induced liver graft injury via the telomere-independent repressor activator protein 1 (RAP1)/keratinocyte chemoattractant (KC) axis post transplantation.^23^ Gene silencing of NLRP3 inflammasome may protect against liver ischaemia/reperfusion injury (IRI).^24^ Decreased NLRP3 inflammasome expression has been found in HCC tissues compared with non-cancerous tissues although its role in tumorigenesis can be opposing.^25^ In MDSCs, NLRP3 inflammasome is activated via cathepsin B release triggered by chemotherapy and curtails anticancer immunity.^26^ However, the role of fatty graft injury in inflammasome activation in MDSCs and on tumour recurrence has not been explored.

In the present study, we aimed to investigate the role and mechanism of inflammasome activation in MDSCs induced by acute-phase injury of steatotic liver graft and its subsequent effect on promoting tumour recurrence. Our data indicated that NLRP3 inflammasome in MDSCs was activated by arachidonic acid through FATP2 during fatty graft injury, and it further induced the IL-17 production of CD4^+^ T cells, promoting the tumour recurrence post liver transplantation.

## Materials and methods

### Clinical cohort and biopsies

Eighty-eight patients with HCC from 1997 to 2017 who underwent living-donor liver transplantation in Queen Mary Hospital, The University of Hong Kong, were included in our study. Among them, eight patients received fatty donor livers (fatty change >10%), whereas the other 80 patients received normal livers. The graft biopsies and peripheral blood were prospectively collected at 2 h and 7 days after portal vein reperfusion, respectively. Signed consent forms were acquired from each donor and recipient patient before operation. The procedures followed in the study conformed to the ethical standards of the Helsinki Declaration of 1975, as revised in 1983, and approved by the institutional review board of The University of Hong Kong.

### Animal models

Sprague Dawley rats (male, 6–8 weeks, 180–220 g) and C57 BL/6 mice (male, 6–8 weeks, 20–25 g) were purchased from the Laboratory Animal Unit, The University of Hong Kong. Both rats and mice were housed in a standard animal facility at 22 ± 2 °C under controlled 12-h light/dark cycles and had free access to chow and autoclaved water. The animals with poor physical conditions were excluded. All animals received humane care according to the criteria outlined in *Guide for the Care and Use of Laboratory Animals (National Institutes Health publication 86–23, 1985 revision)*. Experimental protocols were approved by the Committee on the Use of Live Animals in Teaching and Research, The University of Hong Kong.

#### Rat orthotopic liver transplantation model

The steatotic donor rats were fed with 45% high-fat diet (58G8, TestDiet, Land O’Lakes, USA), whereas the normal donor rats were fed with regular diet for 2 weeks (about 50% steatosis). The recipient rats were injected with carbon tetrachloride (CCl_4_, 2 ml/kg) s.c. for 4 weeks to induce liver cirrhosis before operation. The orthotopic liver transplantation model was established using small-for-size grafts (ratio of graft weight to recipient liver weight was about 50%) in Sprague Dawley rats. Blood and liver tissues were harvested at 2, 6, and 24 h after transplantation. The procedures were implemented according to the previous protocols.^27^^,^^28^

#### Mouse hepatic IRI and tumour recurrence model

The mice in the fatty liver group were fed with 45% high-fat diet for 3 weeks before operation. According to our preliminary experiment, five animals were chosen in each group (n = 5). All the animals were randomly grouping with no blinding. The animals with poor physical conditions were excluded. To mimic the graft injury, mice were subjected to liver IRI.^29^ The right triangular and right middle lobes (about 30%) of the liver underwent ischaemia for 45 min with a vascular clamp. Injured liver and spleen tissues were collected at 12 h after reperfusion. Lipofermata (an FATP2 inhibitor, 2 mg/kg, MedChemExpress, NJ, USA) was injected i.p. in the treatment group 1 day in advance and immediately before the operation start.

The liver tumour cells (Hepa1-6, ATCC, 1.5 × 10^6^/100 μl per mouse) were injected into the portal vein immediately after reperfusion to establish the mouse liver tumour recurrence model. The cell line has been authenticated. Lipofermata (2 mg/kg) was injected i.p. 1 day in advance, immediately before the operation, and every 2 days after the reperfusion in the treatment group. IL-1β recombinant protein (10 μg/kg; BioLegend, San Diego, CA, USA) was injected i.p. immediately after the reperfusion and every 2 days after the operation based on lipofermata treatment in the rescue group. At 14 days post operation, liver and spleen tissues were harvested for analysis. The tumour size was measured by the luminescent intensity after injection of luciferase (IVIS Spectrum Imaging System, PerkinElmer, Waltham, MA, USA).

### Primary MDSC and naive CD4^+^ T-cell isolation by magnetic bead cell sorting from mouse

Primary MDSCs and naive CD4^+^ T cells were isolated from the mouse bone marrow and spleen, respectively. Bone marrow cells were acquired from femurs and tibias. The splenic cells and bone marrow cells were flushed out using PBS, filtered through 70-μm cell strainers and treated with ammonium–chloride–potassium lysing buffer (Chem Cruz, Santa Cruz Biotechnology, Dallas, TX, USA). After obtaining the single cell suspension, Gr1^+^CD11b^+^ cells (MDSCs) and CD4^+^CD44^low^CD62L^high^ cells (naive CD4^+^ T cells) were isolated using mouse magnetic bead cell isolation kits (STEMCELL Technologies, Vancouver, BC, Canada) following the manufacturer’s instruction. The purity was >90% via flowcytometry analysis. The cells were cultured using charcoal stripped FBS (Gibco, NY, USA) medium. MDSCs were stimulated by arachidonic acid (10–200 μM, Sigma-Aldrich, Darmstadt, Germany) or treated with lipofermata (50 μM) for 16 h. After wash, MDSCs were seeded in the upper chambers of 24-well Transwell inserts (0.4 μm, polyester membrane; SPL, Korea). Naive CD4^+^ T cells, continuously stimulated with CD3/CD28 (25 μl/10^6^ cells/ml; Gibco) and along with IL-2 (30 U; R&D Systems, Minneapolis, MN, USA), were seeded in the lower chambers with/without IL-1β recombinant protein (200 ng/μl). Before checking the production of IL-17, CD4^+^ T cells were treated with a protein transport inhibitor (BD GolgiStop, BD Biosciences, San Jose, CA, USA) for 6 h.

### Laboratory methods

Further details on quantitative real-time PCR, flow cytometry analysis, immunostaining, RNA sequencing (RNA-seq), gas chromatography–mass spectrometry (GC-MS) for fatty acids, and Western blot can be found in the Supplementary Materials and Methods.

### Statistical analysis

Comparison was performed using Student’s *t* test. Clinical survival was analysed using the Kaplan–Meier test. Degrees of statistical significance were demonstrated using standardised asterisk nomenclature (∗*p* <0.05, ∗∗*p* <0.01, ∗∗∗*p* <0.001). All analyses were implemented using SPSS 18.0 (SPSS, Chicago, IL, USA) and GraphPad Prism 8.0 (GraphPad Software Inc, La Jolla, CA, USA).

## Results

### Increased MDSCs and NLRP3 were associated with more HCC recurrence after transplantation using steatotic liver graft

**Fig. 1:**
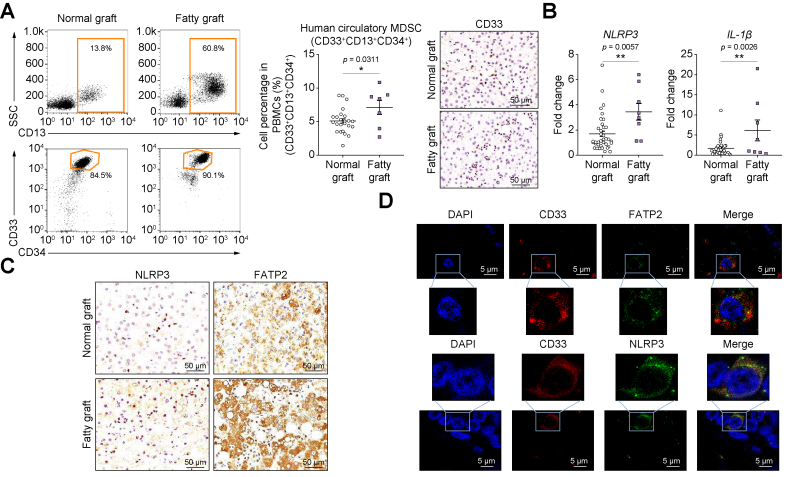
MDSC populations and NLRP3 levels were increased in patients with HCC who received fatty grafts post liver transplantation. (A) Circulatory and intragraft MDSCs were increased in patients who received fatty grafts compared with those with normal liver grafts (n = 45). Scale bars: 50 μm. (B) The mRNA expressions of NLRP3 and IL-1β were higher in fatty liver grafts (n = 31). (C) More infiltrated NLRP3- and FATP2-positive cells were found in patients who received fatty liver grafts (n = 23). Scale bars: 50 μm. (D) CD33 (a MDSC marker) was colocalised with FATP2 and NLRP3 (n = 23). Scale bars: 5 μm. Error bars indicate SEM; ∗*p* <0.05, ∗∗*p* <0.01. FATP2, fatty acid transport protein 2; HCC, hepatocellular carcinoma; MDSC, myeloid-derived suppressor cell; NLRP3, nucleotide-binding oligomerisation domain-like receptor family pyrin domain containing 3; PBMC, peripheral blood mononuclear cell; SSC: side scatter.

Eighty-eight patients with HCC who received living-donor liver transplantation were involved in this study. HCC recurrence occurred in 21 patients after transplantation. The survival analysis indicated that the recipients implanted with steatotic liver grafts had relatively poor disease-free survival compared with patients with normal grafts (*p* = 0.031) (Fig. S1A). The multivariate analysis further certified that fatty change influenced the tumour recurrence (*p* = 0.044) (Table S1). More circulatory (*p* = 0.0311) and intragraft MDSCs (CD33^+^, *p* <0.0001; CD15^+^ and LOX1^+^, *p* = 0.0005) were found in the patients with fatty grafts (Fig. 1A and Fig. S1B–D). Our previous study indicated that the patients with high MDSCs had poor disease-free survival.^18^ These findings suggested that MDSCs may play critical roles in tumour recurrence post liver transplantation using steatotic donors. Our previous report demonstrated that NLRP3 was the major type of inflammasome that was upregulated post transplantation.^23^ In the present study, the patients implanted with fatty grafts had higher mRNA levels of intragraft NLRP3 (*p* = 0.0057) and IL-1β (*p* = 0.0026) (Fig. 1B). More infiltrated NLRP3 (*p* = 0.0011) and FATP2 (*p* <0.0001) positive cells were detected in fatty liver grafts (Fig. 1C and Fig. S1E). The colocalisation and accumulation of NLRP3 and FATP2 with CD33 in fatty grafts demonstrated that NLRP3 and FATP2 might modulate MDSCs in post-transplant tumour recurrence (Fig. 1D and Fig. S1F).

### The aberrant lipid metabolism and tumour-favouring alteration in steatotic grafts were accompanied with higher MDSCs and NLRP3 post rat liver transplantation

**Fig. 2:**
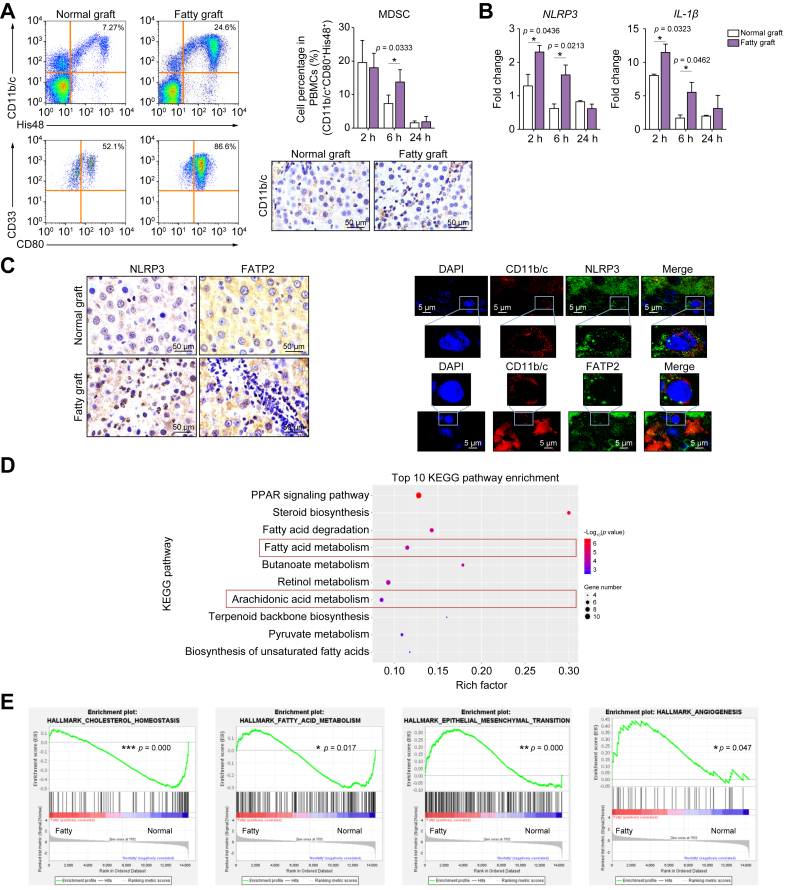
Higher MDSCs and NLRP3 were accompanied with aberrant lipid metabolism and tumour-favouring alteration in fatty grafts post rat liver transplantation. (A) More MDSCs were found in rats implanted with fatty liver grafts. Scale bars: 50 μm. (B) The mRNA expressions of NLRP3 and IL-1β were increased in fatty liver grafts. (C) NLRP3- and FATP2-positive cells were more infiltrated in steatotic grafts and colocalised with CD11b/c (an MDSC marker). Scale bars: 50 μm (left) and 5 μm (right). (D) The lipid metabolism (including arachidonic acid metabolism) was aberrant in fatty grafts through KEGG analysis of RNA-seq post transplantation. (E) Cholesterol homoeostasis, fatty acid metabolism, epithelial mesenchymal transition, and angiogenesis were significantly changed in steatotic grafts by GSEA. (A–C) n = 4/group; (D and E) n = 3/group. Error bars indicate SEM; ∗*p* <0.05, ∗∗*p* <0.01, ∗∗∗*p* <0.001. FATP2, fatty acid transport protein 2; GSEA, gene set enrichment analysis; KEGG, Kyoto Encyclopedia of Genes and Genomes; MDSC, myeloid-derived suppressor cell; NLRP3, nucleotide-binding oligomerisation domain-like receptor family pyrin domain containing 3; PBMC, peripheral blood mononuclear cell; RNA-seq, RNA sequencing.

To avoid the confounding factors in clinical cohorts and to further investigate the alteration of MDSCs, we established a rat orthotopic liver transplantation model using fatty or normal small-for-size grafts. Consistent with the clinical results, circulatory (*p* = 0.0333) and intragraft MDSCs (CD11b/c^+^, *p* = 0.0045; CD11b/c and His48, *p* = 0.0032) were significantly increased in the recipient rats with fatty donors at 6 h after reperfusion (Fig. 2A and Fig. S2A–C). By screening the inflammasome types, NLRP3 (*p* = 0.0213) and IL-1β (*p* = 0.0462) mRNA expressions were significantly upregulated in fatty grafts at Hour 6 post transplantation (Fig. 2B and Fig. S2D). More infiltrated NLRP3 (*p* = 0.0059) and FATP2 (*p* = 0.0067) positive cells and the colocalisation with CD11b/c (an MDSC marker) were found in rat fatty liver grafts (Fig. 2C and Fig. S2E and F). To further explore the different changes between normal and fatty grafts post transplantation, RNA-seq was performed. According to the Kyoto Encyclopedia of Genes and Genomes (KEGG) analysis, the top 10 pathways were related to lipid metabolism, including fatty acid and arachidonic acid metabolism (Fig. 2D). In addition to the significant change of cholesterol homoeostasis (*p* = 0.000) and fatty acid metabolism (*p* = 0.017), gene set enrichment analysis (GSEA) demonstrated that epithelial mesenchymal transition (*p* = 0.000) and angiogenesis (*p* = 0.047) were significantly enhanced in fatty grafts (Fig. 2E). These results indicated that aberrant lipid metabolism was critical in fatty grafts, which might promote the tumour-favouring microenvironment formation post transplantation.

### NLRP3 was upregulated in MDSCs with more lipid uptake in fatty liver of mouse IRI model

**Fig. 3:**
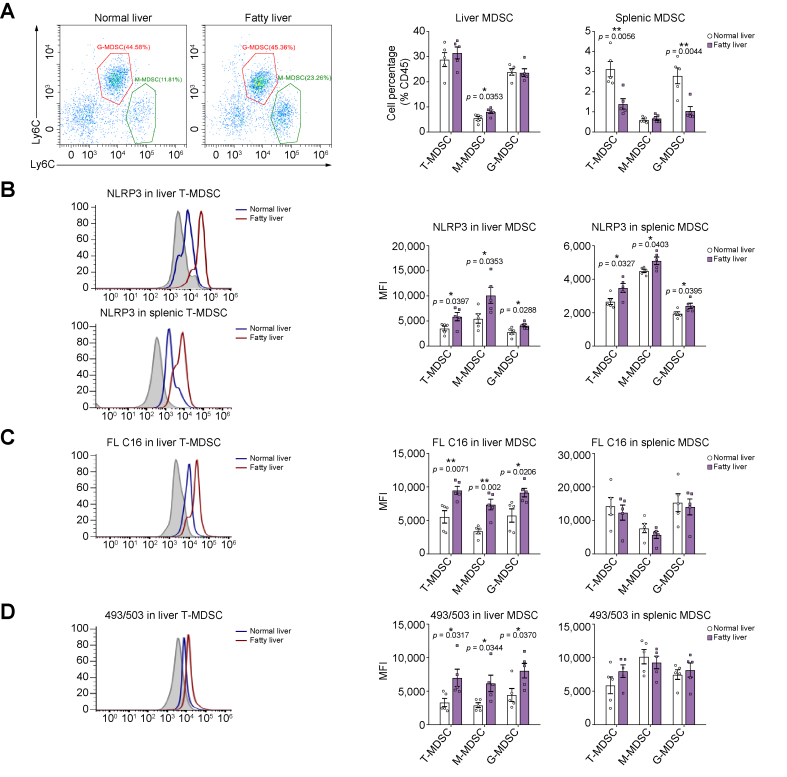
Accumulated lipids enhanced NLRP3 in MDSCs of mouse fatty liver post IRI. (A) More M-MDSCs were accumulated in mouse fatty liver after IRI. (B) NLRP3 was upregulated in liver and splenic MDSCs post fatty liver IRI. (C) More FL C16 was absorbed by MDSCs in fatty liver. (D) The neutral lipids (493/503) were accumulated in MDSCs of fatty liver post IRI. (A–D) n = 5/group. Error bars indicate SEM; ∗*p* <0.05, ∗∗*p* <0.01. G-MDSC, granulocytic MDSC; IRI, ischaemia/reperfusion injury; M-MDSC, monocytic MDSC; MDSC, myeloid-derived suppressor cell; MFI, mean fluorescence intensity; NLRP3, nucleotide-binding oligomerisation domain-like receptor family pyrin domain containing 3; T-MDSC, total MDSC.

The mouse hepatic IRI model with a fatty/normal liver was established to investigate the mechanism of NLRP3 activation in MDSCs. Briefly, the right triangular and right middle lobes of the liver underwent ischaemia for 45 min with a vascular clamp. Injured liver and spleen tissues were collected 12 h after reperfusion. The inflammatory markers of liver injury, including monocyte chemoattractant protein 1 (MCP-1), IL-6, C–X–C motif chemokine ligand 1 (CXCL1), interferon-γ (IFN-γ), IL-1β, and Toll-like receptor 4 (TLR4), were significantly upregulated in the fatty liver post IRI (Fig. S3A). In addition, alanine transaminase and aspartate aminotransferase were significantly increased in fatty liver after IRI (Fig. S3B). This verified the finding that the inflammatory injury was more severe in the fatty graft post liver transplantation. Regarding the MDSCs in the mouse liver IRI model, previous studies mainly focused on their mobilisation upon the surgical stress.^18^^,^^30^ Our preliminary data demonstrated that hepatic and splenic MDSCs were obviously accumulated post normal liver IRI in contrast with sham control, whereas NLRP3 levels in hepatic MDSCs were not significantly increased. In the current study, the hepatic NLRP3 (Hour 2: *p* = 0.0465; Hour 6: *p* = 0.0077) and IL-1β (Hour 2: *p* = 0.0482; Hour 6: *p* = 0.0141) mRNA levels were significantly upregulated at the early stage after fatty liver IRI (Fig. S3C). Interestingly, only monocytic MDSCs (M-MDSCs; *p* = 0.0353) were significantly accumulated in the fatty liver after IRI (Fig. 3A and Fig. S4A). Importantly, NLRP3 was increased in total MDSCs (T), M-MDSCs (M), and G-MDSCs (G) of the liver (T: *p* = 0.0397; M: *p* = 0.0353; G: *p* = 0.0288) and spleen (T: *p* = 0.0327; M: *p* = 0.0403; G: *p* = 0.0395) post fatty liver IRI (Fig. 3B). The lipid uptake was further detected in MDSCs. FL C16 was significantly increased in MDSCs (T: *p* = 0.0071; M: *p* = 0.002; G: *p* = 0.0206) of the fatty liver with no obvious change in the spleen (Fig. 3C). Moreover, MDSCs (T: *p* = 0.0317; M: *p* = 0.0344; G: *p* = 0.0370) accumulated more neutral lipids using 493/503 staining in the fatty liver post IRI (Fig. 3D). Dil-LDL and free fatty acid in MDSCs were not changed obviously during fatty liver IRI (Fig. S4B and C).

### Arachidonic acid activated NLRP3 inflammasome in MDSCs through FATP2

**Fig. 4:**
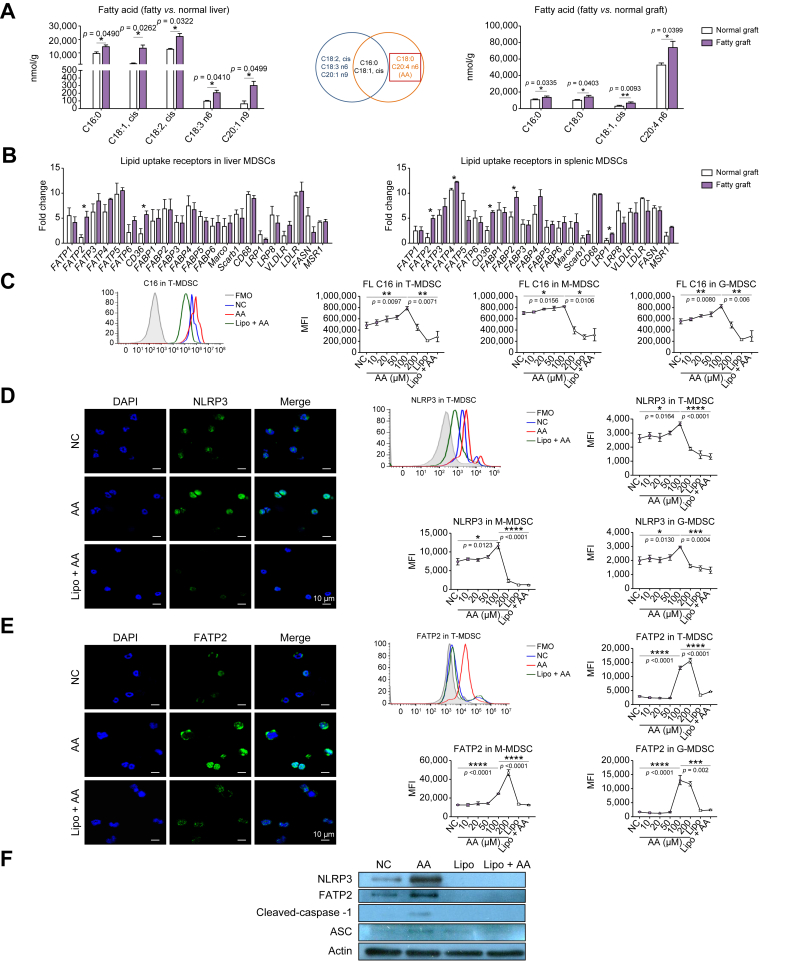
Arachidonic acid activated NLRP3 inflammasome in MDSCs through FATP2. (A) C18:0 and C20:4 n6 (AA) were screened out in rat fatty *vs*. normal liver graft after getting rid of the fatty acids as a result of the diet by GC-MS (n = 4/group). (B) FATP2 and CD36 mRNA levels were upregulated in both liver and splenic MDSCs post mouse fatty liver IRI (n = 5/group). (C) FL C16 was augmented by AA stimulation but inhibited by Lipo (an FATP2 inhibitor) in primary MDSCs. (D) AA increased NLRP3, which was reduced by Lipo in primary MDSCs. (E) FATP2 was accumulated in MDSCs by AA stimulation but decreased by Lipo. (F) FATP2 and NLRP3 activation pathway was enhanced by AA and inhibited by FATP2 blockade through Western blot analysis. Scale bars: 10 μm. Error bars indicate SEM; ∗*p* <0.05, ∗∗*p* <0.01, ∗∗∗*p* <0.001. AA, arachidonic acid; ASC, apoptosis-associated speck-like protein containing a caspase recruitment domain; FATP2, fatty acid transport protein 2; FMO, fluorescence minus one; G-MDSC, granulocytic MDSC; GC-MS, gas chromatography–mass spectrometry; IRI, ischaemia/reperfusion injury; Lipo, lipofermata; M-MDSC, monocytic MDSC; MDSC, myeloid-derived suppressor cell; MFI, mean fluorescence intensity; NC, negative control (the primary MDSCs without stimulation); NLRP3, nucleotide-binding oligomerisation domain-like receptor family pyrin domain containing 3; T-MDSC, total MDSC.

The fatty acids in fatty and normal grafts of the rat model were analysed by GC-MS. C18:0 and C20:4 n6 (arachidonic acid) were identified through screening after getting rid of the changed fatty acids as a result of the diet (Fig. 4A). To identify the ones that might mediate the activation of NLRP3 by fatty acids, the mRNA levels of potential lipid uptake receptors (as listed) in splenic and hepatic MDSCs isolated from the mouse model were evaluated. FATP2 and CD36 (two lipid uptake receptors) was increased in both liver and spleen MDSCs after fatty liver IRI in contrast with normal ones (Fig. 4B). To further explore the inflammasome activation by fatty acid, MDSCs were sorted from the bone marrow. FL C16 was found significantly accumulated in MDSCs (T: *p* = 0.0097; M: *p* = 0.0156; G: *p* = 0.0080) in a dosage-dependent manner with arachidonic acid stimulation (<100 μM). Lipofermata, an FATP2 inhibitor, could significantly reduce the arachidonic acid uptake of MDSCs (T: *p* = 0.0071; M: *p* = 0.0106; G: *p* = 0.006) (Fig. 4C). Moreover, NLRP3 levels in MDSCs were significantly increased by arachidonic acid stimulation (T: *p* = 0.0164; M: *p* = 0.0123; G: *p* = 0.0130) and decreased by lipofermata (T: *p* <0.0001; M: *p* <0.0001; G: *p* = 0.0004), as demonstrated by immunostaining and flow cytometry analysis (Fig. 4D). However, C18:0 could not change the levels of FL C16, NLRP3, and CD36 in a consistent manner, and sulfosuccinimidyl oleate sodium (an inhibitor of CD36) treatment could not decrease their expressions (Fig. S5A–C). These results indicated that arachidonic acid could be absorbed through FATP2 in MDSCs, which further activated the NLRP3 inflammasome.

FATP2 in MDSCs was also significantly upregulated by arachidonic acid stimulation (T: *p* <0.0001; M: *p* <0.0001; G: *p* <0.0001) and diminished by its inhibitor (T: *p* <0.0001; M: *p* <0.0001; G: *p* = 0.002) (Fig. 4E). Interestingly, CD36 in MDSCs was increased by arachidonic acid (T: *p* = 0.0147; M: *p* = 0.0405; G: *p* = 0.0104) and could be also reduced by lipofermata (T: *p* = 0.0017; M: *p* = 0.0051; G: *p* = 0.002) (Fig. S6A). The results from Western blot confirmed that the FATP2/NLRP3 inflammasome activation pathway could be enhanced by arachidonic acid and reduced by lipofermata (Fig. 4F). These data suggested that arachidonic acid could not only activate the NLRP3 inflammasome but also modulate the levels of lipid uptake receptors.

### Activation of NLRP3 inflammasome with mitochondrial dysfunction in MDSCs stimulated CD4^+^ T cells producing IL-17 *in vitro* and *in vivo*

**Fig. 5:**
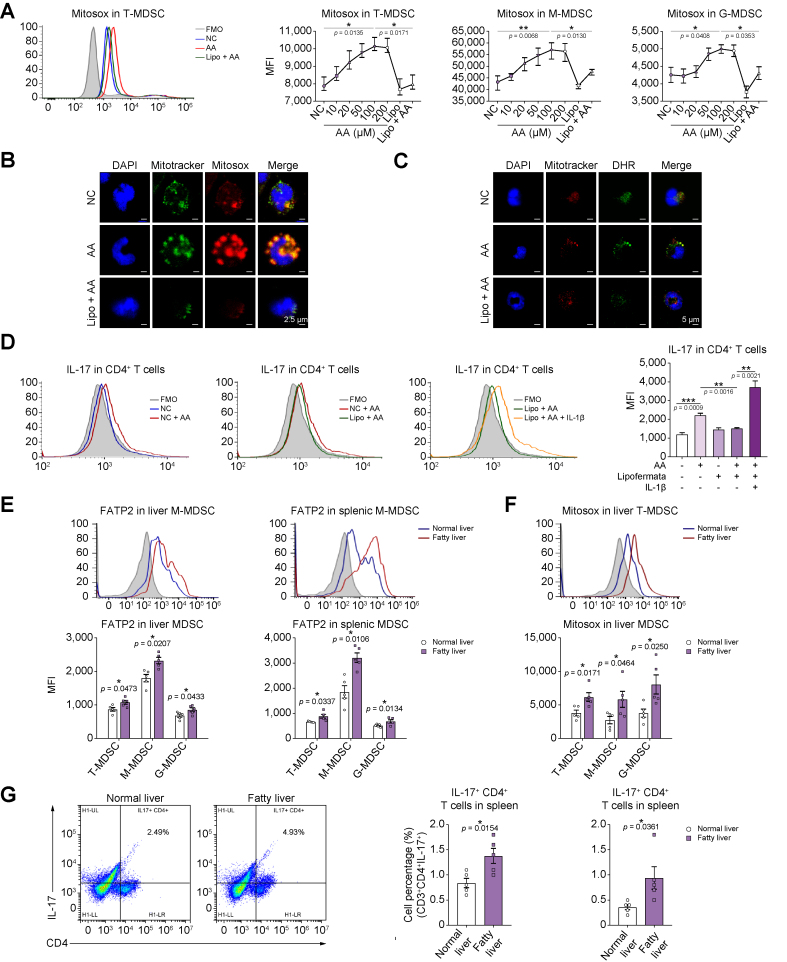
NLRP3 inflammasome activation with mitochondrial dysfunction in MDSCs stimulated naive CD4^+^ T cells producing IL-17. (A) AA increased the ROS (MitoSOX) of primary MDSCs, which was diminished by Lipo through flow cytometry analysis. (B) The MitoSOX was raised by AA and reduced by FATP2 inhibition by immunostaining. Scale bars: 2.5 μm. (C) DHR was enhanced and decreased by AA and Lipo, respectively. Scale bars: 5 μm. (D) The production of IL-17 in primary naive CD4^+^ T cells was increased, decreased, and restored after coculture with MDSCs treated by AA, Lipo, and IL-1β recombinant protein, respectively. (E) FATP2 in hepatic and splenic MDSCs was upregulated after fatty liver IRI in mice. (F) The ROS in MDSCs of the fatty liver was enhanced post IRI in mice. (G) The population of IL-17^+^ CD4^+^ T cells was accumulated after fatty liver IRI. (E–G) n = 5/group. Error bars indicate SEM; ∗*p* <0.05, ∗∗*p* <0.01, ∗∗∗*p* <0.001. AA, arachidonic acid; DHR, dihydrorhodamine 123; FATP2, fatty acid transport protein 2; FMO, fluorescence minus one; G-MDSC, granulocytic MDSC; IRI, ischaemia/reperfusion injury; Lipo, lipofermata; M-MDSC, monocytic MDSC; MDSC, myeloid-derived suppressor cell; MFI, mean fluorescence intensity; NC, negative control (the primary MDSCs without stimulation); NLRP3, nucleotide-binding oligomerisation domain-like receptor family pyrin domain containing 3; ROS, reactive oxygen species; T-MDSC, total MDSC.

Next, we explored the mechanisms of lipid uptake activating the NLRP3 inflammasome via FATP2 in MDSCs. The reactive oxygen species (ROS) production (mitochondrial superoxide indicator, MitoSOX), instead of mitochondrial counts (MitoTracker) in MDSCs, was significantly increased in a dosage-dependent manner (T: *p* = 0.0135; M: *p* = 0.0068; G: *p* = 0.0408). Moreover, the MitoSOX was decreased by lipofermata (T: *p* = 0.0171; M: *p* = 0.0130; G: *p* = 0.0353). Such phenotype was consistent with FL C16 and inflammasome expressions by arachidonic acid stimulation (Fig. 5A and Fig. S6B). The immunofluorescent staining further indicated that arachidonic acid enhanced the ROS of MDSCs, which might subsequently activate the NLRP3 inflammasome (Fig. 5B and C). The major mitochondrial protein levels, including NADH dehydrogenase (ubiquinone) 1 beta subcomplex subunit 8 (NDUFB8, complex I), succinate dehydrogenase B (SDHB, complex II), ubiquinol–cytochrome C reductase core protein 2 (UQCRC2, complex III), mitochondrially encoded cytochrome C oxidase I (MT-CO1, complex IV), ATP synthase alpha-subunit (ATP5A1, complex V), voltage-dependent anion channel (VDAC), prohibitin 1 (PHB1), and superoxide dismutase 1 (SOD1) in MDSCs were obviously downregulated by arachidonic acid stimulation but increased through the inhibition of FATP2. However, dynamin-related protein 1 (DRP1), the damage marker of mitochondria, was increased by arachidonic acid but reduced by FATP2 inhibition (Fig. S6C). Furthermore, we found that the ATPs produced by MDSCs were decreased by arachidonic acid (*p* = 0.0029) but enhanced by FATP2 blocking (*p* = 0.0004) (Fig. S6D). Therefore, the increased levels of ROS in MDSCs by arachidonic acid stimulation damaged the mitochondrial membrane and function, and diminished the production of ATPs.

The immune cell infiltration analysis from our RNA-seq data showed that CD4^+^ T cells were increased in steatotic grafts post transplantation. The effects of MDSCs on CD4^+^ T-cell differentiation were further explored. MDSCs were treated with arachidonic acid or lipofermata, and then cocultured with naive CD4^+^ T cells isolated from the mouse spleen. The IL-17 levels in CD4^+^ T cells were significantly increased (*p* = 0.0009) or reduced (*p* = 0.0016) after coculture with arachidonic acid- or lipofermata-treated MDSCs, respectively. Adding the IL-1β recombinant protein reversed the inhibition of lipofermata and raised much more IL-17 production of CD4^+^ T cells (*p* = 0.0021) (Fig. 5D and Fig. S6E). The data indicated that bioactive IL-1β release through inflammasome activation in MDSCs caused naive CD4^+^ T cells preferred to differentiate into T helper 17 (Th17) cells. An increase of Th17 cells was reported to promote HCC.^31^ Accumulated MDSCs and Th17 cells may lead to the microenvironment of the fatty liver graft favouring tumour recurrence post transplantation.

Based on our *in vitro* findings, the FATP2 levels and mitochondrial function in MDSCs were further verified in mice. Consistent with NLRP3, FATP2 was significantly increased in MDSCs of the liver (T: *p* = 0.0473; M: *p* = 0.0207; G: *p* = 0.0433) and spleen (T: *p* = 0.0337; M: *p* = 0.0106; G: *p* = 0.0134) after fatty liver IRI (Fig. 5E). The ROS in MDSCs was significantly enhanced (T: *p* = 0.0171; M: *p* = 0.0464; G: *p* = 0.0250), whereas the counts of mitochondria were not obviously changed in fatty liver after IRI, which was echoed with the *in vitro* findings (Fig. 5F and Fig. S6F and G). Moreover, IL-17-positive CD4^+^ T cells were significantly increased in the liver (*p* = 0.0154) and spleen (*p* = 0.0361) (Fig. 5G and Fig. S7A). CD36 in MDSCs was also upregulated in the liver (T: *p* = 0.0410; M: *p* = 0.0459; G: *p* = 0.0427) and spleen (T: *p* = 0.0034; M: *p* = 0.0004; G: *p* = 0.0405) (Fig. S7B). These data confirmed that NLRP3 inflammasome in MDSCs was activated through FATP2 with mitochondrial dysfunction and subsequently increased the IL-17 production of CD4^+^ T cells.

### Blockade of FATP2 inhibited NLRP3 activation in MDSCs and IL-17 production in CD4^+^ T cells post fatty liver IRI in mice

**Fig. 6:**
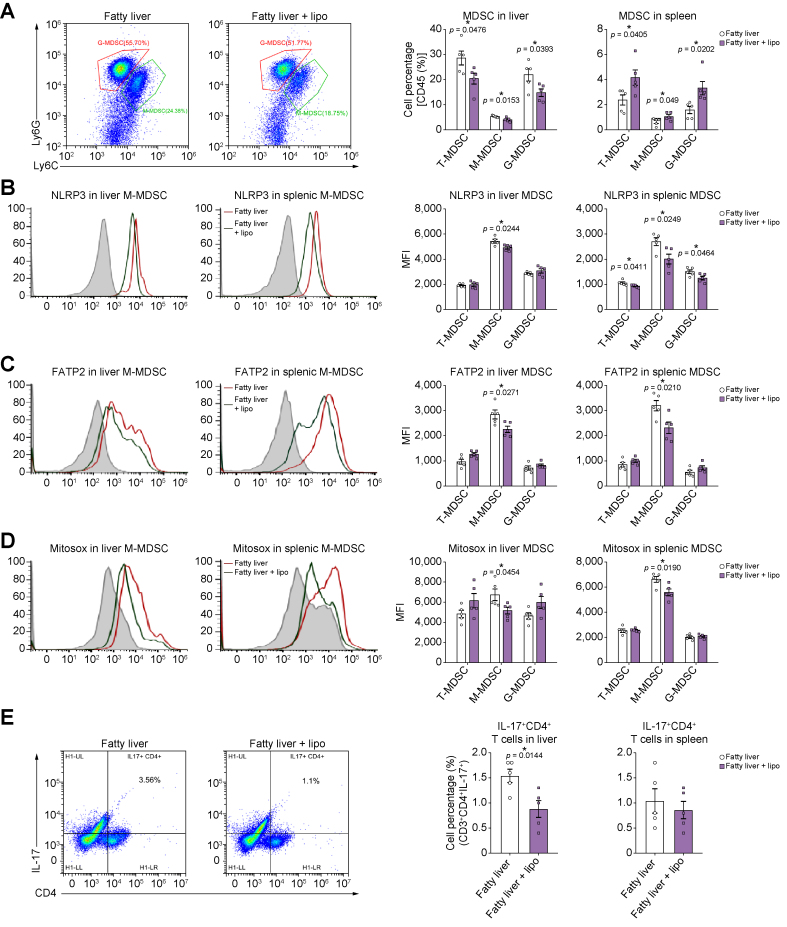
FATP2 blockade diminished NLRP3 activation in MDSCs and IL-17 production in CD4^+^ T cells after mouse fatty liver IRI. (A) The counts of hepatic MDSCs were reduced by FATP2 blockade after fatty liver IRI in mice. (B) NLRP3 in M-MDSCs of the fatty liver was decreased by FATP2 inhibition. (C) Lipo (an FATP2 inhibitor) reduced the FATP2 levels of M-MDSCs post fatty liver IRI. (D) ROS in M-MDSCs was diminished by FATP2 blockade. (E) The population of liver IL-17^+^CD4^+^ T cells was decreased by FATP2 inhibition. (A–E) n = 5/group. Error bars indicate SEM; ∗*p* <0.05. FATP2, fatty acid transport protein 2; G-MDSC, granulocytic MDSC; IRI, ischaemia/reperfusion injury; Lipo, lipofermata; M-MDSC, monocytic MDSC; MDSC, myeloid-derived suppressor cell; MFI, mean fluorescence intensity; NLRP3, nucleotide-binding oligomerisation domain-like receptor family pyrin domain containing 3; ROS, reactive oxygen species; T-MDSC, total MDSC.

As a lipid uptake receptor, FATP2 might be the target to reduce the NLRP3 activation in MDSCs. The MDSC populations in fatty liver were significantly decreased by lipofermata (an FATP2 inhibitor) treatment after IRI (T: *p* = 0.0476; M: *p* = 0.0153; G: *p* = 0.0393) (Fig. 6A and Fig. S4A). Inhibition of FATP2 reduced the NLRP3 levels only in M-MDSCs of the liver (*p* = 0.0244) and spleen (*p* = 0.0249) after fatty liver IRI (Fig. 6B). FATP2 in M-MDSCs was significantly diminished in both the liver (*p* = 0.0271) and the spleen (*p* = 0.0210) by lipofermata (Fig. 6C). CD36 expression was also downregulated in M-MDSCs of the liver (*p* = 0.0126) and spleen (*p* = 0.0419) (Fig. S7C). Consistently, FATP2 inhibition reduced the ROS in M-MDSCs of the liver (*p* = 0.0454) and spleen (*p* = 0.0190) (Fig. 6D). Furthermore, the population of IL-17-positive CD4^+^ T cells in the fatty liver was significantly decreased by blockade of FATP2 (*p* = 0.0144) (Fig. 6E). These results suggested that FATP2 inhibition could effectively decrease NLRP3 inflammasome activation in MDSCs and IL-17 production in CD4^+^ T cells during fatty liver IRI, which paved the way to study the effects of FATP2 blockade on tumour recurrence post transplantation.

### Tumour recurrence was inhibited by therapeutic targeting of FATP2

**Fig. 7:**
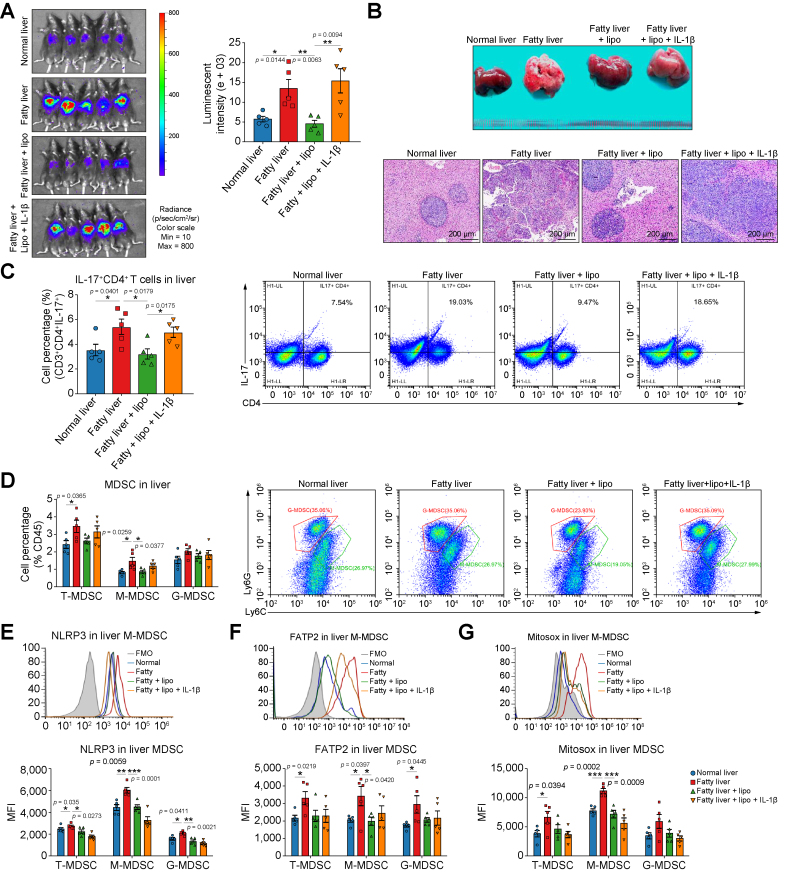
Targeting FATP2 inhibited liver tumour recurrence in mice. (A) The tumour size was increased in the fatty liver, and decreased and restored by Lipo and IL-1β recombinant protein injection, respectively. (B) FATP2 blockade inhibited the increased tumour size in the fatty liver, whereas IL-1β injection offset the inhibition effects in the mouse tumour recurrence model. (C) IL-17^+^ CD4^+^ T cells were accumulated in the fatty liver, and reduced and raised by Lipo and IL-1β, respectively. (D) The population of liver M-MDSCs was increased in the fatty liver and decreased by FATP2 blockade. (E) NLRP3 in liver MDSCs was upregulated in the fatty liver and reduced by FATP2 inhibition. (F) FATP2 in hepatic MDSCs was enhanced in the fatty liver, whereas Lipo inhibited its levels in M-MDSCs. (G) The ROS in liver M-MDSCs was increased in the fatty liver and diminished by FATP2 inhibition. (A–G) n = 5/group. Error bars indicate SEM; ∗*p* <0.05, ∗∗*p* <0.01, ∗∗∗*p* <0.001. FATP2, fatty acid transport protein 2; FMO, fluorescence minus one; G-MDSC, granulocytic MDSC; Lipo, lipofermata; M-MDSC, monocytic MDSC; MDSC, myeloid-derived suppressor cell; MFI, mean fluorescence intensity; NLRP3, nucleotide-binding oligomerisation domain-like receptor family pyrin domain containing 3; ROS, reactive oxygen species; T-MDSC, total MDSC.

To further explore the effects of targeting FATP2, mouse hepatic IRI with tumour recurrence models (the liver tumour cells were injected into the portal vein immediately after reperfusion) were established with four treatment groups: (1) normal liver, (2) fatty liver, (3) fatty liver + lipofermata, and (4) fatty liver + lipofermata + IL-1β recombinant protein. The tumour size was significantly increased (*p* = 0.0144) in the fatty liver and reduced by lipofermata treatment (*p* = 0.0063). IL-1β offset the inhibition of lipofermata and expanded the tumour size through evaluating the luminescent intensity (*p* = 0.0094) (Fig. 7A and B). Moreover, the increased liver weight/body weight and spleen weight/body weight ratios in the fatty liver group were suppressed by FATP2 inhibition and raised by IL-1β (Fig. S8A). The immunohistochemistry staining showed that alpha foetoprotein and CD31-positive cells were accumulated in the fatty liver group, decreased by FATP2 inhibition and obviously increased by IL-1β treatment. These results demonstrated that not only the tumour burden but also the angiogenesis was increased in the fatty liver, and both were inhibited by FATP2 blockade and restored by IL-1β (Fig. S8B). Consistently, the hepatic IL-17^+^CD4^+^ T cells were significantly accumulated (*p* = 0.0401) in the fatty liver group, decreased by FATP2 inhibition (*p* = 0.0179) and obviously increased by IL-1β (*p* = 0.0175) (Fig. 7C and Figs. S7A and S9A). Only the hepatic M-MDSCs were significantly increased (*p* = 0.0259) in the fatty liver but were decreased by FATP2 blockade (*p* = 0.037) (Fig. 7D and Fig. S9B and C). Moreover, NLRP3 in MDSCs was enhanced in both the liver (T: *p* = 0.035; M: *p* = 0.0059; G: *p* = 0.0411) and spleen (T: *p* = 0.0296; M: *p* = 0.0269) of the fatty liver group. Lipofermata reduced NLRP3 in MDSCs of the liver (T: *p* = 0.0273; M: *p* = 0.0001; G: *p* = 0.0021) and spleen (T: *p* = 0.0466; M: *p* = 0.0269; G: *p* = 0.0053). IL-1β recombinant protein inhibited NLRP3 more in MDSCs probably owing to the feedback of IL-1β (Fig. 7E and Fig. S9D).

Consistent with NLRP3, FATP2 in MDSCs was upregulated in both the liver (T: *p* = 0.0219; M: *p* = 0.0397; G: *p* = 0.0445) and spleen (T: *p* = 0.0063; M: *p* = 0.0433; G: *p* = 0.0033) of the fatty liver group. However, FATP2 was inhibited by lipofermata only in M-MDSCs of the liver (*p* = 0.0420) and spleen (*p* = 0.0217) (Fig. 7F and Fig. S9E). The ROS in MDSCs was also upregulated in the liver (T: *p* = 0.039; M: *p* = 0.0002) and spleen (T: *p* = 0.0258; M: *p* = 0.0175; G: *p* = 0.0293) of the fatty liver group. Consistently, only the ROS in hepatic M-MDSCs was inhibited by FATP2 blockade (*p* = 0.0009) (Fig. 7G and Fig. S9F). In the fatty liver group, CD36 in M-MDSCs was enhanced in the liver (*p* = 0.0069) and spleen (*p* = 0.0233). FATP2 inhibition could significantly reduce the CD36 levels in hepatic MDSCs (T: *p* = 0.0347; M: *p* = 0.0175; G: *p* = 0.0429) (Fig. S9G). FATP2 levels in liver tumour are lower than those in non-tumour liver tissues in the Gene Expression Profiling Interactive Analysis database, and the viability of tumour cells (Hepa1-6) was not obviously affected by lipofermata in our preliminary data. Therefore, these results excluded the effects of lipofermata on tumour cells. These data suggested that FATP2 blockade could reduce the tumour recurrence through suppressing the activation of NLRP3 inflammasome in MDSCs, which further inhibited the IL-17 production of CD4^+^ T cells.

### Discussion

In the current study, we demonstrated that the inflammasome activation in MDSCs bridged the acute-phase steatotic liver graft injury and tumour recurrence. Our finding provided the new insight that fatty liver graft injury induced the immunosuppressive environment, which contributed to tumour recurrence after transplantation. Our study indicated the casual effect of inflammation induced by steatotic liver graft injury on tumour recurrence from the perspective of immunosuppressive environment formation post transplantation.

We first identified that arachidonic acid activated NLRP3 inflammasome in MDSCs post transplantation using fatty grafts. MDSCs represent a heterogeneous population of myeloid progenitor cells, whose immune function was influenced by lipid metabolism.^32^ For example, the uptake of triacylglycerol substrates and subsequent lipolysis are essential for M2 activation.^33^ The lipid accumulation in dendritic cells impairs their ability to process and present antigens in tumour.^34^ The fatty acid uptake reprogrammes the neutrophils into G-MDSCs to promote tumour progression.^17^ However, previous studies mainly focused on the role of lipid metabolism promoting the immunosuppressive phenotype of MDSCs.[15], [16], [17] Our current study first explored the mechanism for lipid metabolism on inflammasome activation in MDSCs. Arachidonic acid was firstly found to activate inflammasome in the current study, although other lipids have been reported, including palmitic acid.^35^ The alteration of arachidonic acid metabolism was found by both GC-MS and RNA-seq analysis in our study, suggesting its critical role in steatotic liver graft injury. It was recently reported that arachidonic acid also induced the immunosuppressive activity acquisition of neutrophils.^17^ The accumulation of arachidonic acids in hepatic MDSCs could enhance NLRP3 and FATP2 in both liver and splenic MDSCs. It indicated the significant impact of fatty acid metabolism alteration in hepatic MDSCs. The detailed alterations of lipid metabolism pathways are worthwhile for further independent studies.

The fatty liver graft injury was associated with impairment of hepatic microcirculation and dysfunction of mitochondria.^4^^,^[36], [37], [38] NLRP3 inflammasome activation was found to be associated with mitochondrial dysfunction in the present study. The hepatic IRI induced the mitophagy and permeability change of mitochondria.^6^ Our recent study also demonstrated that a compromised AMPK–PGC1α axis exacerbated steatotic liver graft injury by dysregulating mitochondrial homoeostasis.^4^ The dysfunction of mitochondria produced excessive ROS, resulting in the oxidative stress and cytotoxicity.^39^ Mitochondrial DNA release was demonstrated as the link between palmitic acid stimulation and inflammasome activation.^35^ The previous reports showed that succinate was a universal metabolic signature of ischaemia in transplanted organs (including the liver) and is responsible for mitochondrial ROS production during reperfusion at complex I although there are controversial findings in humans.^40^^,^^41^ In the current study, ROS was increased in MDSCs by arachidonic acid uptake through FATP2 in the fatty graft post liver transplantation. We found that the complexes of mitochondria were impaired with reduced levels of ATP by arachidonic acid stimulation, but the effects could be rescued by FATP2 inhibition. Our finding might contribute to the mechanism of fatty acid metabolism on mitochondrial dysfunction in MDSCs.

FATP2, as the most critical member of the FATP family in the liver, is identified as a major contributor to peroxisomal very-long-chain acyl-CoA synthetase and hepatic fatty acid uptake.^42^ In our study, FATP2 was found as the transporter that mediated arachidonic acid uptake. The enhanced CD36, the classical lipid uptake receptor, was also reduced by FATP2 blockade, which demonstrated its critical role in lipid uptake. Interestingly, FATP2 on M-MDSCs was obviously higher than FATP2 on G-MDSCs, which might explain that NLRP3 activation in M-MDSCs was more sensitive by FATP2 inhibition. Moreover, the hepatic M-MDSCs were distinctly increased in fatty liver IRI. The previous findings reported that FATP2 blockade could abrogate the activity of G-MDSCs and substantially delayed tumour progression.^17^ The mechanisms of FATP2 overexpression on M-MDSCs found in the current study need to be further explored. Based on our findings, the treatment of FATP2 inhibitors before and after transplantation for the recipients decreased the harmful effects of aberrant fatty acid metabolism on liver graft immune environment during acute injury. We previously also found that inhibiting lipocalin-2 could attenuate fatty liver graft injury.^43^ Our recent study showed that metformin could alleviate steatotic liver graft injury by restoring mitochondrial function through AMPK reactivation.^4^ Probably, the combination therapy or personalised treatments targeting at FATP2 and lipocalin-2, together with metformin, might be the solutions to attenuate steatotic graft injury and subsequently reduce cancer recurrence after transplantation. Moreover, combine machine perfusion and FATP2 inhibitor might be the direction to the clinical practice owing to the observed effect of hypothermic machine perfusion on HCC recurrence in livers from donation after circulatory death and the overall protection from complications and graft loss in recipients of steatotic livers from deceased donors.^44^^,^^45^

As more primitive cells, more infiltrated M-MDSCs with higher FATP2 might have profound impact on the immune microenvironment, such as T cells. In the current study, the inflammasome activation by arachidonic acid produced more bioactive IL-1β, which skewed the naive CD4^+^ T cells differentiated into Th17 cells. This was consistent with the report that the chemotherapy triggered inflammasome activation in MDSCs, which induced the IL-17 secretion of CD4^+^ T cells.^26^ Th17 cells not only can switch to a Th1 phenotype and exert antitumour activities but also are capable of expressing a regulatory T cell phenotype, which can perform protumour and antitumour activities, depending on the context of the immune response.^46^^,^^47^ In HCC, accumulation of intratumour Th17 cells promote tumour progression through fostering angiogenesis, and associated with poor survival in patients.^31^ The mechanism of Th17 promoting liver tumour progression is not well understood. One study demonstrated that IL-17 has a direct effect over HCC with the induction of IL-6/Janus kinase 2 (JAK2)/signal transducer and activator of transcription 3 (STAT3) by activating the protein kinase B (AKT) pathway.^48^ The STAT3 signalling promotes tumour growth through the regulation of pro-angiogenic genes,^49^ which is echoed by our findings that angiogenesis was significantly enhanced in fatty grafts post liver transplantation. Our previous data showed that the counts of MDSCs were obviously increased after hepatic IRI, and the current study demonstrated that fatty liver grafts recruited more M-MDSCs. Therefore, accumulated MDSCs and the higher inflammasome activation co-ordinately induced more IL-17 production of naive CD4^+^ T cells, contributing to the impairment of tumour surveillance. The recruitment of M-MDSCs to fatty liver grafts might be associated with more bioactive IL-1β release as a result of the inflammasome activation.

Taken together, arachidonic acid activated NLRP3 inflammasome in MDSCs through FATP2 during fatty liver graft injury, which led to more IL-17 secretion of CD4^+^ T cells and promoted tumour recurrence post transplantation through our integrated study with clinical analyses, animal models, and *in vitro* experiments. Targeting FATP2 represents the novel therapeutic strategies to reduce tumour recurrence post liver transplantation and to potentially expand the donor pool.

## Financial support

This work was supported by the General Research Funding (GRF: 17106921&17124219), Theme-based Research Scheme (TRS: T12-703/19R), and Collaborative Research Funding (C7026-18 GF&C7021-21G) from the Research Grant Council, Hong Kong. This work was also supported by the 10.13039/501100001809National Science Foundation of China (NSFC) grants (82203794 and 82072757).

## Authors’ contributions

Designed the study, performed the experiments and interpretation of data, and wrote/revised the manuscript: HL, KM. Obtained fundings and supervised the study: KM, CL. Was involved in technical support: HL, WY, LP, JL, XL, KN, QZ, WQ, YZ, TD, ZW, JZ. Had final approval of the submitted version: all authors.

## Data availability statement

The data generated in this study are available upon request from the corresponding author. The RNA-seq data have been submitted and are available through the NCBI’s Gene Expression Omnibus (GEO GSE204919).

## Conflicts of interest

All authors have no conflicts of interest to be declared.

Please refer to the accompanying ICMJE disclosure forms for further details.
