# Supplementary material for: Arachidonic acid activates NLRP3 inflammasome in MDSCs via FATP2 to promote post-transplant tumour recurrence in steatotic liver grafts
Source: JHEP Rep. 2023 Aug 22;5(12):100895. doi: 10.1016/j.jhepr.2023.100895 (PMC10616418; doi:10.1016/j.jhepr.2023.100895)
Supplement: Multimedia component 1 [file mmc1.pdf]

Arachidonic acid activates NLRP3 inflammasome in MDSCs via FATP2 to promote post-transplant tumor recurrence in steatotic liver grafts

Hui Liu, Wai Ho Oscar Yeung, Li Pang, Jiang Liu, Xiao Bing Liu, Kevin Tak Pan Ng, Qingmei Zhang, Wen Qi Qiu, Yueqin Zhu, Tao Ding, Zhe Wang, Ji Ye Zhu, Chung Mau Lo, Kwan Man

Table of contents

Supplementary figures……………………………….............…………………………2

Supplementary tables………………………………………….............…………….… 11

Supplementary figure legends…………………………………………...........……...14

Supplementary materials and methods………………………………………........……19

Supplementary references..22

Supplementary figures

Fig. S1

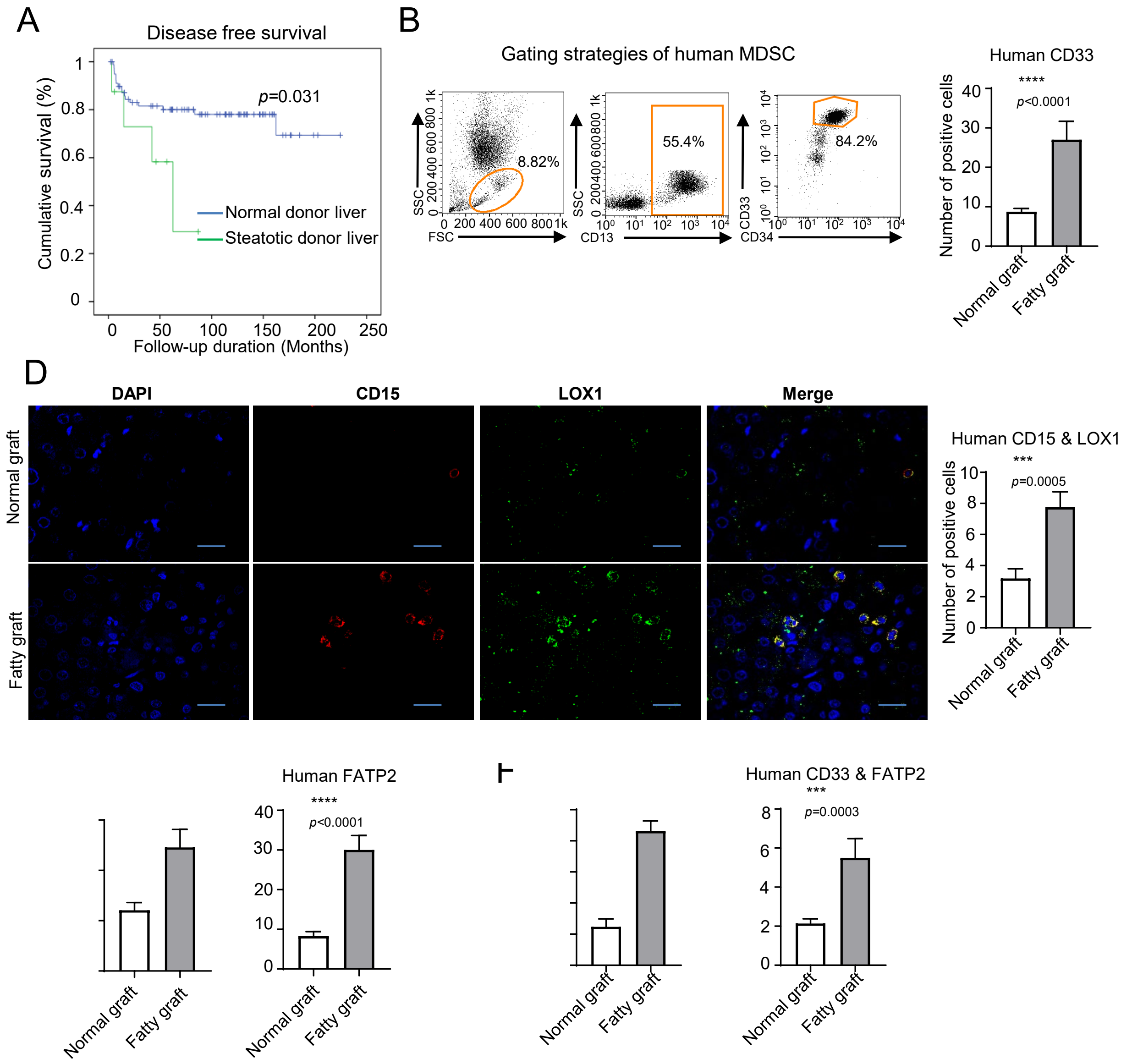

Fig. S1 The tumor recurrent rate was higher in HCC patients underwent liver transplantation using steatotic donor accompanied with the increase of MDSCs and NLRP3/FATP2 levels. A The tumor recurrence free survival was relatively poor in HCC patients after liver transplantation using steatotic donor (n=88). **B** The gating strategies of human MDSCs (CD33$^+$CD13$^+$CD34$^+$) by flow cytometry (n=45). **C** The number of MDSCs (CD33$^+$) were significantly accumulated in steatotic grafts by immunohistochemistry (n=23). **D** More MDSCs were infiltrated in fatty grafts by co-staining of CD15 & LOX1 (n=23). Scale bars: 20μm. **E** The increased numbers of NLRP3 and FATP2 positive cells in fatty grafts by immunohistochemistry (n=23). **F** More CD33$^+$NLRP3$^+$ and CD33$^+$FATP2$^+$ cells were infiltrated in fatty grafts post liver transplantation by co-staining (n=23). Error bars indicate Standard Error of Mean; **p<0.05, **p<0.01, ***p<0.001, ****p<0.0001.* MDSC, myeloid-derived suppressor cell; NLRP3: nucleotide-binding oligomerization domain-like receptor family pyrin domain containing 3; FATP2: fatty acid transport protein 2.

Fig. S2

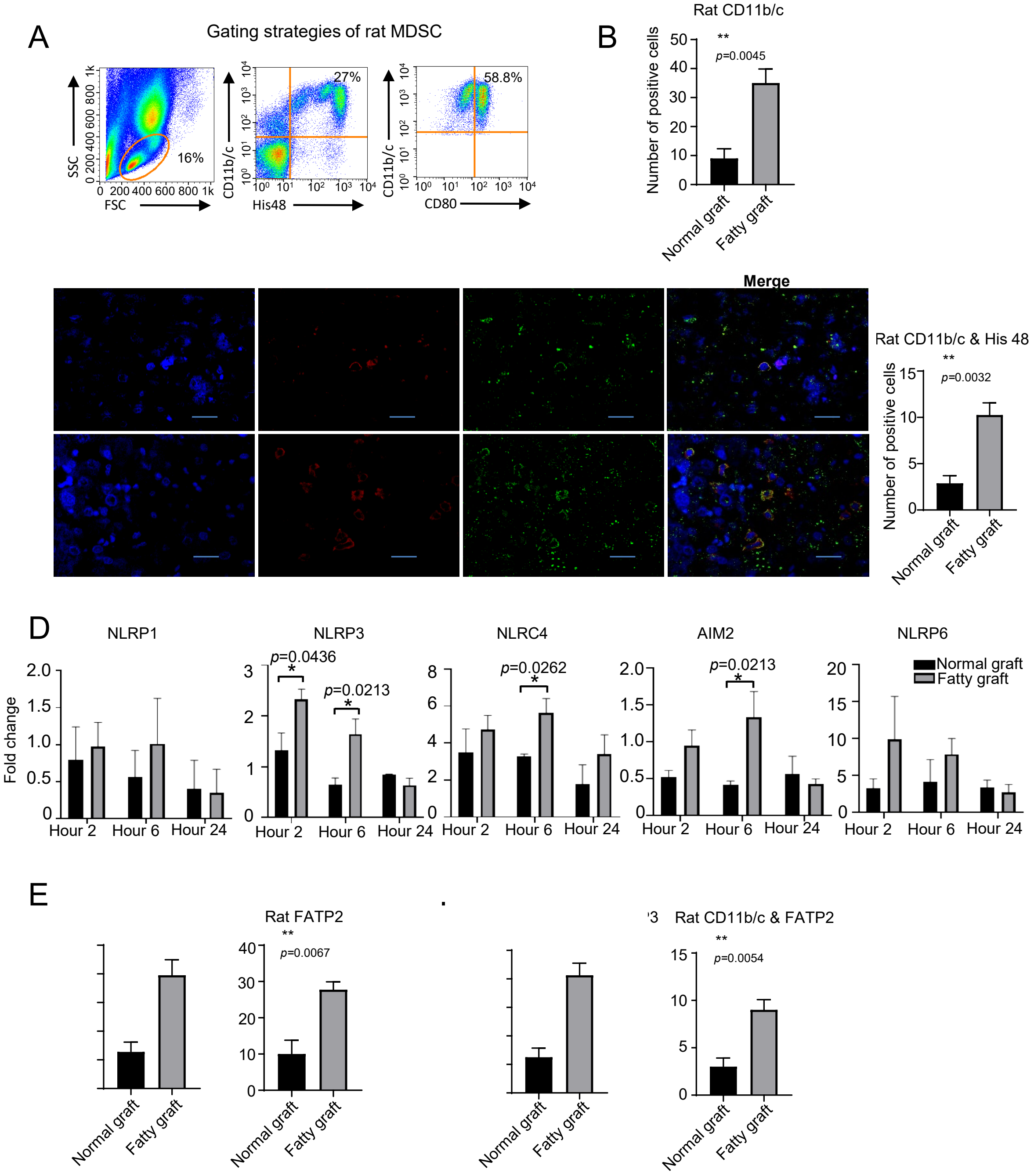

Fig. S2 The levels of NLRP3 and FATP2 were higher with accumulation of MDSCs in fatty liver grafts of rat model. A The gating strategies of rat MDSCs ($CD11b/c^{+}CD80^{+}His48^{+}$) by flow cytometry. **B** The number of $CD11b/c^{+}$ (MDSC marker) cells were obviously increased in rat fatty liver grafts by immunohistochemistry. **C** Accumulated MDSCs in fatty grafts by co-staining of CD11b/c & His48. Scale bars: 20μm. **D** NLRP3 was upreglated in fatty grafts through the inflammsome (NLRP1, NLRP3, NLRC4, AIM2, NLRP6) mRNA screen of rat liver transplantation model. **E** NLRP3 and FATP2 positive cells were more in fatty liver grafts by immunohistochemistry. **F** The immunofluorescent co-staining showed increased $CD11b/c^{+}NLRP3^{+}$ and $CD11b/c^{+}FATP2^{+}$ cells in fatty grafts post liver transplantation. A-F: n=4/group; Error bars indicate Standard Error of Mean; **p<0.05, **p<0.01, ***p<0.001, ****p<0.0001.* MDSC, myeloid-derived suppressor cell; NLRP: nucleotide-binding oligomerization domain-like receptor family pyrin domain containing; FATP2: fatty acid transport protein 2; NLRC4: NLR Family CARD Domain Containing 4; AIM2: Absent In Melanoma 2.

Fig. S3

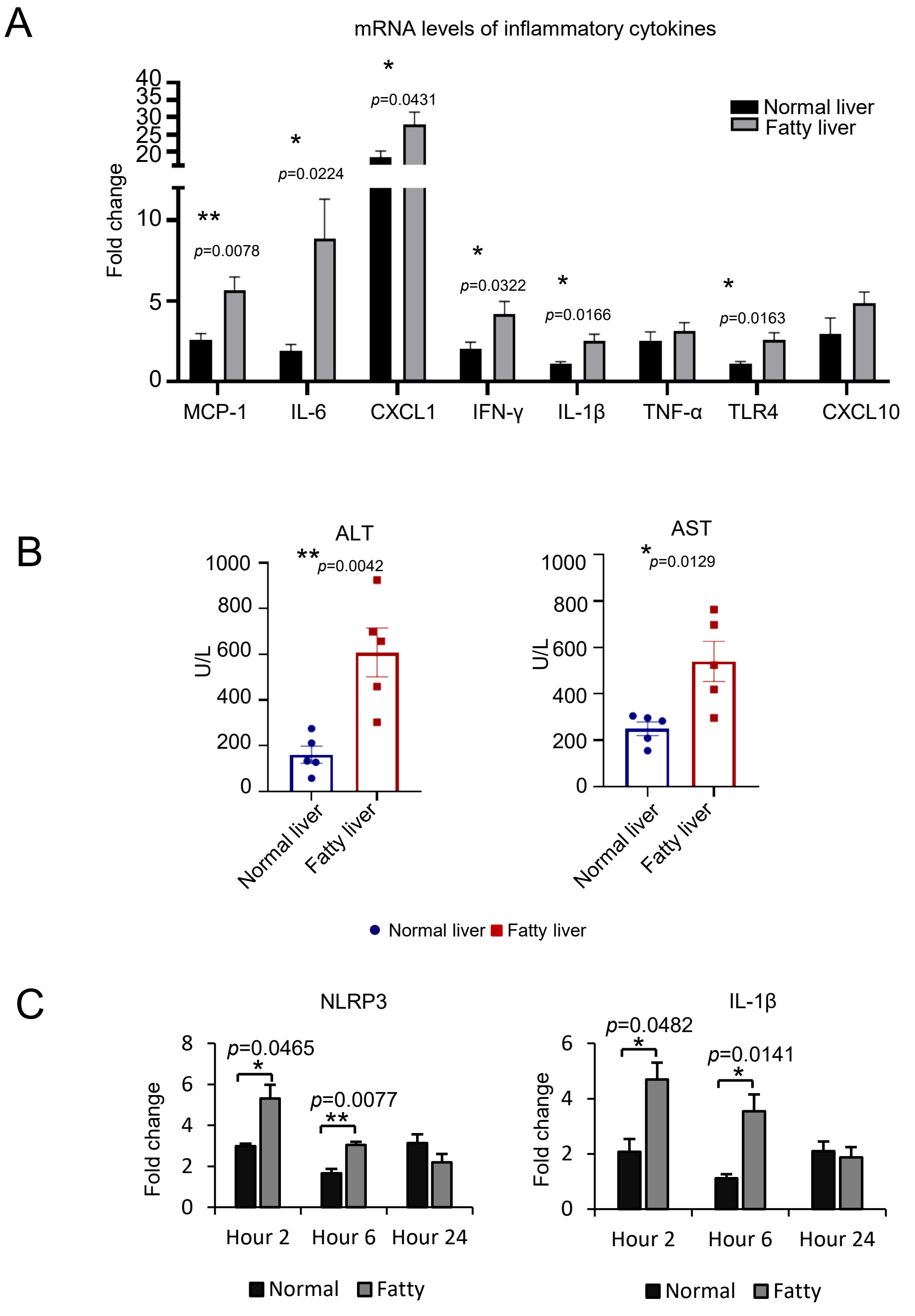

Fig. S3 The severer inflammatory injury and poorer liver function with enhanced NLRP3 and IL-1β expressions in mice post fatty liver ischemia/reperfusion injury (IRI). A The inflammatory markers, included MCP-1, IL-6, CXCL1, IFN-γ, IL-1β and TLR4, were significantly upregulated post fatty liver IRI. **B** The liver function was poorer with increased alanine transaminase (ALT) and aspartate aminotransferase (AST) in fatty liver after IRI. **C** The mRNA levels of NLRP3 and IL-1β were increased in fatty liver post IRI in mice**.** A-C: n=5/group; Error bars indicate Standard Error of Mean; **p<0.05, **p<0.01.* NLRP3: nucleotide-binding oligomerization domain-like receptor family pyrin domain containing 3; IRI, ischemia/reperfusion injury; MCP-1: Monocyte Chemoattractant Protein 1; IL-6: Interleukin-6; CXCL1: C-X-C Motif chemokine ligand 1; IFN-γ: Interferon-γ; TNF-α: tumor necrosis factor α; TLR4: Toll Like Receptor 4; ALT: alanine transaminase; AST, aspartate aminotransferase.

Fig. S4

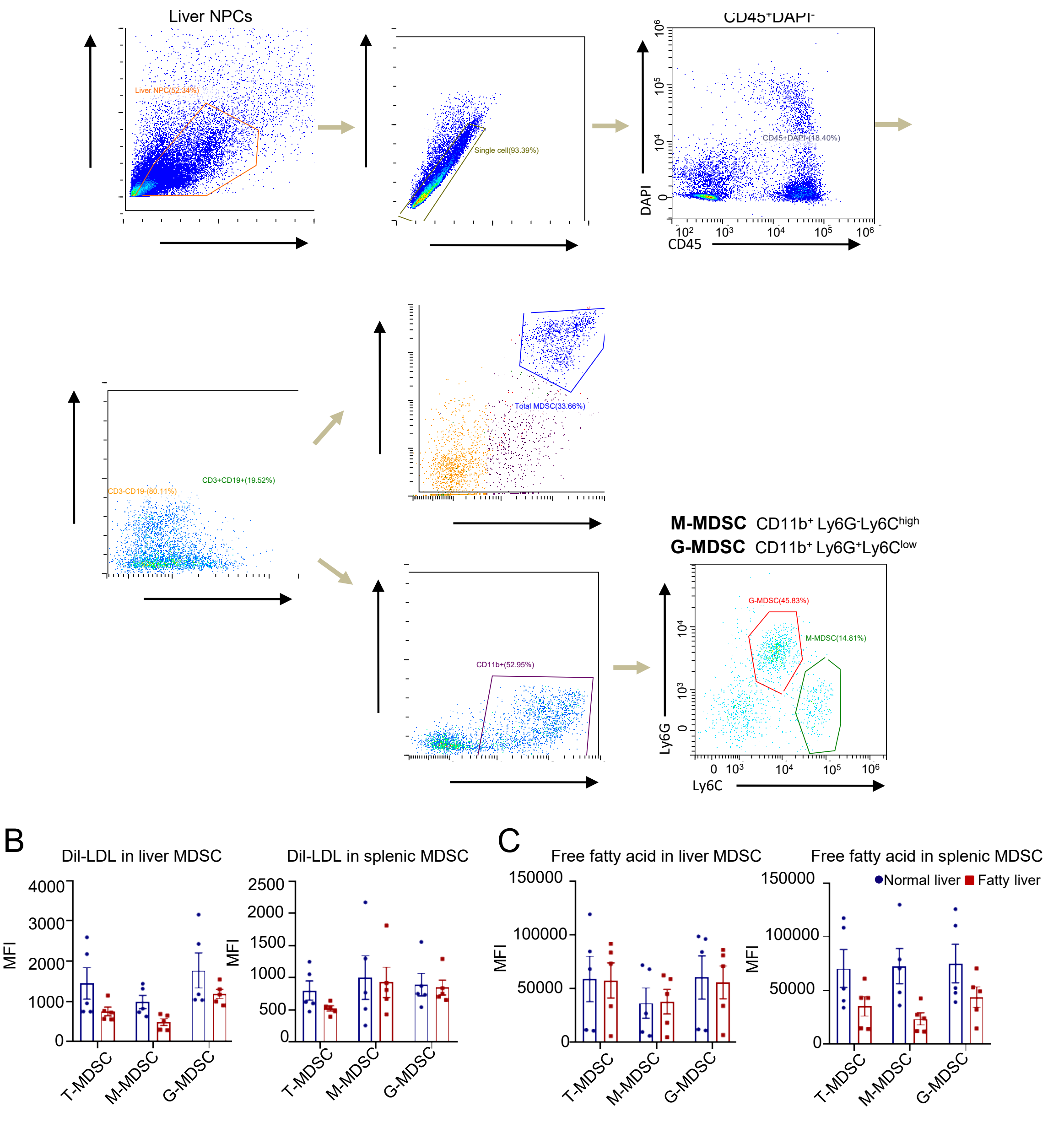

Fig. S4 No obvious change of Dil-LDL and free fatty acid in MDSCs of mice post fatty liver IRI. A The gating strategies of Total MDSC (T-MDSC, $CD11b^{+}Gr1^{+}$), Granulocytic MDSC (G-MDSC, $CD11b^{+}$ $Ly6G^{+}Ly6C^{low}$) and Monocytic MDSC (M-MDSC, $CD11b^{+}$ $Ly6G^{-}Ly6C^{high}$) in mice. **B** Dil-LDL in MDSCs was not significantly changed in mice after fatty liver IRI. **C** Free fatty acids were not altered obviously post fatty liver IRI. A-C: n=5/group; Error bars indicate Standard Error of Mean; *$*p<0.05$, $**p<0.01$*. IRI, ischemia/reperfusion injury; MDSC, myeloid-derived suppressor cell; LDL: low density lipoprotein; T-MDSC: total-MDSC; M-MDSC: monocytic-MDSC; G-MDSC: granulocytic-MDSC.

Fig. S5

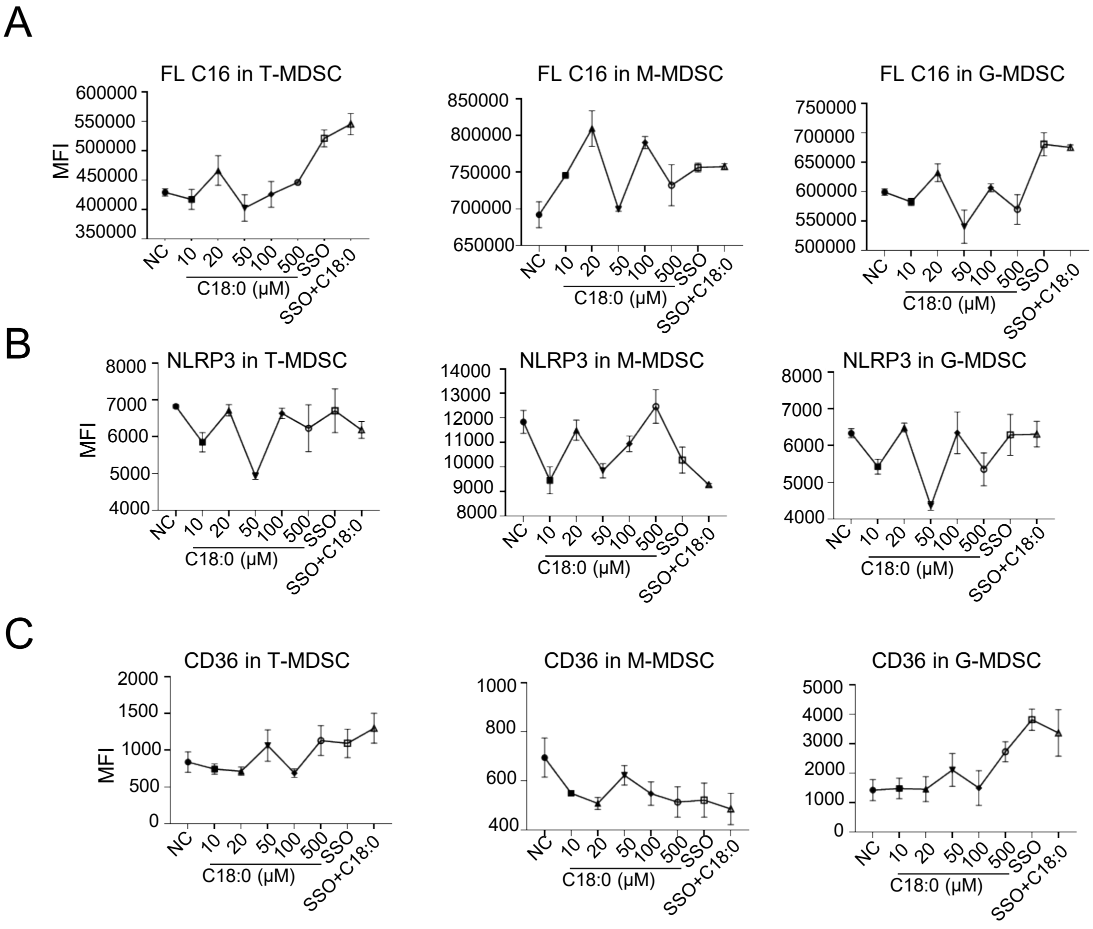

Fig. S5 NLRP3 in primary MDSCs was not changed by C18:0 stimulation. A FL C16 in primary MDSCs was not obviously altered by C18:0 or SSO (CD36 inhibitor) treatment. **B** C18:0 or SSO could not activate or inhibit NLRP3 in MDSCs, respectively. **C** CD36 in MDSCs was not significantly changed by C18:0 or SSO treatment. Error bars indicate Standard Error of Mean; NLRP3: nucleotide-binding oligomerization domain-like receptor family pyrin domain containing 3; MDSC, myeloid-derived suppressor cell; SSO: Sulfosuccinimidyl oleate sodium; T-MDSC: total-MDSC; M-MDSC: monocytic-MDSC; G-MDSC: granulocytic-MDSC.

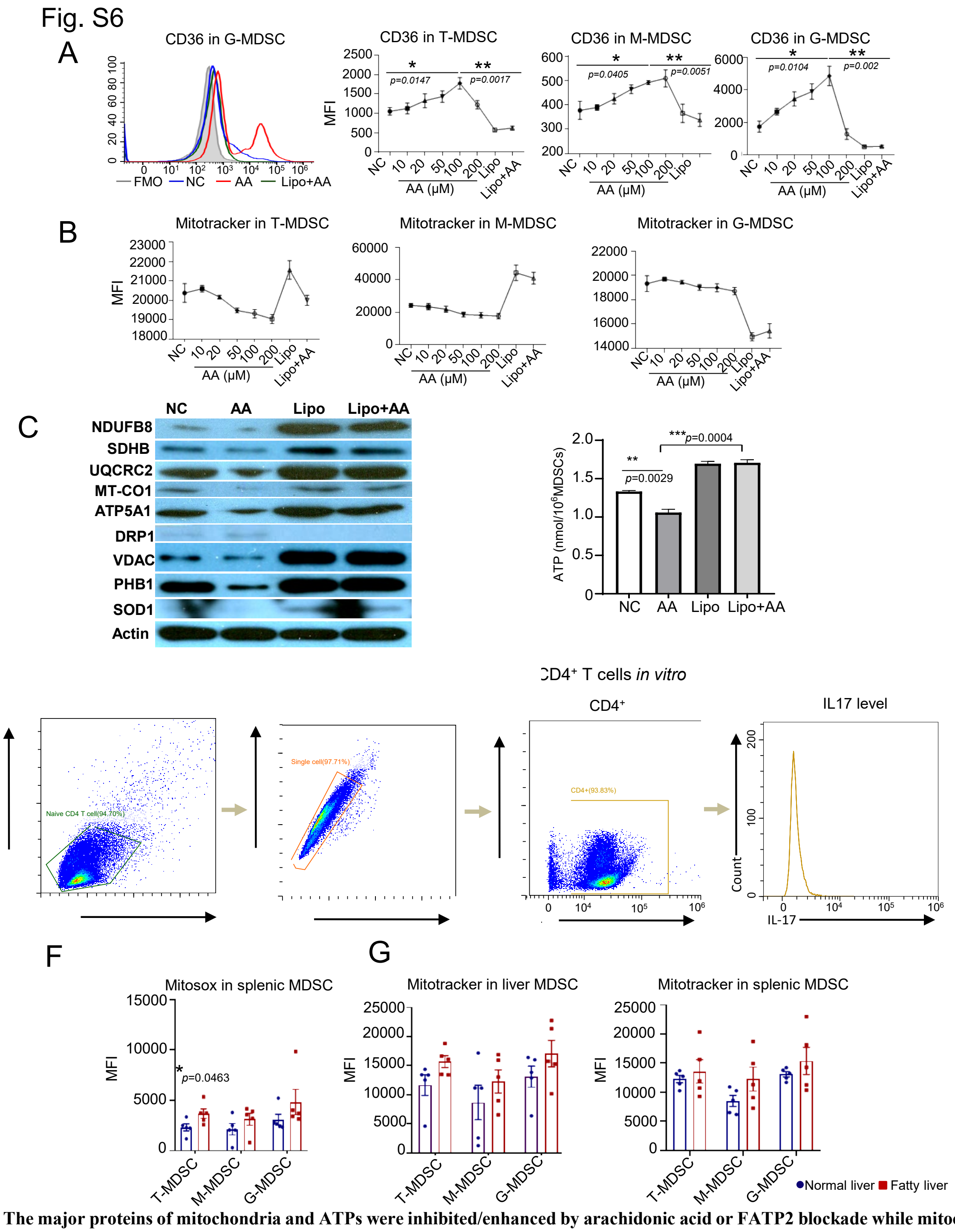

Fig. S6 The major proteins of mitochondria and ATPs were inhibited/enhanced by arachidonic acid or FATP2 blockade while mitochondrial counts were not changed obviously. A CD36 in primary MDSCs was upregulated and decreased by arachidonic acid (AA) and lipofermata (Lipo, FATP2 inhibitor), respectively. **B** The counts of mitochondria were not obviously changed by AA or lipofermata treatment. **C** The major mitochondrial protein levels, including NDUFB8 (complex I), SDHB (complex II), UQCRC2 (complex III), MT-CO1 (complex IV), ATP5A1 (complex V), VDAC, PHB1 and SOD1 in MDSCs were obviously decreased by AA but increased through the inhibition of FATP2. DRP1, the damage marker of mitochondria, was increased by AA but reduced by FATP2 inhibition. **D** The adenosine triphosphates (ATPs) produced by MDSCs were decreased by AA but enhanced by FATP2 blocking. **E** The gating strategies of IL-17 levels in CD4$^+$ T cells *in vitro*. **F** The mitosox was only increased in splenic total MDSCs. **G** The counts of mitochondria were not significantly altered post fatty liver IRI. F-G: n=5/group; Error bars indicate Standard Error of Mean; **p<0.05, **p<0.01, ***p<0.001, ****p<0.0001.* NC: negative control, the primary MDSCs without stimulation; FMO: fluorescence minus one; AA: arachidonic acid; Lipo: lipofermata; MDSC, myeloid-derived suppressor cell; T-MDSC: total-MDSC; M-MDSC: monocytic-MDSC; G-MDSC: granulocytic-MDSC; NDUFB8: NADH dehydrogenase (ubiquinone) 1 beta subcomplex subunit 8; SDHB: succinate dehydrogenase B; UQCRC2: ubiquinol-cytochrome C reductase core protein 2; MT-CO1: mitochondrially encoded cytochrome C oxidase I; ATP5A1: ATP synthase alpha-subunit; DRP1: dynamin-related protein 1; VDAC: voltage-dependent anion channel; PHB1: prohibitin 1; SOD1: superoxide dismutase 1; ATPs: adenosine triphosphates.

Fig. S7

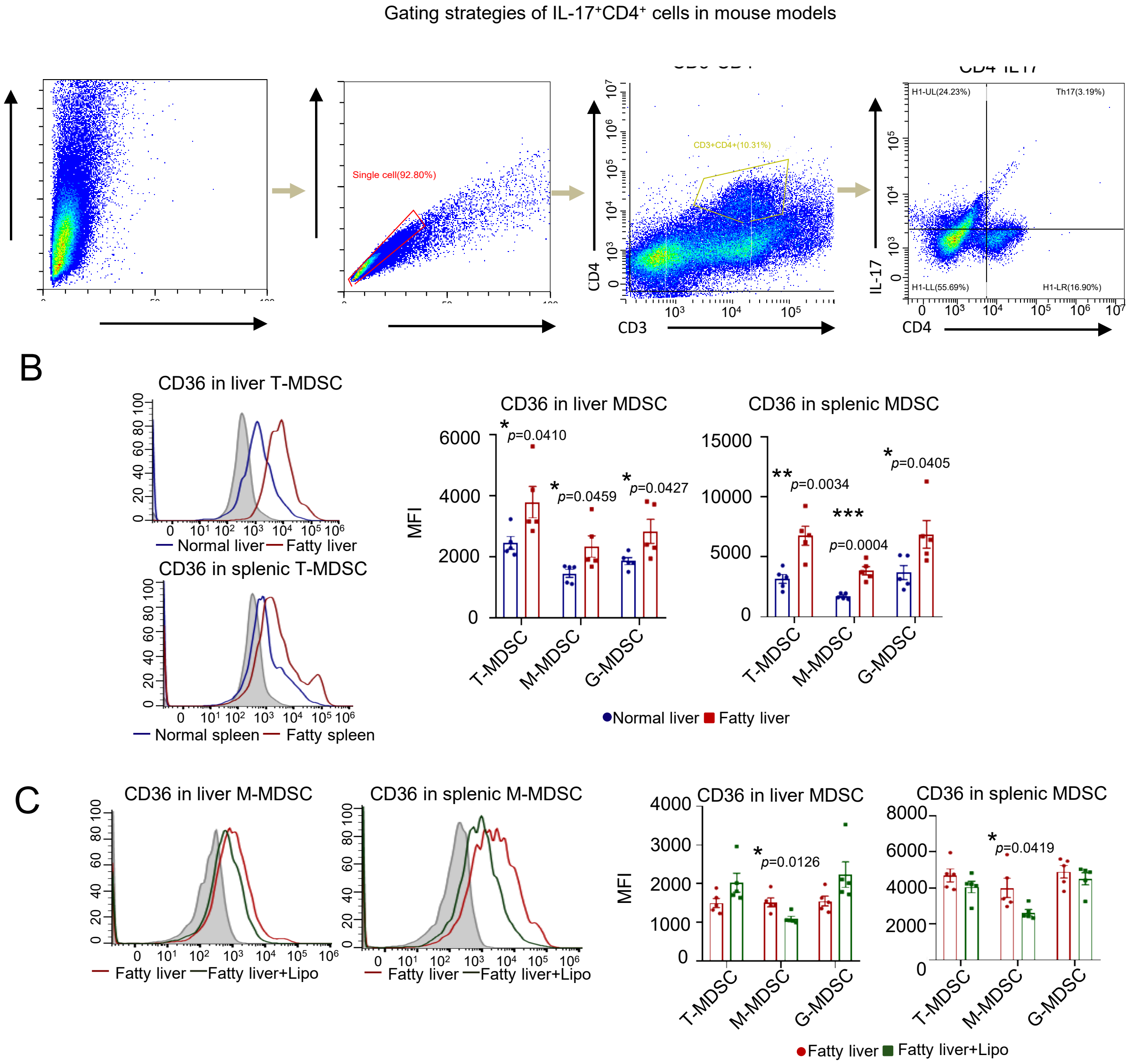

Fig. S7 CD36 in MDSCs was enhanced in fatty liver IRI and inhibited by FATP2 blockade. A The gating strategies of IL-17⁺CD4⁺ T cells in mouse model; **B** CD36 in MDSCs was increased post fatty liver IRI. **C** FATP2 blockade inhibited CD36 in monocytic MDSCs. Error bars indicate Standard Error of Mean; **p<0.05, **p<0.01, ***p<0.001, ****p<0.0001.* A-C, n=5/group; Lipo: lipofermata; MDSC, myeloid-derived suppressor cell; T-MDSC: total-MDSC; M-MDSC: monocytic-MDSC; G-MDSC: granulocytic-MDSC.

Fig. S8

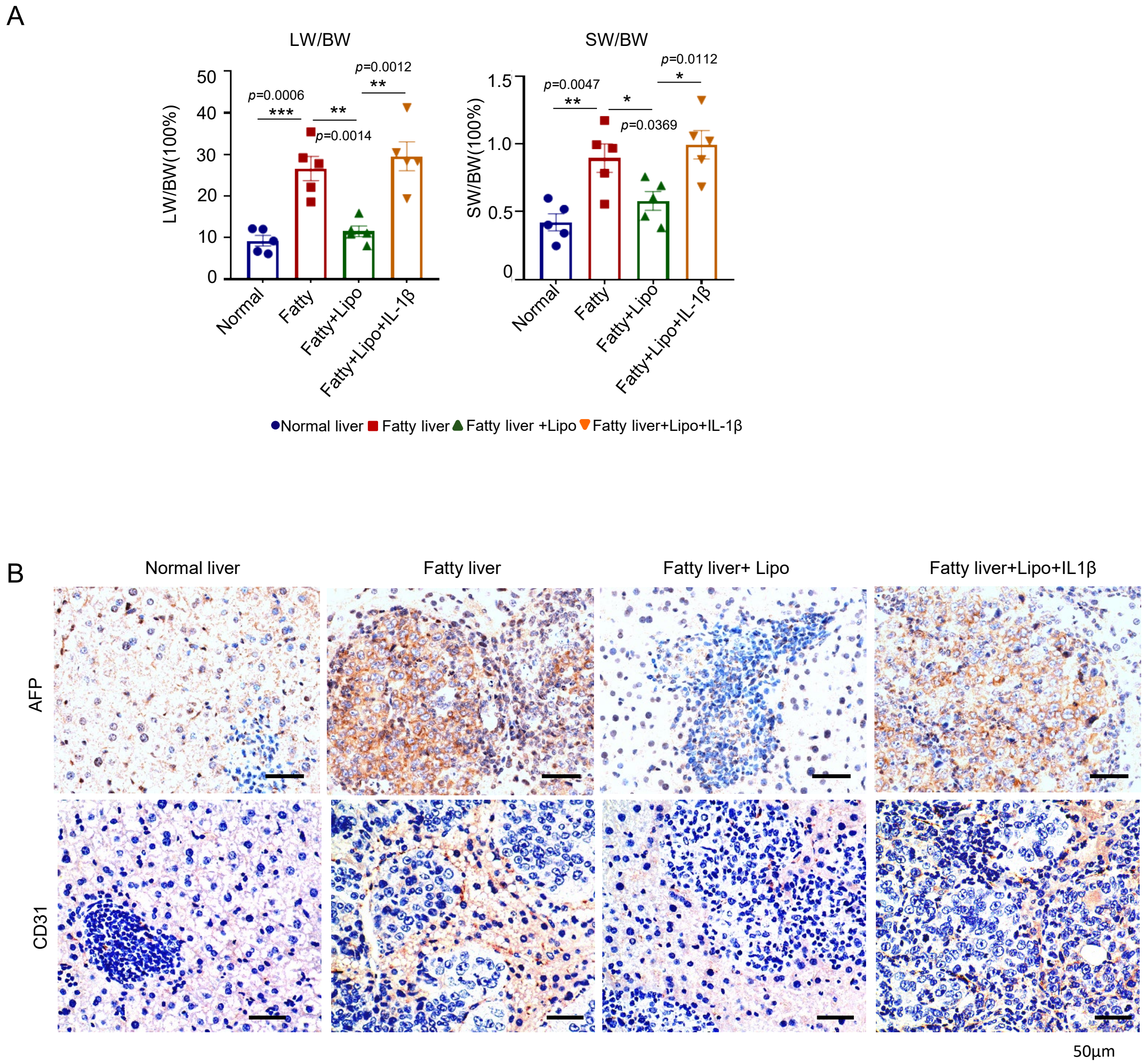

Fig. S8 The increase of tumor burden in fatty liver and reduced by FATP2 inhibition in mouse tumor recurrence model. A The increased liver weight/body weight (LW/BW) and spleen weight/body weight (SW/BW) in fatty liver group was suppressed by FATP2 inhibition and raised by IL-1β. **B** The liver infiltrated alpha fetoprotein (AFP) and CD31 positive cells were increased, decreased and restored in fatty liver, by lipofermata and IL-1β treatment, repectively. Scale bars: 50μm. A-B: n=5/group; Error bars indicate Standard Error of Mean; **p<0.05, **p<0.01, ***p<0.001, ****p<0.0001.* Lipo: lipofermata; 2; LW/BW: liver weight/body weight; SW/BW: spleen weight/body weight; AFP: alpha fetoprotein.

Fig. S9

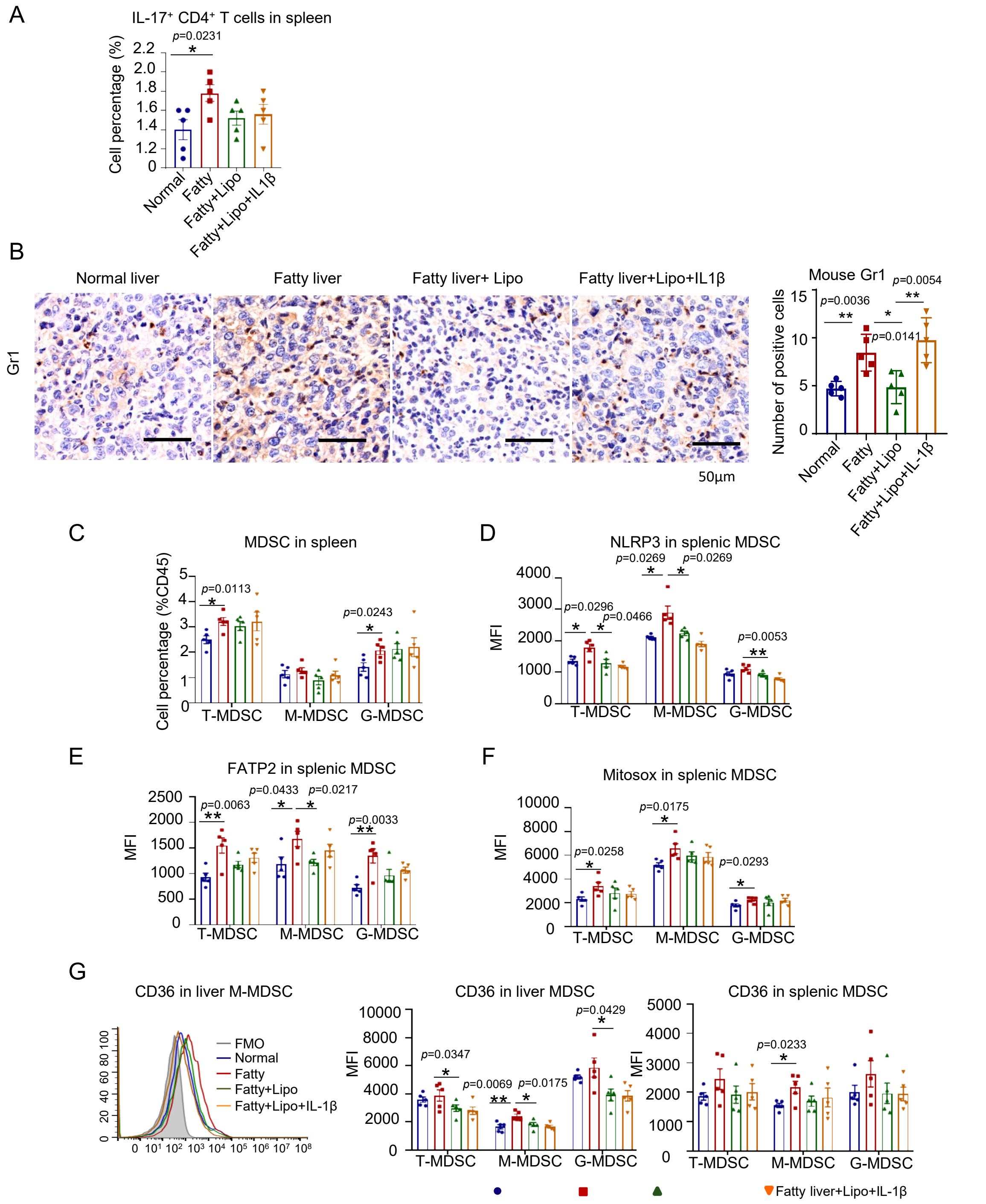

Fig. S9 The alteration of IL-17^{+}CD4^{+}T cells, MDSCs and NLRP3/FATP2/mitosox/CD36 levels in MDSCs of mouse tumor recurrence model. **A** Splenic CD4^{+}T cells produced more IL-17 in fatty liver tumor recurrence model. **B** The liver infiltrated MDSCs (Gr1^{+}) were increased, decreased and restored in fatty liver, by lipofermata and IL-1β treatment, repectively. Scale bars: 50μm. **C** Splenic T/G-MDSCs were accumulated in fatty liver tumor recurrence. **D** NLRP3 was upregualted in splenic T/M-MDSCs and diminished by lipofermata in fatty liver tumor recurrence. **E** FATP2 was raised in MDSCs while reduced in M-MDSCs by FATP2 inhibition in the spleen of fatty liver tumor recurrence model. **F** The ROS levels were enhanced in splenic MDSCs of fatty liver tumor recurrence model. **G** CD36 was increased in M-MDSCs and decreased in liver MDSCs by FATP2 blockade in fatty liver tumor recurrence model. A-G: n=5/group; Error bars indicate Standard Error of Mean; *$p<0.05$, **$p<0.01$, ***$p<0.001$, ****$p<0.0001$*. FMO: fluorescence minus one; AA: arachidonic acid; Lipo: lipofermata; MDSC, myeloid-derived suppressor cell; T-MDSC: total-MDSC; M-MDSC: monocytic-MDSC; G-MDSC: granulocytic-MDSC; NLRP3: nucleotide-binding oligomerization domain-like receptor family pyrin domain containing 3; FATP2: fatty acid transport protein 2. ROS: reactive oxygen species.

Supplementary tables

Table S1 Univariate and multivariate analyses of risk factors for tumor recurrence in HCC patients post living donor liver transplantation.

Table S2 Primers used for the amplification of genes.

	HR	95% CI	*p*
Univariate analysis			
Fatty change (⩾10% vs none)	3.147	(1.046, 9.463)	0.041*
Number of tumors	1.201	(1.103, 1.308)	<0.01**
Largest size of tumour (cm)	1.295	(1.025, 1.636)	0.03*
Vascular permeation	2.920	(1.200, 7.104)	0.018*
Macrovascular invasion	6.366	(1.406, 28.817)	0.016*
New TNM staging	3.212	(1.627, 6.340)	<0.01**
Graft weight to Recipient ESLV (⩾40 vs <40%)	1.073	(0.416, 2.771)	0.884
AFP （⩾20 vs <20 ng/ml）	1.619	(0.671, 3.907)	0.284
Cold ischemic time (⩾120 vs <120 min)	0.993	(0.400, 2.464)	0.988
Recipient warm ischemic time (⩾50 vs <50 min)	1.729	(0.667, 4.483)	0.26
Child-pugh score	0.872	(0.725, 1.050)	0.149
History of treatment for HCC before Tx (treatment vs none)	4.533	(1.506, 13.649)	0.007**
Multivariate analysis			
Fatty change (>10% vs none)	3.901	(1.039, 14.639)	0.044*
Number of tumors	1.153	(1.025, 1.297)	0.018*
Largest size of tumour (cm)	1.279	(0.903, 1.813)	0.166
Vascular permeation	0.896	(0.280, 2.875)	0.854
Macrovascular invasion	4.761	(0.666, 34.040)	0.120
New TNM staging	1.395	(0.476, 4.083)	0.544
Graft weight to Recipient ESLV cater (⩾40 vs <40%)	1.519	(0.436, 5.288)	0.512
AFP （⩾20 vs <20 ng/ml）	2.024	(0.676, 6.060)	0.208
Cold ischemic time (⩾120 vs <120 min)	0.588	(0.190, 1.823)	0.358
Recipient warm ischemic time (⩾50 vs <50 min)	2.506	(0.689, 1.057)	0.104
Child-pugh score	0.853	(0.739, 1.063)	0.146
History of treatment for HCC before Tx (treatment vs none)	2.466	(0.639, 6.519)	0.190

Primers	Forward sequence (5' to 3')	Reverse sequence (5' to 3')
Human NLRP3	CCAAAAGGAAGTGGACTGCG	TCAAACGACTCCCTGGAACG
Human IL-1β	TCAAACGACTCCCTGGAACG	GGAGCGAATGACAGAGGGTT
Rat NLRP3	CCAGGAGTTCTTTGCGGCTA	GCCTTTTTCGAACTTGCCGT
Rat IL-1β	TGACCCATGTGAGCTGAAAG	CAGGGATTTTGTCGTTGCTT
Rat NLRP1	GGACCCCATCACTATGGGAGA	AAAGACTGCTCGACTTGGGG
Rat NLRC4	TCCCAGTAGGGCTGTGTCTT	GCTGGCGGAGCTGAATATCT
Rat AIM2	AAATGCTGTTGTTGACCGGC	CTCCGTCCTGTCTGCAATGT
Rat NLRP6	CTGAGACTGGTGAGCTGTGG	ATTGCCTCACAGAGTGGACG
Mouse NLRP3	TCCCAGACACTCATGTTGCC	GTCCAGTTCAGTGAGGCTCC
Mouse IL-1β	GGCTGGACTGTTTCTAATGCC	TCTTGGCCGAGGACTAAGGA
Mouse ACSL1	ATCTGGTGGAACGAGGCAAG	TCCTTTGGGGTTGCCTGTAG
Mouse ACSL3	AGACCAGGGCTGAGTGGATGAT	CAGACGTGGGACCAAAGAGACTAT
Mouse ACSL5	AAGACGATCATCCTCATGGACC	CCTATATTCTCCGCATCATGCA
Mouse ACSL6	CGGAAGCCAGAGCAACCTTA	ATCCCAGAACCGTTGGTGAC
Mouse FATP1	GCAGCATTGCCAACATGGAC	GTGTCCTCATTGACCTTGACCAGA
Mouse FATP2	CATCGTGGTTGGGGCTACTT	GGTACCGAAGCAGTTCACCA
Mouse FATP3	CTAGAGGAAAAGGGCACCATGGCGGC	GAGCCCCTCCCTCAAGTGGAAGGATT
Mouse FATP4	TGCCCAGTCACCCAGACAAG	CATGCGGAATCCATAGTACACCAG
Mouse FATP5	AGCTATACCAGCATGTCCGC	ACCAGCCGTGACTTTACCAG
Mouse FATP6	ATCGGGTACGTTTGGCAGTT	TCCTTCGGTAGCTCCGTACA
Mouse FASN	GGAGGTGGTGATAGCCGGTAT	TGGGTAATCCATAGAGCCCAG
Mouse CD36	GATGACGTGGCAAAGAACAG	AAAGGAGGCTGCGTCTGTG
Mouse FFAR1	CCATTCTGCTCTTCTTTCTG	GGGTTTATGAAACTAGCCAC
Mouse FFAR2	CCCTGTGCACATCCTCCTGC	GCGTTCCATGCTGATGCCCG
Mouse FFAR3	TGTCCAATACTCTGCATCTGT	AGGTCCGAAATGGTCAGGTT
Mouse HCAR2	GCACAACCAGAAGTATTCCAG	CCAAATCGCCTCTCCAG
Mouse OR51E2	ACGCTGCTGTCCTCAACAAT	ACAGAAAGCCAGTCGCTTGA
Mouse SLC16A1	GTGCCATTTGCTTGCCCCT	TGGTTTTGGATGTCGTGGG
Mouse SLC16A3	ACTGACCTGACAGGCTCCAC	CTTTGGAATGACGCGGTTCC
Mouse SLC16A7	ATGCCATCTCTTATGCCC	TCTGGACGCGTGTGAAGCT
Mouse SLC22A7	CTGGTTGGGTACCTGATACG	CCTGTCTGTCTGAGCACAGT
Mouse SLC21A9	CTTCATGCTCATCCTGAG	GAGGCCTATGAATCGGTT
Mouse SLC26A3	CTGTCTCCTAGAACAGGACTGC	GCCACTGATTAGGGACTCGG
Mouse SLC21A12	TTCGGTCCCAAGTTCTTCG	CACTTCCCTGCGGTAGCAT
Mouse SLC5A8	GCCCCTTGAAACCTATGGCT	CAGTGGAGTCCTTTCCGCAT
Mouse FABP1	GGAAGGACATCAAGGGGGTG	GTCATGGTCTCCAGTTCGCA
Mouse FABP2	AGTTGAGGCCAAGCGATTCT	GCCTGGCATTAGCATGATGG
Mouse FABP3	TGCTGCCTCATGGTTTTCCC	GACCTTGGAGCACCCTTTGG
Mouse FABP4	TGAAATCACCGCAGACGACA	ACACATTCCACCACCAGCTT
Mouse FABP5	GCTGCTTTTGTGCTCTCCCT	GCAGACCGTCTAGCTCTTTCAT
Mouse FABP6	GTGGCAGAGTTCCCCAACTA	TGCTTACGCGCTCATAGGTC
Mouse MSR1	AGGGAAGTGGATAAATCAGTGCT	TGCAGTCAGCATCCTCTTGT
Mouse MARCO	GGGAACATCTGGCTGGACAA	GCTCCCAAGTCAGGAGCATT
Mouse Scarb1	ATAAAGCCTCTGGCCACCTG	ACCTGAAGGAGACGGAGACA
Mouse CD68	TGTTCAGCTCCAAGCCCAAA	GTACCGTCACAACCTCCCTG
Mouse LRP1	GGCGGTGTGACAACGACAAT	CACGTCCAGTGCTCGGGG
Mouse LRP8	TGTCCACACACGGATTGGTT	TTCACAGACAGCAAGTGGCA
Mouse VLDLR	TGACTGTCGGTGTGTGTTGT	CTCAGAGCTGAGTGCCCTTG
Mouse LDLR	GCCAGGAAAGTGACTCGTGA	AAGGGAGAATGGCGACTTGG

Table S3 Antibodies used for the staining.

Name	Citation (PMID)	Supplier	Cat no.	Clone no.
Anti-CD33	8822961	BD Biosciences	555450	WM53
Anti-CD13	2665173	BD Biosciences	557454	WM15
Anti-CD34	26395069	Militenyi Biotech	130-081-001	AC136
Anti-His48	2046327	BD Pharmingen	554907	HIS48
Anti-CD80	9237108	BD Biosciences	555012	3H5
Anti-CD11b/c	-	Invitrogen	MA5-17507	OX-42
Anti-CD11b	6184305	BD Biosciences	557397	M1/70
Anti-CD11b	29276143	Biolegend	101226	M1/70
Anti-Gr1	-	Stem Cell	60028AZ	RB6-8C5
Anti-Gr1	29262351	Biolegend	108406	RB6-8C5
Anti-Ly6G	30955887	Biolegend	127622	1A8
Anti-Ly6C	11754008	BD Biosciences	563011	AL-21
Anti-CD3	29548673	Biolegend	100330	145-2C11
Anti-CD19	29101162	Biolegend	115530	6D5
Anti-CD45	22547694	Biolegend	103138	30-F11
Anti-CD36	11560944	BD Biosciences	562744	CRF D-2712
Anti-NLRP3	31558756	R&D	IC7578A	768319
Anti-SLC27A2	-	Invitrogen	PA5-102343	Polyclonal
Anti-CD4	30610104	Biolegend	116005	RM4-4
Anti-IL-17A	18606690	Biolegend	506909	TC11-18H10.1
Anti-CD44	30194420	Biolegend	103011	IM7
Anti-CD62L	33440157	Invitrogen	63-0621-80	MEL-14
Anti-NLRP3	19364881	Santa Cruz Biotech	sc-66846	Polyclonal
Anti-NLRP3	34651582	Cell signaling	15101S	D4D8T
Anti-ASC	27882934	Sigma-Aldrich	SAB4501315	Polyclonal
Anti-CD36	-	Cell signaling	74002	Polyclonal
Anti-C-caspase-1	33621216	Cell signaling	89332	E2G2I
Anti-CD33	23708142	Leica	NCL-L-CD33	PWS44
Anti-CD11b/c	1672643	BD Pharmingen	550299	OX-42
Anti-CD15	8236092	BD Pharmingen	559045	MMA
Anti-Lox1	32235836	Abcam	ab60178	Polyclonal
Anti-His48	32109414	Abcam	ab33760	HIS48
Anti-VDAC	35505004	Cell signaling	4661p	D73D12
Anti-SDHB	33431792	Abcam	ab14714	21A11AE7
Anti-PHB1	32778760	Cell signaling	2426	Polyclonal
Anti-SOD1	32402267	Cell signaling	4266	71G8
Anti-DRP1	18838687	Cell signaling	14647	4E11B11
Anti-NDUFB8	34697471	Invitrogen	459210	20E9DH10C12
Anti-UQCRC2	-	Invitrogen	PA5-53939	Polyclonal
Anti-MT-CO1	24360282	Cell signaling	62101	Polyclonal
Anti-ATP5A1	35900274	Cell signaling	18023	Polyclonal
Anti-AFP	34958137	Abcam	ab46799	Polyclonal
Anti-CD31	25079331	Invitrogen	PA5-16301	Polyclonal

Supplementary figure legends

Fig. S1 The tumor recurrent rate was higher in HCC patients underwent liver transplantation using steatotic donor accompanied with the increase of MDSCs and NLRP3/FATP2 levels. A The tumor recurrence free survival was relatively poor in HCC patients after liver transplantation using steatotic donor (n=88). **B** The gating strategies of human MDSCs ($CD33^{+}CD13^{+}CD34^{+}$) by flow cytometry (n=45). **C** The number of MDSCs ($CD33^{+}$) were significantly accumulated in steatotic grafts by immunohistochemistry (n=23). **D** More MDSCs were infiltrated in fatty grafts by co-staining of CD15 & LOX1 (n=23). Scale bars: 20μm. **E** The increased numbers of NLRP3 and FATP2 positive cells in fatty grafts by immunohistochemistry (n=23). **F** More $CD33^{+}NLRP3^{+}$ and $CD33^{+}FATP2^{+}$ cells were infiltrated in fatty grafts post liver transplantation by co-staining (n=23). Error bars indicate Standard Error of Mean; *$p<0.05$, **$p<0.01$, ***$p<0.001$, ****$p<0.0001$. MDSC, myeloid-derived suppressor cell; NLRP3: nucleotide-binding oligomerization domain-like receptor family pyrin domain containing 3; FATP2: fatty acid transport protein 2.

Fig. S2 The levels of NLRP3 and FATP2 were higher with accumulation of MDSCs in fatty liver grafts of rat model. A The gating strategies of rat MDSCs ($CD11b/c^{+}CD80^{+}His48^{+}$) by flow cytometry. **B** The number of $CD11b/c^{+}$ (MDSC marker) cells were obviously increased in rat fatty liver grafts by immunohistochemistry. **C** Accumulated MDSCs in fatty grafts by co-staining of CD11b/c & His48. Scale bars: 20μm. **D** NLRP3 was upreglated in fatty grafts through the inflammsome (NLRP1, NLRP3, NLRC4, AIM2, NLRP6) mRNA screen of rat liver transplantation model. **E** NLRP3 and FATP2 positive cells were more in fatty liver grafts by immunohistochemistry. **F** The immunofluorescent co-staining showed increased

$CD11b/c^+NLRP3^+$ and $CD11b/c^+FATP2^+$ cells in fatty grafts post liver transplantation. A-F: n=4/group; Error bars indicate Standard Error of Mean; $^*p<0.05$, $^{**}p<0.01$, $^{***}p<0.001$, $^{****}p<0.0001$. MDSC, myeloid-derived suppressor cell; NLRP: nucleotide-binding oligomerization domain-like receptor family pyrin domain containing; FATP2: fatty acid transport protein 2; NLRC4: NLR Family CARD Domain Containing 4; AIM2: Absent In Melanoma 2.

Fig. S3 The severer inflammatory injury and poorer liver function with enhanced NLRP3 and IL-1β expressions in mice post fatty liver ischemia/reperfusion injury (IRI). A The inflammatory markers, included MCP-1, IL-6, CXCL1, IFN-γ, IL-1β and TLR4, were significantly upregulated post fatty liver IRI. **B** The liver function was poorer with increased alanine transaminase (ALT) and aspartate aminotransferase (AST) in fatty liver after IRI. **C** The mRNA levels of NLRP3 and IL-1β were increased in fatty liver post IRI in mice**.** A-C: n=5/group; Error bars indicate Standard Error of Mean; $^*p<0.05$, $^{**}p<0.01$. NLRP3: nucleotide-binding oligomerization domain-like receptor family pyrin domain containing 3; IRI, ischemia/reperfusion injury; MCP-1: Monocyte Chemoattractant Protein 1; IL-6: Interleukin-6; CXCL1: C-X-C Motif chemokine ligand 1; IFN-γ: Interferon-γ; TNF-α: tumor necrosis factor α; TLR4: Toll Like Receptor 4; ALT: alanine transaminase; AST, aspartate aminotransferase.

Fig. S4 No obvious change of Dil-LDL and free fatty acid in MDSCs of mice post fatty liver IRI. A The gating strategies of Total MDSC (T-MDSC, $CD11b^+Gr1^+$), Granulocytic MDSC (G-MDSC, $CD11b^+$ $Ly6G^+Ly6C^{low}$) and Monocytic MDSC (M-MDSC, $CD11b^+$ $Ly6G^-Ly6C^{high}$) in mice. **B** Dil-LDL in MDSCs was not significantly changed in mice after fatty liver IRI. **C** Free fatty acids were not altered obviously post fatty liver IRI. A-C: n=5/group; Error bars indicate Standard Error of Mean; $^*p<0.05$, $^{**}p<0.01$. IRI, ischemia/reperfusion injury; MDSC, myeloid-

derived suppressor cell; LDL: low density lipoprotein; T-MDSC: total-MDSC; M-MDSC: monocytic-MDSC; G-MDSC: granulocytic-MDSC.

Fig. S5 NLRP3 in primary MDSCs was not changed by C18:0 stimulation. **A** FL C16 in primary MDSCs was not obviously altered by C18:0 or SSO (CD36 inhibitor) treatment. **B** C18:0 or SSO could not activate or inhibit NLRP3 in MDSCs, respectively. **C** CD36 in MDSCs was not significantly changed by C18:0 or SSO treatment. Error bars indicate Standard Error of Mean; NLRP3: nucleotide-binding oligomerization domain-like receptor family pyrin domain containing 3; MDSC, myeloid-derived suppressor cell; SSO: Sulfosuccinimidyl oleate sodium; T-MDSC: total-MDSC; M-MDSC: monocytic-MDSC; G-MDSC: granulocytic-MDSC.

Fig. S6 The major proteins of mitochondria and ATPs were inhibited/enhanced by arachidonic acid or FATP2 blockade while mitochondrial counts were not changed obviously. **A** CD36 in primary MDSCs was upregulated and decreased by arachidonic acid (AA) and lipofermata (Lipo, FATP2 inhibitor), respectively. **B** The counts of mitochondria were not obviously changed by AA or lipofermata treatment. **C** The major mitochondrial protein levels, including NDUFB8 (complex I), SDHB (complex II), UQCRC2 (complex III), MT-CO1 (complex IV), ATP5A1 (complex V), VDAC, PHB1 and SOD1 in MDSCs were obviously decreased by AA but increased through the inhibition of FATP2. DRP1, the damage marker of mitochondria, was increased by AA but reduced by FATP2 inhibition. **D** The adenosine triphosphates (ATPs) produced by MDSCs were decreased by AA but enhanced by FATP2 blocking. **E** The gating strategies of IL-17 levels in $CD4^+$ T cells *in vitro*. **F** The mitosox was only increased in splenic total MDSCs. **G** The counts of mitochondria were not significantly altered post fatty liver IRI. F-G: n=5/group; Error bars indicate Standard Error of Mean; $^{*}p<0.05$, $^{**}p<0.01$, $^{***}p<0.001$,

$^{****}p<0.0001$. NC: negative control, the primary MDSCs without stimulation; FMO: fluorescence minus one; AA: arachidonic acid; Lipo: lipofermata; MDSC, myeloid-derived suppressor cell; T-MDSC: total-MDSC; M-MDSC: monocytic-MDSC; G-MDSC: granulocytic-MDSC; NDUFB8: NADH dehydrogenase (ubiquinone) 1 beta subcomplex subunit 8; SDHB: succinate dehydrogenase B; UQCRC2: ubiquinol-cytochrome C reductase core protein 2; MT-CO1: mitochondrially encoded cytochrome C oxidase I; ATP5A1: ATP synthase alpha-subunit; DRP1: dynamin-related protein 1; VDAC: voltage-dependent anion channel; PHB1: prohibitin 1; SOD1: superoxide dismutase 1; ATPs: adenosine triphosphates.

Fig. S7 CD36 in MDSCs was enhanced in fatty liver IRI and inhibited by FATP2 blockade. **A** The gating strategies of IL-17^{+}CD4^{+} T cells in mouse model; **B** CD36 in MDSCs was increased post fatty liver IRI. **C** FATP2 blockade inhibited CD36 in monocytic MDSCs. Error bars indicate Standard Error of Mean; $^{*}p<0.05$, $^{**}p<0.01$, $^{***}p<0.001$, $^{****}p<0.0001$. A-C, n=5/group; Lipo: lipofermata; MDSC, myeloid-derived suppressor cell; T-MDSC: total-MDSC; M-MDSC: monocytic-MDSC; G-MDSC: granulocytic-MDSC.

Fig. S8 The increase of tumor burden in fatty liver and reduced by FATP2 inhibition in mouse tumor recurrence model. **A** The increased liver weight/body weight (LW/BW) and spleen weight/body weight (SW/BW) in fatty liver group was suppressed by FATP2 inhibition and raised by IL-1β. **B** The liver infiltrated alpha fetoprotein (AFP) and CD31 positive cells were increased, decreased and restored in fatty liver, by lipofermata and IL-1β treatment, repectively. Scale bars: 50μm. A-B: n=5/group; Error bars indicate Standard Error of Mean; $^{*}p<0.05$, $^{**}p<0.01$, $^{***}p<0.001$, $^{****}p<0.0001$. FATP2: fatty acid transport protein 2; LW/BW: liver weight/body weight; SW/BW: spleen weight/body weight; AFP: alpha fetoprotein.

Fig. S9 The alteration of IL-17$^+$CD4$^+$T cells, MDSCs and NLRP3/FATP2/mitosox/CD36 levels in MDSCs of mouse tumor recurrence model. **A** Splenic CD4$^+$ T cells produced more IL-17 in fatty liver tumor recurrence model. **B** The liver infiltrated MDSCs (Gr1$^+$) were increased, decreased and restored in fatty liver, by lipofermata and IL-1β treatment, repectively. Scale bars: 50μm. **C** Splenic T/G-MDSCs were accumulated in fatty liver tumor recurrence. **D** NLRP3 was upregualted in splenic T/M-MDSCs and diminished by lipofermata in fatty liver tumor recurrence. **E** FATP2 was raised in MDSCs while reduced in M-MDSCs by FATP2 inhibition in the spleen of fatty liver tumor recurrence model. **F** The ROS levels were enhanced in splenic MDSCs of fatty liver tumor recurrence model. **G** CD36 was increased in M-MDSCs and decreased in liver MDSCs by FATP2 blockade in fatty liver tumor recurrence model. A-G: n=5/group; Error bars indicate Standard Error of Mean; *$p<0.05$, **$p<0.01$, ***$p<0.001$, ****$p<0.0001$. FMO: fluorescence minus one; AA: arachidonic acid; Lipo: lipofermata; MDSC, myeloid-derived suppressor cell; T-MDSC: total-MDSC; M-MDSC: monocytic-MDSC; G-MDSC: granulocytic-MDSC; NLRP3: nucleotide-binding oligomerization domain-like receptor family pyrin domain containing 3; FATP2: fatty acid transport protein 2; ROS: reactive oxygen species.

Supplementary materials and methods

Quantitative Real-Time Polymerase Chain Reaction (qRT-PCR)

Total RNA was extracted from human liver specimens, rat/mouse liver tissues and MDSCs isolated from mouse liver/spleen using Trizol Reagent (Invitrogen, CA, USA). Complementary DNA was synthesized from 1μg or 500ng total RNA using High Capacity cDNA Reverse Transcription Kits (Applied Biosystems, CA, USA). qRT-PCR was performed using Viia7 Fast Real-time PCR system (Life Technologies, CA, USA) with primers listed in **Table S2** and Fast Start SYBR Green Master Mix (Promega, WI, USA).

Flow Cytometry Analysis

Flow cytometry was carried out with a modified version of the previous protocol(1). Human MDSCs were detected by CD33, CD13 (BD Pharmingen, CA, USA) and CD34 (Militenyi Biotech, Bergisch Gladbach, Germany). Rat MDSC population was detected by incubation with His48, CD80 (BD Pharmingen), CD11b/c (Invitrogen) antibodies. Mouse total MDSCs was labeled by Gr1 (Biolegend, CA, USA/Stem cell, Stemcell Technologies, BC, Canada), CD11b (BD Pharmingen/ Biolegend). The monocytic/granulocytic MDSCs of mouse was checked by CD11b (BD Pharmingen/Biolegend), Ly6G (Biolegend) and Ly6C (BD Horizon). Moreover, mouse CD45, CD3 and CD19 (Biolegend) were stained to gate the cells and avoid the lymphocytes. NLRP3 (R&D, MN, USA), FATP2 (Invitrogen), CD36 (BD Pharmingen), IL17 (Biolegend) antibodies were used to investigate their levels. BODIPY FL C16, 493/503, low density lipoprotein from human plasma-DiI complex (Dil LDL), MitoTracker green FM, MitoSox red mitochondrial superoxide indicator (Invitrogen) and free fatty acid (Abcam, Cambridge, MA, USA) was stained to investigate the lipid level and mitochondrial alteration. The purity of primary MDSCs and naïve

$CD4^+$ T cells were determined by $Gr1^+CD11b^+$ (Biolegend) and $CD4^+CD44^+CD62L$ (Biolegend/Invitrogen), respectively. Before detecting the IL-17 levels in $CD4^+$ T cells, the single cell suspension from liver and spleen of mouse model was stimulated with cell activation cocktail (Brefeldin A, Biolegend) for at least 4 hours at 37 °C. The cells were implemented to permeabilization for 30 mins. After that, IL-17 was stained to explore the levels by flow cytometry. Labeled cells were analyzed by BD FACS Calibur (BD Biosciences, CA, USA), Cytoflex S (Beckman Coulter, CA, USA), FlowJo (Treestar, San Carlos, CA, USA).

Immunostaining

The detailed protocols of immunohistochemistry and immunofluorescent staining was performed using the previous methods with modification(1-3). Antibodies against human CD33 (Leica, Milton Keynes, UK), human CD15 (BD Pharmingen), LOX1 (Abcam), rat CD11b/c (BD Pharmingen), rat His 48 (Abcam), human/rat/mouse NLRP3 (Santa Cruz, CA, USA) and FATP2 (Invitrogen) were used. Mitochondria was detected by MitoTracker red CMXRos/green FM, MitoSox red mitochondrial superoxide indicator (Invitrogen) and Dihydrorhodamine 123 (Sigma-Aldrich, Darmstadt, Germany). The Hamamatsu imaging system (Hamamatsu Photonics, Shizuoka, Japan) and Carl Zeiss LSM 780 imaging System (Carl Zeiss, Jena, Germany) was used to acquire the images and count the positive cells of five randomly chosen views in each slide (×400) for statisctics.

RNA sequencing

Total RNA was extracted from rat liver graft at 6 hours after transplantation using normal or fatty donor (n=3). RNA high throughout sequencing was performed using Illumina PE150. RNA-seq data have been submmited and are available through the NCBI's Gene Expression Omnibus (GEO

GSE204919). The results were analyzed by Kyoto Encyclopedia of Genes and Genomes and Gene Set Enrichment Analysis.

Gas chromatography-mass spectrometry analysis of fatty acids

The tissues of rat normal/fatty liver and grafts at 6 hours after liver transplantation using normal/fatty donor were prepared for gas chromatography-mass spectrometry analysis (n=4). The whole procedure was finished by Center for Proteomics and Metabolomics core, Core PanorOmic Sciences, LKS faculty of Medicine, The University of Hong Kong. The detailed protocols could be acquired from the core. Total 36 types of fatty acids (medium to long chain) were detected including C4:0, C8:0, C10:0, C11:0, C12:0, C13:0, C14:0, C14:1, C15:0, C15:1, C16:0, C16:1, C17:0, C17:1, C18:0, C18:1, trans, C18:1, cis, C18:2, trans, C18:2, cis, C18:3 n6, C18:3 n3, C20:0, C20:1 n9, C20:2, C21:0, C20:3 n6, C20:4 n6, C20:3 n3, C20:5, C22:0, C22:1, C22:2, C23:0, C24:0, C22:6, C24:1, C6:0, C26:0. The changed fatty acids were analyzed.

Western blot

Western blot was carried out according to the protocol described previously(3). Anti-mouse FATP2 (Invitrogen), ASC (Sigma-Aldrich), NLRP3, cleaved-caspase-1, CD36, MT-CO1, ATP5A1, DRP1, VDAC, PHB1, SOD1 (Cell Signaling Technology, MA, USA), NDUFB8, UQCRC2 (Invitrogen), SDHB (Abcam) antibodies were used.

ATP quantification assay

The evaluation of total ATPs in MDSCs was performed according to the manufacturer's instructions of ATP colorimetric assay (Abcam). Briefly, the isolated MDSCs with arachidonic acid/lipofermata stimulation was homogenized in 100 µl ATP assay buffer and the homogenate was centrifuged (13,000g, 5min, 4°C) to harvest supernatant. Add cold 4M perchloric acid to a

final concentration 1M in the supernatant, mix well and centrifuge (13,000g, 5min, 4℃). One-third volume of 2M KOH was added to the supernatant and the mixture pH was adjusted to 6.5-8.0. The mixture was centrifuged (13,000g, 15min, 4℃) again to obtain supernatant. Add 50μL reaction mixture or background reaction mixture with equal volume of the last supernatant to each well and incubate at room temperature for 30min. The result was read from the absorbance at OD 570nm.

Supplementary references

1. Ling C-C, Ng KT, Shao Y, Geng W, Xiao J-W, Liu H*, et al.* Post-transplant endothelial progenitor cell mobilization via CXCL10/CXCR3 signaling promotes liver tumor growth. J Hepatol **2014**;60(1):103-9.
2. Yeung OW, Lo C-M, Ling C-C, Qi X, Geng W, Li C-X*, et al.* Alternatively activated (M2) macrophages promote tumour growth and invasiveness in hepatocellular carcinoma. J Hepatol **2015**;62(3):607-16.
3. Liu H, Lo CM, Yeung OWH, Li CX, Liu XB, Qi X*, et al.* NLRP3 inflammasome induced liver graft injury through activation of telomere-independent RAP1/KC axis. The Journal of pathology **2017**;242(3):284-96.
