# Supplementary material for: Arachidonic acid activates NLRP3 inflammasome in MDSCs via FATP2 to promote post-transplant tumour recurrence in steatotic liver grafts
Source: JHEP Rep. 2023 Aug 22;5(12):100895. doi: 10.1016/j.jhepr.2023.100895 (PMC10616418; doi:10.1016/j.jhepr.2023.100895)
Supplement: Multimedia component 2 [file mmc2.docx]

**Journal of Hepatology**

**CTAT methods**

Tables for a “Complete, Transparent, Accurate and Timely account” (CTAT) are now mandatory for all revised submissions. The aim is to enhance the reproducibility of methods.

- Only include the parts relevant to your study
- Refer to the CTAT in the main text as ‘Supplementary CTAT Table’
- Do not add subheadings
- Add as many rows as needed to include all information
- Only include one item per row

**If the CTAT form is not relevant to your study, please outline the reasons why:**

|  |
| --- |

- 1. **Antibodies**

| **Name** | **Citation** | **Supplier** | **Cat no.** | **Clone no.** |
| --- | --- | --- | --- | --- |
| Anti-CD33  Anti-CD13  Anti-CD34  Anti-His48  Anti-CD80  Anti-CD11b/c  Anti-CD11b  Anti-CD11b  Anti-Gr1  Anti-Gr1  Anti-Ly6G  Anti-Ly6C  Anti-CD3  Anti-CD19  Anti-CD45  Anti-CD36  Anti-NLRP3  Anti-SLC27A2  Anti-CD4  Anti-IL-17A  Anti-CD44  Anti-CD62L  Anti-NLRP3  Anti-NLRP3  Anti-ASC  Anti-CD36  Anti-C-caspase-1  Anti-CD33  Anti-CD11b/c  Anti-CD15  Anti-Lox1  Anti-His48  Anti-VDAC  Anti-SDHB  Anti-PHB1  Anti-SOD1  Anti-DRP1  Anti-NDUFB8  Anti-UQCRC2  Anti-MT-CO1  Anti-ATP5A1  Anti-AFP  Anti-CD31 | 8822961  2665173  26395069  2046327  9237108  -  6184305  29276143  -  29262351  30955887  11754008  29548673  29101162  22547694  11560944  31558756  -  30610104  18606690  30194420  33440157  19364881  34651582  27882934  -  33621216  23708142  1672643  8236092  32235836  32109414  35505004  33431792  32778760  32402267  18838687  34697471  -  24360282  35900274  34958137  25079331 | BD Biosciences  BD Biosciences  Militenyi Biotech  BD Pharmingen  BD Biosciences  Invitrogen  BD Biosciences  Biolegend  Stem Cell  Biolegend  Biolegend  BD Biosciences  Biolegend  Biolegend  Biolegend  BD Biosciences  R&D  Invitrogen  Biolegend  Biolegend  Biolegend  Invitrogen  Santa Cruz Biotech  Cell signaling  Sigma-Aldrich  Cell signaling  Cell signaling  Leica  BD Pharmingen  BD Pharmingen  Abcam  Abcam  Cell signaling  Abcam  Cell signaling  Cell signaling  Cell signaling  Invitrogen  Invitrogen  Cell signaling  Cell signaling  Abcam  Invitrogen | 555450  557454  130-081-001  554907  555012  MA5-17507  557397  101226  60028AZ  108406  127622  563011  100330  115530  103138  562744  IC7578A  PA5-102343  116005  506909  103011  63-0621-80  sc-66846  15101S  SAB4501315  74002  89332  NCL-L-CD33  550299  559045  ab60178  ab33760  4661p  ab14714  2426  4266  14647  459210  PA5-53939  62101  18023  ab46799  PA5-16301 | WM53  WM15  AC136  HIS48  3H5  OX-42  M1/70  M1/70  RB6-8C5  RB6-8C5  1A8  AL-21  145-2C11  6D5  30-F11  CRF D-2712  768319  Polyclonal  RM4-4  TC11-18H10.1  IM7  MEL-14  Polyclonal  D4D8T  Polyclonal  Polyclonal  E2G2I  PWS44  OX-42  MMA  Polyclonal  HIS48  D73D12  21A11AE7  Polyclonal  71G8  4E11B11  20E9DH10C12  Polyclonal  Polyclonal  Polyclonal  Polyclonal  Polyclonal |

- 1. **Cell lines**

| **Name** | **Citation** | **Supplier** | **Cat no.** | **Passage no.** | **Authentication test method** |
| --- | --- | --- | --- | --- | --- |
| Hepa1-6 | 27245433 | ATCC | CRL-1601 | 6 | STR |

- 1. **Organisms**

| **Name** | **Citation** | **Supplier** | **Strain** | **Sex** | **Age** | **Overall n number** |
| --- | --- | --- | --- | --- | --- | --- |
| Rats | 23994383 | Laboratory animal unit, HKU | Sprague Dawley (SD) | M | 6-8 weeks | 30 |
| Mouse | 27245433 | Laboratory animal unit, HKU | C57 BL/6 | M | 6-8 weeks | 70 |

- 1. **Sequence based reagents**

| **Name** | **Sequence** |  | **Supplier** |
| --- | --- | --- | --- |
| Human NLRP3 | CCAAAAGGAAGTGGACTGCG | TCAAACGACTCCCTGGAACG |  |
| Human IL-1β | TCAAACGACTCCCTGGAACG | GGAGCGAATGACAGAGGGTT |  |
| Rat NLRP3 | CCAGGAGTTCTTTGCGGCTA | GCCTTTTTCGAACTTGCCGT |  |
| Rat IL-1β | TGACCCATGTGAGCTGAAAG | CAGGGATTTTGTCGTTGCTT |  |
| Rat NLRP1  Rat NLRC4  Rat AIM2  Rat NLRP6  Mouse NLRP3  Mouse IL-1β  Mouse ACSL1  Mouse ACSL3  Mouse ACSL5  Mouse ACSL6  Mouse FATP1  Mouse FATP2  Mouse FATP3  Mouse FATP4  Mouse FATP5  Mouse FATP6  Mouse FASN  Mouse CD36  Mouse FFAR1  Mouse FFAR2  Mouse FFAR3  Mouse HCAR2  Mouse OR51E2  Mouse SLC16A1  Mouse SLC16A3  Mouse SLC16A7  Mouse SLC22A7  Mouse SLC21A9  Mouse SLC26A3  Mouse SLC21A12  Mouse SLC5A8  Mouse FABP1  Mouse FABP2  Mouse FABP3  Mouse FABP4  Mouse FABP5  Mouse FABP6  Mouse MSR1  Mouse MARCO  Mouse Scarb1  Mouse CD68  Mouse LRP1  Mouse LRP8  Mouse VLDLR  Mouse LDLR | GGACCCCATCACTATGGGAGA  TCCCAGTAGGGCTGTGTCTT  AAATGCTGTTGTTGACCGGC  CTGAGACTGGTGAGCTGTGG  TCCCAGACACTCATGTTGCC  GGCTGGACTGTTTCTAATGCC  ATCTGGTGGAACGAGGCAAG  AGACCAGGGCTGAGTGGATGAT  AAGACGATCATCCTCATGGACC  CGGAAGCCAGAGCAACCTTA  GCAGCATTGCCAACATGGAC  CATCGTGGTTGGGGCTACTT  CTAGAGGAAAAGGGCACCATGGCGGC  TGCCCAGTCACCCAGACAAG  AGCTATACCAGCATGTCCGC  ATCGGGTACGTTTGGCAGTT  GGAGGTGGTGATAGCCGGTAT  GATGACGTGGCAAAGAACAG  CCATTCTGCTCTTCTTTCTG  CCCTGTGCACATCCTCCTGC  TGTCCAATACTCTGCATCTGT  GCACAACCAGAAGTATTCCAG  ACGCTGCTGTCCTCAACAAT  GTGCCATTTGCTTGCCCCT  ACTGACCTGACAGGCTCCAC  ATGCCATCTCTTATGCCC  CTGGTTGGGTACCTGATACG  CTTCATGCTCATCCTGAG  CTGTCTCCTAGAACAGGACTGC  TTCGGTCCCAAGTTCTTCG  GCCCCTTGAAACCTATGGCT  GGAAGGACATCAAGGGGGTG  AGTTGAGGCCAAGCGATTCT  TGCTGCCTCATGGTTTTCCC  TGAAATCACCGCAGACGACA  GCTGCTTTTGTGCTCTCCCT  GTGGCAGAGTTCCCCAACTA  AGGGAAGTGGATAAATCAGTGCT  GGGAACATCTGGCTGGACAA  ATAAAGCCTCTGGCCACCTG  TGTTCAGCTCCAAGCCCAAA  GGCGGTGTGACAACGACAAT  TGTCCACACACGGATTGGTT  TGACTGTCGGTGTGTGTTGT  GCCAGGAAAGTGACTCGTGA | AAAGACTGCTCGACTTGGGG  GCTGGCGGAGCTGAATATCT  CTCCGTCCTGTCTGCAATGT  ATTGCCTCACAGAGTGGACG  GTCCAGTTCAGTGAGGCTCC  TCTTGGCCGAGGACTAAGGA  TCCTTTGGGGTTGCCTGTAG  CAGACGTGGGACCAAAGAGACTAT  CCTATATTCTCCGCATCATGCA  ATCCCAGAACCGTTGGTGAC  GTGTCCTCATTGACCTTGACCAGA  GGTACCGAAGCAGTTCACCA  GAGCCCCTCCCTCAAGTGGAAGGATT  CATGCGGAATCCATAGTACACCAG  ACCAGCCGTGACTTTACCAG  TCCTTCGGTAGCTCCGTACA  TGGGTAATCCATAGAGCCCAG  AAAGGAGGCTGCGTCTGTG  GGGTTTATGAAACTAGCCAC  GCGTTCCATGCTGATGCCCG  AGGTCCGAAATGGTCAGGTT  CCAAATCGCCTCTCCAG  ACAGAAAGCCAGTCGCTTGA  TGGTTTTGGATGTCGTGGG  CTTTGGAATGACGCGGTTCC  TCTGGACGCGTGTGAAGCT  CCTGTCTGTCTGAGCACAGT  GAGGCCTATGAATCGGTT  GCCACTGATTAGGGACTCGG  CACTTCCCTGCGGTAGCAT  CAGTGGAGTCCTTTCCGCAT  GTCATGGTCTCCAGTTCGCA  GCCTGGCATTAGCATGATGG  GACCTTGGAGCACCCTTTGG  ACACATTCCACCACCAGCTT  GCAGACCGTCTAGCTCTTTCAT  TGCTTACGCGCTCATAGGTC  TGCAGTCAGCATCCTCTTGT  GCTCCCAAGTCAGGAGCATT  ACCTGAAGGAGACGGAGACA  GTACCGTCACAACCTCCCTG  CACGTCCAGTGCTCGGGG  TTCACAGACAGCAAGTGGCA  CTCAGAGCTGAGTGCCCTTG  AAGGGAGAATGGCGACTTGG | Integrated DNA Technologies, Inc. |

- 1. **Biological samples**

| **Description** | **Source** | **Identifier** |
| --- | --- | --- |
| Liver tissue and blood of patients underwent liver transplantation | Human | Queen Mary Hospital, Hong Kong |

- 1. **Deposited data**

| **Name of repository** | **Identifier** |  | **Link** |
| --- | --- | --- | --- |
| NCBI’s Gene Expression Omnibus | GEO GSE204919 |  | https://www.ncbi.nlm.nih.gov/geo/query/acc.cgi?acc=GSE204919 |

- 1. **Software**

| **Software name** | **Manufacturer** | **Version** |
| --- | --- | --- |
| SPSS  Prism | SPSS, Inc  Graphpad software | 18 8 |

- 1. **Other (e.g. drugs, proteins, vectors etc.)**

| **Name** | **Cat no.** | **Supplier** | **Citations** |
| --- | --- | --- | --- |
| Lipofermata | HY-116788 | MedChemExpress | 26394026 |
| IL-1β recombinant protein | 575104 | Biolegend | 31853061 |

- 1. **Please provide the details of the corresponding methods author for the manuscript:**

| Further information and requests for resources will be followed by Prof. Man (kwanman@hku.hk) and Dr. Liu (jadekoala@sina.com). |
| --- |

**2.0 Please confirm for randomised controlled trials all versions of the clinical protocol are included in the submission. These will be published online as supplementary information.**

| Nil. |
| --- |
