# Supplementary material for: Arachidonic acid activates NLRP3 inflammasome in MDSCs via FATP2 to promote post-transplant tumour recurrence in steatotic liver grafts
Source: JHEP Rep. 2023 Aug 22;5(12):100895. doi: 10.1016/j.jhepr.2023.100895 (PMC10616418; doi:10.1016/j.jhepr.2023.100895)
Supplement: Multimedia component 4 [file mmc4.pdf]

Research article

JHEP Reports

Arachidonic acid activates NLRP3 inflammasome in MDSCs via FATP2 to promote post-transplant tumour recurrence in steatotic liver grafts

Hui Liu,[1,2] Wai Ho Oscar Yeung,[1] Li Pang,[1] Jiang Liu,[1] Xiao Bing Liu,[1] Kevin Tak Pan Ng,[1] Qingmei Zhang,[1] Wen Qi Qiu,[1] Yueqin Zhu,[1] Tao Ding,[1] Zhe Wang,[1] Ji Ye Zhu,[1] Chung Mau Lo,[1] Kwan Man[1,*]

[1]*Department of Surgery, School of Clinical Medicine, HKU-SZH and LKS Faculty of Medicine, The University of Hong Kong, Hong Kong, China;* [2]*Department of Pathophysiology, Key Laboratory of Cell Differentiation and Apoptosis of the Chinese Ministry of Education, Shanghai Jiao Tong University School of Medicine, Shanghai, China*

JHEP Reports **2023.** https://doi.org/10.1016/j.jhepr.2023.100895

Background & Aims: The steatotic grafts have been applied in liver transplantation frequently owing to the high incidence of non-alcoholic fatty liver disease. However, fatty livers are vulnerable to graft injury. Myeloid-derived suppressor cell (MDSC) recruitment during liver graft injury promotes tumour recurrence. Lipid metabolism exerts the immunological influence on MDSCs in tumour progression. Here, we aimed to explore the role and mechanism of inflammasome activation in MDSCs induced by lipid metabolism during fatty liver graft injury and the subsequent effects on tumour recurrence.
Methods: MDSC populations and nucleotide-binding oligomerisation domain-like receptor family pyrin domain containing 3 (NLRP3) inflammasome levels were investigated in a clinical cohort and a rat liver transplantation model. The mechanism of NLRP3 activation by specific fatty acids was explored in mouse hepatic ischaemia/reperfusion injury (IRI) with tumour recurrence model and *in vitro* studies.
Results: MDSC populations and NLRP3 levels were increased with higher tumour recurrent rate in patients using steatotic grafts. NLRP3 was upregulated in MDSCs with lipid accumulation post mouse fatty liver IRI. Mechanistically, arachidonic acid was discovered to activate NLRP3 inflammasome in MDSCs through fatty acid transport protein 2 (FATP2), which was identified by screening lipid uptake receptors. The mitochondrial dysfunction with enhanced reactive oxygen species bridged arachidonic acid uptake and NLRP3 activation in MDSCs, which subsequently stimulated $CD4^+$ T cells producing more IL-17 in fatty liver IRI. Blockade of FATP2 inhibited NLRP3 activation in MDSCs, IL-17 production in $CD4^+$ T cells, and the tumour recurrence post fatty liver IRI.
Conclusions: During fatty liver graft injury, arachidonic acid activated NLRP3 inflammasome in MDSCs through FATP2, which subsequently stimulated $CD4^+$ T cells producing IL-17 to promote tumour recurrence post transplantation.
Impact and implications: The high incidence of non-alcoholic fatty liver disease resulted in the frequent application of steatotic donors in liver transplantation. Our data showed that the patients who underwent liver transplantation using fatty grafts experienced higher tumour recurrence. We found that arachidonic acid activated NLRP3 inflammasome in MDSCs through FATP2 during fatty liver graft injury, which led to more IL-17 secretion of $CD4^+$ T cells and promoted tumour recurrence post transplantation. The inflammasome activation by aberrant fatty acid metabolism in MDSCs bridged the acute-phase fatty liver graft injury and liver tumour recurrence.
© 2023 The Authors. Published by Elsevier B.V. on behalf of European Association for the Study of the Liver (EASL). This is an open access article under the CC BY license (http://creativecommons.org/licenses/by/4.0/).

Introduction

The drastic donor shortage is still a huge problem for liver transplantation. The high incidence of non-alcoholic fatty liver disease resulted in the frequent application of steatotic donor livers.[1–3] However, fatty donor livers are vulnerable to graft injury.[4,5] For transplant recipients with hepatocellular carcinoma (HCC), graft injury may reshape the graft immune microenvironment through recruiting several immunosuppressive cells, such as regulatory T cells and myeloid-derived suppressor cells (MDSCs), which subsequently promote tumour recurrence.[5–8] Our previous study found the tendency of poor recurrence-free survival in patients who received graft with >10% steatosis after living-donor liver transplantation.[4] Nevertheless, the responses of immune cells to steatotic liver graft injury and their roles and mechanisms for liver tumour recurrence have not been well illustrated.

MDSCs represent a heterogeneous population of myeloid progenitor cells that not only disrupt the surveillance of tumours by crosstalking with other immune cells[9–12] but also directly promote cancer stemness, angiogenesis, and metastasis.[13] Recent

Keywords: Steatotic liver graft; Tumour recurrence; MDSC; Inflammasome; Lipid metabolism.
Received 10 March 2023; received in revised form 6 July 2023; accepted 16 August 2023; available online 22 August 2023
* Corresponding author. Address: Department of Surgery, School of Clinical Medicine, HKU-SZH and LKS Faculty of Medicine, The University of Hong Kong, L9-55, 21 Sassoon Road, Hong Kong, China. Tel.: +852-39179646; Fax: +852-39179634.
E-mail address: kwanman@hku.hk (K. Man).

ELSEVIER

EASL The Home of Hepatology

studies reported that the immune function of MDSCs could be modulated by lipid metabolism. The uptake and oxidation of fatty acid increases in tumour-infiltrating MDSCs, and the inhibition of fatty acid oxidation enhances the cancer therapies.[14] Moreover, the tumour-derived factors facilitate lipid uptake, oxidative metabolism, and tolerogenic functional reprogramming of MDSCs.[15] In patients with cancer, the specific marker of granulocytic MDSCs (G-MDSCs) is associated with endoplasmic reticulum stress and lipid metabolism.[16] G-MDSCs acquired immunosuppressive activity mediated by fatty acid transport protein 2 (FATP2).[17] Our previous findings indicated that MDSCs were recruited into liver graft during acute-phase injury, promoting tumour recurrence post transplantation.[18] However, the roles and mechanisms of lipid metabolism in MDSCs in steatotic liver graft injury have never been explored.

As pattern recognition receptors to sense the danger signals, inflammasomes process the release of bioactive IL-1β and IL-18 via caspase-1 activation and induce cell pyroptosis.[19] Inflammasome activation plays critical roles in liver diseases.[20] In non-alcoholic steatohepatitis, saturate fatty acids activate the inflammasomes in hepatocytes, and the danger signals released by hepatocytes could further trigger the inflammasome activation in immune cells.[21] Blockade of nucleotide-binding oligomerisation domain-like receptor family pyrin domain containing 3 (NLRP3) inflammasome reduces the liver inflammation and fibrosis in a mouse non-alcoholic steatohepatitis model.[22] We reported that NLRP3 inflammasome activation in neutrophils induced liver graft injury via the telomere-independent repressor activator protein 1 (RAP1)/keratinocyte chemoattractant (KC) axis post transplantation.[23] Gene silencing of NLRP3 inflammasome may protect against liver ischaemia/reperfusion injury (IRI).[24] Decreased NLRP3 inflammasome expression has been found in HCC tissues compared with non-cancerous tissues although its role in tumorigenesis can be opposing.[25] In MDSCs, NLRP3 inflammasome is activated via cathepsin B release triggered by chemotherapy and curtails anticancer immunity.[26] However, the role of fatty graft injury in inflammasome activation in MDSCs and on tumour recurrence has not been explored.

In the present study, we aimed to investigate the role and mechanism of inflammasome activation in MDSCs induced by acute-phase injury of steatotic liver graft and its subsequent effect on promoting tumour recurrence. Our data indicated that NLRP3 inflammasome in MDSCs was activated by arachidonic acid through FATP2 during fatty graft injury, and it further induced the IL-17 production of $CD4^+$ T cells, promoting the tumour recurrence post liver transplantation.

Materials and methods

Clinical cohort and biopsies

Eighty-eight patients with HCC from 1997 to 2017 who underwent living-donor liver transplantation in Queen Mary Hospital, The University of Hong Kong, were included in our study. Among them, eight patients received fatty donor livers (fatty change >10%), whereas the other 80 patients received normal livers. The graft biopsies and peripheral blood were prospectively collected at 2 h and 7 days after portal vein reperfusion, respectively. Signed consent forms were acquired from each donor and recipient patient before operation. The procedures followed in the study conformed to the ethical standards of the Helsinki Declaration of 1975, as revised in 1983, and approved by the institutional review board of The University of Hong Kong.

Animal models

Sprague Dawley rats (male, 6–8 weeks, 180–220 g) and C57 BL/6 mice (male, 6–8 weeks, 20–25 g) were purchased from the Laboratory Animal Unit, The University of Hong Kong. Both rats and mice were housed in a standard animal facility at 22 ± 2 °C under controlled 12-h light/dark cycles and had free access to chow and autoclaved water. The animals with poor physical conditions were excluded. All animals received humane care according to the criteria outlined in *Guide for the Care and Use of Laboratory Animals (National Institutes Health publication 86–23, 1985 revision)*. Experimental protocols were approved by the Committee on the Use of Live Animals in Teaching and Research, The University of Hong Kong.

Rat orthotopic liver transplantation model

The steatotic donor rats were fed with 45% high-fat diet (58G8, TestDiet, Land O'Lakes, USA), whereas the normal donor rats were fed with regular diet for 2 weeks (about 50% steatosis). The recipient rats were injected with carbon tetrachloride (CCl_4, 2 ml/kg) s.c. for 4 weeks to induce liver cirrhosis before operation. The orthotopic liver transplantation model was established using small-for-size grafts (ratio of graft weight to recipient liver weight was about 50%) in Sprague Dawley rats. Blood and liver tissues were harvested at 2, 6, and 24 h after transplantation. The procedures were implemented according to the previous protocols.[27,28]

Mouse hepatic IRI and tumour recurrence model

The mice in the fatty liver group were fed with 45% high-fat diet for 3 weeks before operation. According to our preliminary experiment, five animals were chosen in each group (n = 5). All the animals were randomly grouping with no blinding. The animals with poor physical conditions were excluded. To mimic the graft injury, mice were subjected to liver IRI.[29] The right triangular and right middle lobes (about 30%) of the liver underwent ischaemia for 45 min with a vascular clamp. Injured liver and spleen tissues were collected at 12 h after reperfusion. Lipofermata (an FATP2 inhibitor, 2 mg/kg, MedChemExpress, NJ, USA) was injected i.p. in the treatment group 1 day in advance and immediately before the operation start.

The liver tumour cells (Hepa1-6, ATCC, 1.5 × 10^6/100 µl per mouse) were injected into the portal vein immediately after reperfusion to establish the mouse liver tumour recurrence model. The cell line has been authenticated. Lipofermata (2 mg/kg) was injected i.p. 1 day in advance, immediately before the operation, and every 2 days after the reperfusion in the treatment group. IL-1β recombinant protein (10 µg/kg; BioLegend, San Diego, CA, USA) was injected i.p. immediately after the reperfusion and every 2 days after the operation based on lipofermata treatment in the rescue group. At 14 days post operation, liver and spleen tissues were harvested for analysis. The tumour size was measured by the luminescent intensity after injection of luciferase (IVIS Spectrum Imaging System, PerkinElmer, Waltham, MA, USA).

Primary MDSC and naive $CD4^+$ T-cell isolation by magnetic bead cell sorting from mouse

Primary MDSCs and naive $CD4^+$ T cells were isolated from the mouse bone marrow and spleen, respectively. Bone marrow cells

were acquired from femurs and tibias. The splenic cells and bone marrow cells were flushed out using PBS, filtered through 70-μm cell strainers and treated with ammonium–chloride–potassium lysing buffer (Chem Cruz, Santa Cruz Biotechnology, Dallas, TX, USA). After obtaining the single cell suspension, $Gr1^+CD11b^+$ cells (MDSCs) and $CD4^+CD44^{low}CD62L^{high}$ cells (naive $CD4^+$ T cells) were isolated using mouse magnetic bead cell isolation kits (STEMCELL Technologies, Vancouver, BC, Canada) following the manufacturer's instruction. The purity was >90% via flowcytometry analysis. The cells were cultured using charcoal stripped FBS (Gibco, NY, USA) medium. MDSCs were stimulated by arachidonic acid (10–200 μM, Sigma-Aldrich, Darmstadt, Germany) or treated with lipofermata (50 μM) for 16 h. After wash, MDSCs were seeded in the upper chambers of 24-well Transwell inserts (0.4 μm, polyester membrane; SPL, Korea). Naive $CD4^+$ T cells, continuously stimulated with CD3/CD28 (25 μl/10^6 cells/ml; Gibco) and along with IL-2 (30 U; R&D Systems, Minneapolis, MN, USA), were seeded in the lower chambers with/without IL-1β recombinant protein (200 ng/μl). Before checking the production of IL-17, $CD4^+$ T cells were treated with a protein transport inhibitor (BD GolgiStop, BD Biosciences, San Jose, CA, USA) for 6 h.

Laboratory methods

Further details on quantitative real-time PCR, flow cytometry analysis, immunostaining, RNA sequencing (RNA-seq), gas chromatography–mass spectrometry (GC-MS) for fatty acids, and Western blot can be found in the Supplementary Materials and Methods.

Statistical analysis

Comparison was performed using Student's *t* test. Clinical survival was analysed using the Kaplan–Meier test. Degrees of statistical significance were demonstrated using standardised asterisk nomenclature (*p <0.05, **p <0.01, ***p <0.001). All analyses were implemented using SPSS 18.0 (SPSS, Chicago, IL, USA) and GraphPad Prism 8.0 (GraphPad Software Inc, La Jolla, CA, USA).

Results

Increased MDSCs and NLRP3 were associated with more HCC recurrence after transplantation using steatotic liver graft

Eighty-eight patients with HCC who received living-donor liver transplantation were involved in this study. HCC recurrence occurred in 21 patients after transplantation. The survival analysis indicated that the recipients implanted with steatotic liver grafts had relatively poor disease-free survival compared with patients with normal grafts (p = 0.031) (Fig. S1A). The multivariate analysis further certified that fatty change influenced the tumour recurrence (p = 0.044) (Table S1). More circulatory (p =

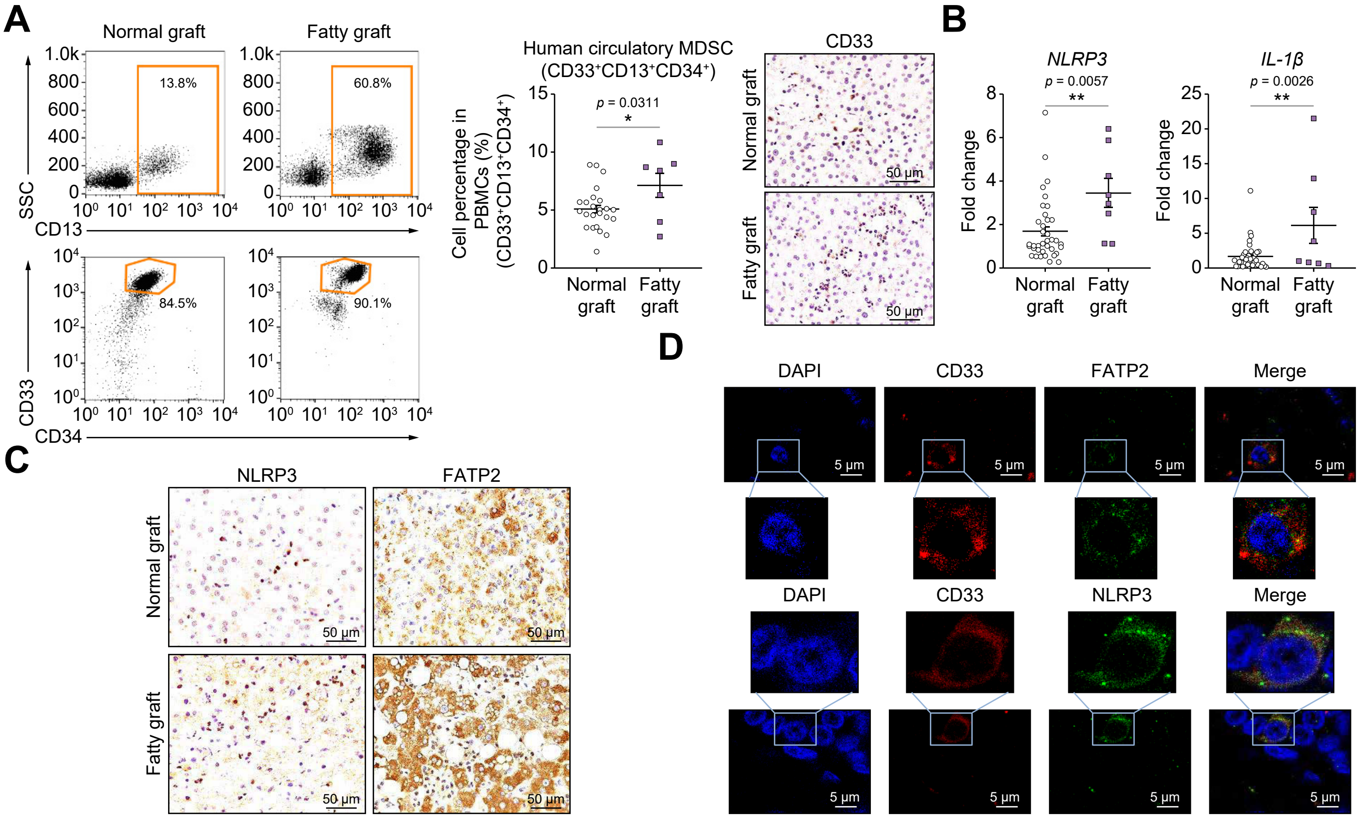

Fig. 1. MDSC populations and NLRP3 levels were increased in patients with HCC who received fatty grafts post liver transplantation. (A) Circulatory and intragraft MDSCs were increased in patients who received fatty grafts compared with those with normal liver grafts (n = 45). Scale bars: 50 μm. (B) The mRNA expressions of NLRP3 and IL-1β were higher in fatty liver grafts (n = 31). (C) More infiltrated NLRP3- and FATP2-positive cells were found in patients who received fatty liver grafts (n = 23). Scale bars: 50 μm. (D) CD33 (a MDSC marker) was colocalised with FATP2 and NLRP3 (n = 23). Scale bars: 5 μm. Error bars indicate SEM; *p <0.05, **p <0.01. FATP2, fatty acid transport protein 2; HCC, hepatocellular carcinoma; MDSC, myeloid-derived suppressor cell; NLRP3, nucleotide-binding oligomerisation domain-like receptor family pyrin domain containing 3; PBMC, peripheral blood mononuclear cell; SSC: side scatter.

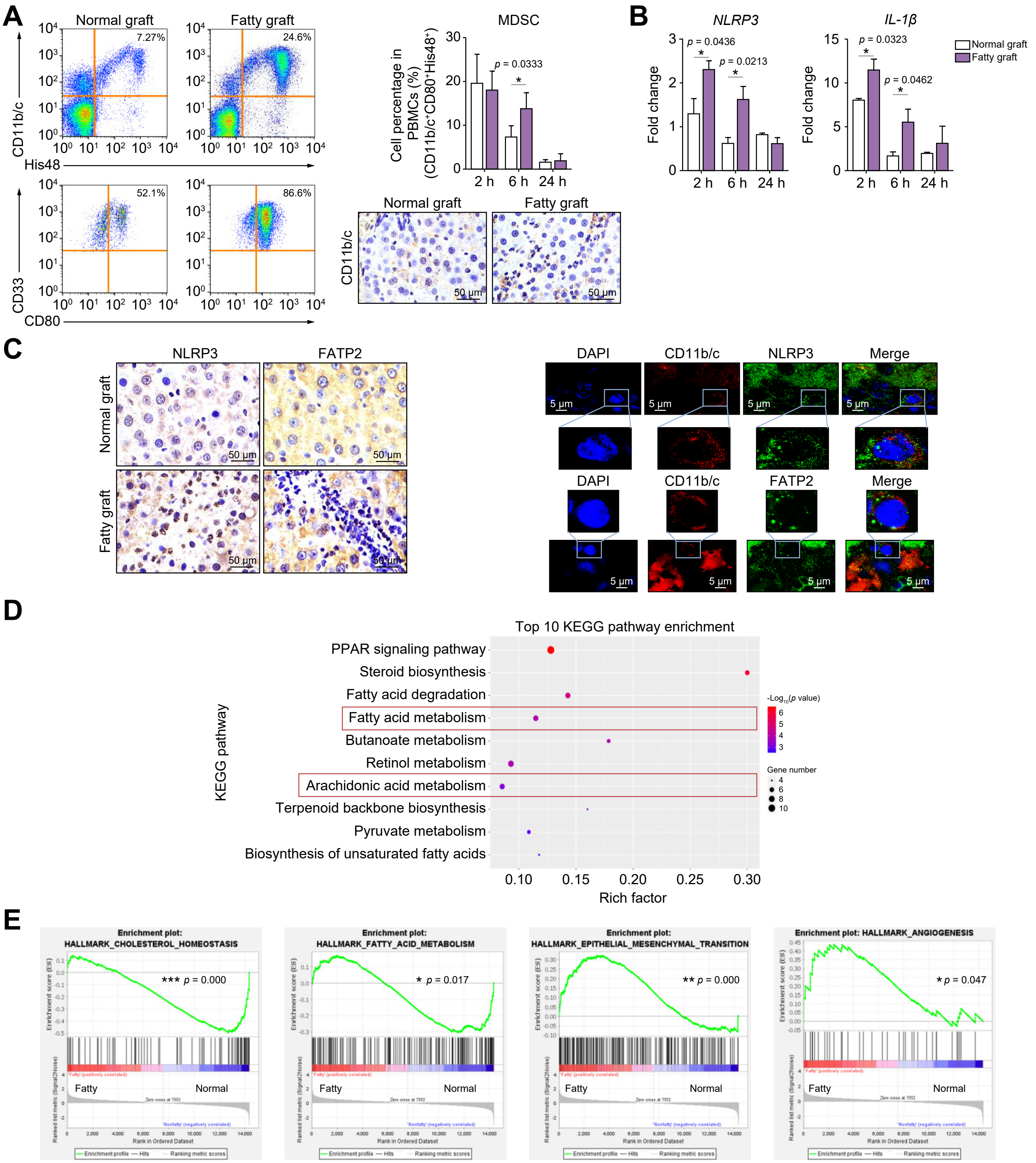

Fig. 2. Higher MDSCs and NLRP3 were accompanied with aberrant lipid metabolism and tumour-favouring alteration in fatty grafts post rat liver transplantation. (A) More MDSCs were found in rats implanted with fatty liver grafts. Scale bars: 50 μm. (B) The mRNA expressions of NLRP3 and IL-1β were increased in fatty liver grafts. (C) NLRP3- and FATP2-positive cells were more infiltrated in steatotic grafts and colocalised with CD11b/c (an MDSC marker). Scale bars: 50 μm (left) and 5 μm (right). (D) The lipid metabolism (including arachidonic acid metabolism) was aberrant in fatty grafts through KEGG analysis of RNA-seq post transplantation. (E) Cholesterol homoeostasis, fatty acid metabolism, epithelial mesenchymal transition, and angiogenesis were significantly changed in steatotic grafts by GSEA. (A–C) n = 4/group; (D and E) n = 3/group. Error bars indicate SEM; *p <0.05, **p <0.01, ***p <0.001. FATP2, fatty acid transport protein 2; GSEA, gene set enrichment analysis; KEGG, Kyoto Encyclopedia of Genes and Genomes; MDSC, myeloid-derived suppressor cell; NLRP3, nucleotide-binding oligomerisation domain-like receptor family pyrin domain containing 3; PBMC, peripheral blood mononuclear cell; RNA-seq, RNA sequencing.

0.0311) and intragraft MDSCs (CD33$^+$, p <0.0001; CD15$^+$ and LOX1$^+$, p = 0.0005) were found in the patients with fatty grafts (Fig. 1A and Fig. S1B–D). Our previous study indicated that the patients with high MDSCs had poor disease-free survival.[18] These findings suggested that MDSCs may play critical roles in tumour recurrence post liver transplantation using steatotic donors. Our previous report demonstrated that NLRP3 was the major type of inflammasome that was upregulated post transplantation.[23] In the present study, the patients implanted with fatty grafts had higher mRNA levels of intragraft NLRP3 (p = 0.0057) and IL-1β (p = 0.0026) (Fig. 1B). More infiltrated NLRP3 (p = 0.0011) and FATP2 (p <0.0001) positive cells were detected in fatty liver grafts (Fig. 1C and Fig. S1E). The colocalisation and accumulation of NLRP3 and FATP2 with CD33 in fatty grafts demonstrated that NLRP3 and FATP2 might modulate MDSCs in post-transplant tumour recurrence (Fig. 1D and Fig. S1F).

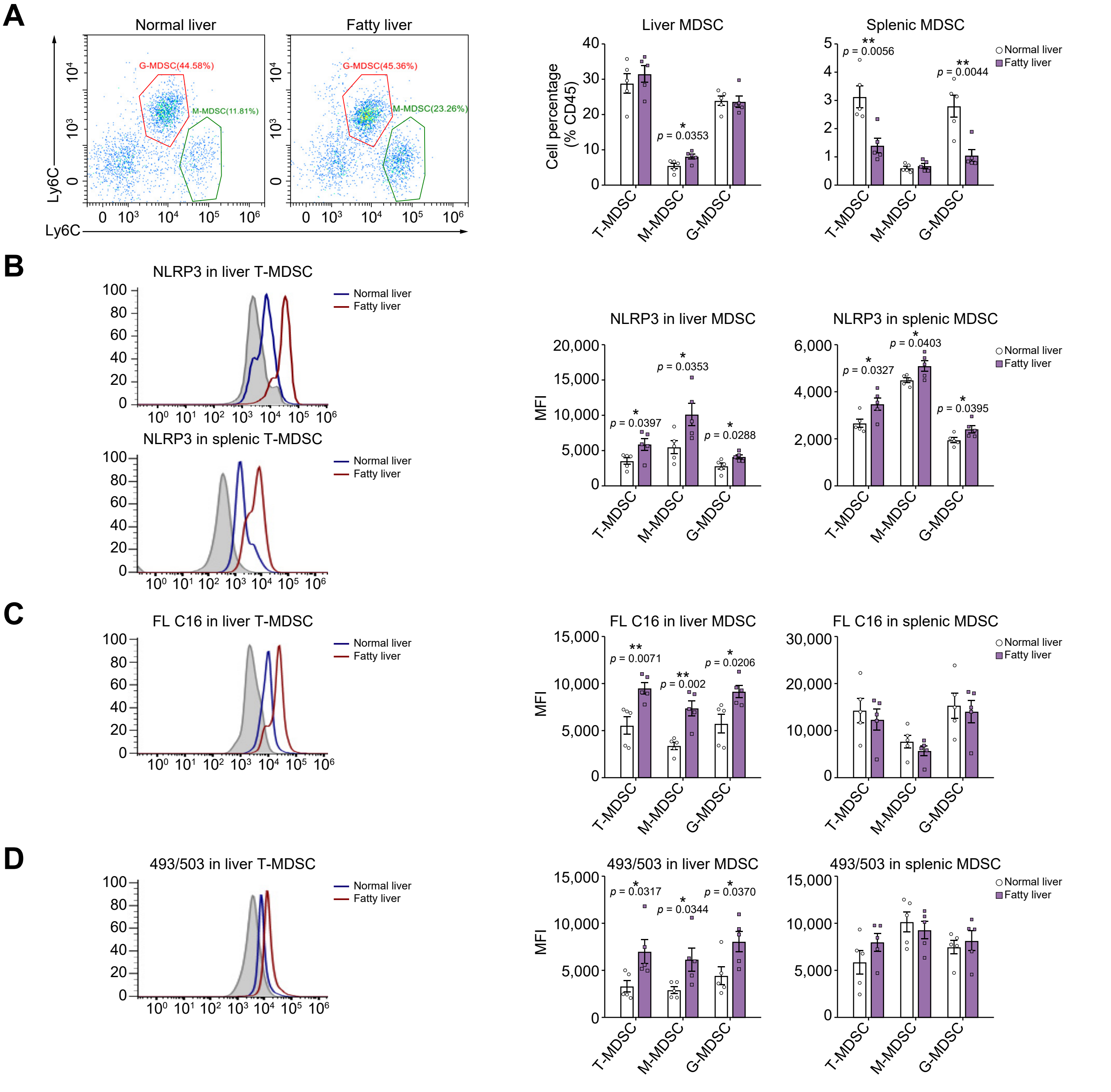

Fig. 3. Accumulated lipids enhanced NLRP3 in MDSCs of mouse fatty liver post IRI. (A) More M-MDSCs were accumulated in mouse fatty liver after IRI. (B) NLRP3 was upregulated in liver and splenic MDSCs post fatty liver IRI. (C) More FL C16 was absorbed by MDSCs in fatty liver. (D) The neutral lipids (493/503) were accumulated in MDSCs of fatty liver post IRI. (A–D) n = 5/group. Error bars indicate SEM; *p <0.05, **p <0.01. G-MDSC, granulocytic MDSC; IRI, ischaemia/reperfusion injury; M-MDSC, monocytic MDSC; MDSC, myeloid-derived suppressor cell; MFI, mean fluorescence intensity; NLRP3, nucleotide-binding oligomerisation domain-like receptor family pyrin domain containing 3; T-MDSC, total MDSC.

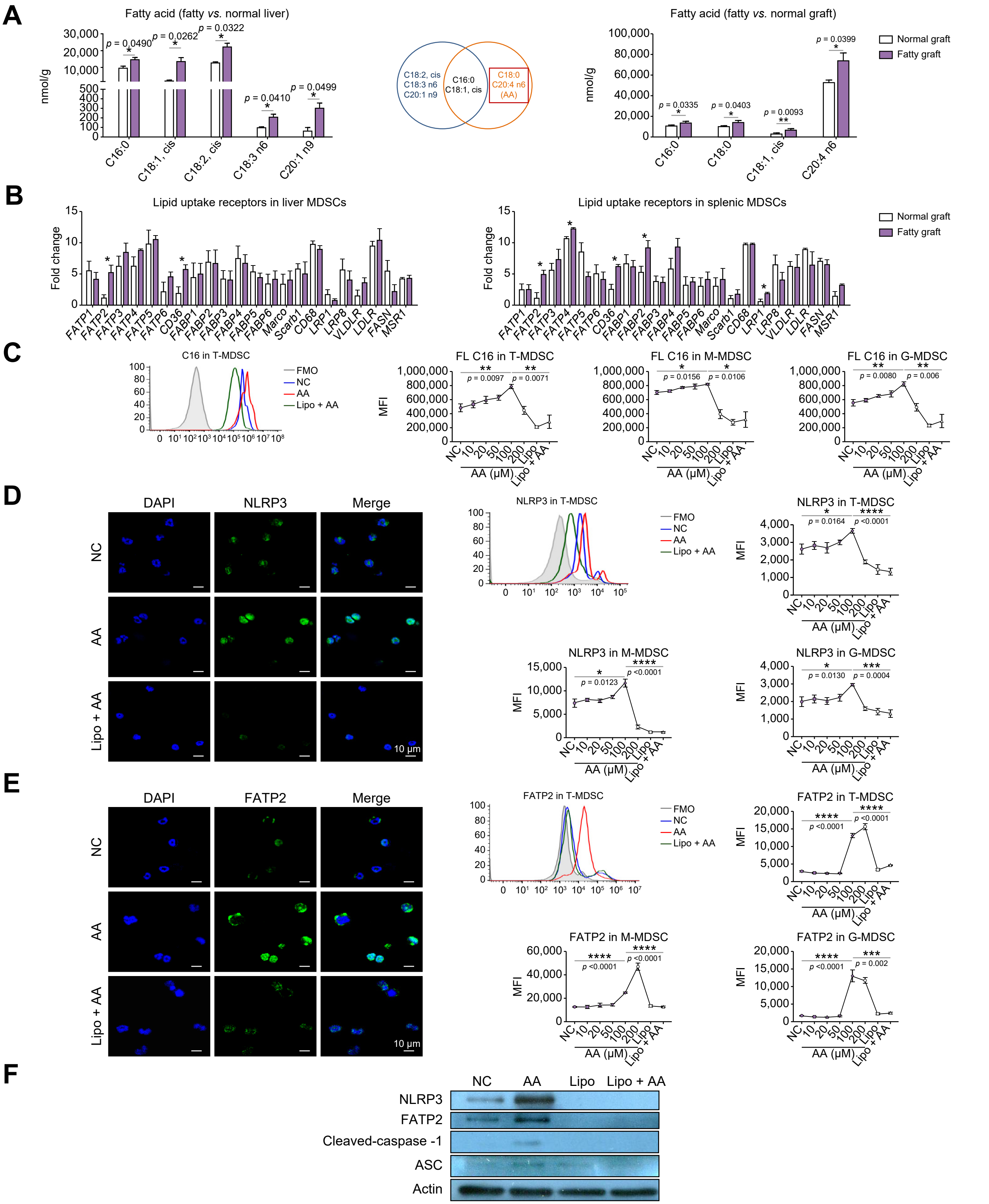

Fig. 4. Arachidonic acid activated NLRP3 inflammasome in MDSCs through FATP2. (A) C18:0 and C20:4 n6 (AA) were screened out in rat fatty *vs.* normal liver graft after getting rid of the fatty acids as a result of the diet by GC-MS (n = 4/group). (B) FATP2 and CD36 mRNA levels were upregulated in both liver and splenic MDSCs post mouse fatty liver IRI (n = 5/group). (C) FL C16 was augmented by AA stimulation but inhibited by Lipo (an FATP2 inhibitor) in primary MDSCs. (D) AA increased NLRP3, which was reduced by Lipo in primary MDSCs. (E) FATP2 was accumulated in MDSCs by AA stimulation but decreased by Lipo. (F) FATP2 and

The aberrant lipid metabolism and tumour-favouring alteration in steatotic grafts were accompanied with higher MDSCs and NLRP3 post rat liver transplantation

To avoid the confounding factors in clinical cohorts and to further investigate the alteration of MDSCs, we established a rat orthotopic liver transplantation model using fatty or normal small-for-size grafts. Consistent with the clinical results, circulatory (p = 0.0333) and intragraft MDSCs (CD11b/c$^+$, p = 0.0045; CD11b/c and His48, p = 0.0032) were significantly increased in the recipient rats with fatty donors at 6 h after reperfusion (Fig. 2A and Fig. S2A–C). By screening the inflammasome types, NLRP3 (p = 0.0213) and IL-1β (p = 0.0462) mRNA expressions were significantly upregulated in fatty grafts at Hour 6 post transplantation (Fig. 2B and Fig. S2D). More infiltrated NLRP3 (p = 0.0059) and FATP2 (p = 0.0067) positive cells and the colocalisation with CD11b/c (an MDSC marker) were found in rat fatty liver grafts (Fig. 2C and Fig. S2E and F). To further explore the different changes between normal and fatty grafts post transplantation, RNA-seq was performed. According to the Kyoto Encyclopedia of Genes and Genomes (KEGG) analysis, the top 10 pathways were related to lipid metabolism, including fatty acid and arachidonic acid metabolism (Fig. 2D). In addition to the significant change of cholesterol homoeostasis (p = 0.000) and fatty acid metabolism (p = 0.017), gene set enrichment analysis (GSEA) demonstrated that epithelial mesenchymal transition (p = 0.000) and angiogenesis (p = 0.047) were significantly enhanced in fatty grafts (Fig. 2E). These results indicated that aberrant lipid metabolism was critical in fatty grafts, which might promote the tumour-favouring microenvironment formation post transplantation.

NLRP3 was upregulated in MDSCs with more lipid uptake in fatty liver of mouse IRI model

The mouse hepatic IRI model with a fatty/normal liver was established to investigate the mechanism of NLRP3 activation in MDSCs. Briefly, the right triangular and right middle lobes of the liver underwent ischaemia for 45 min with a vascular clamp. Injured liver and spleen tissues were collected 12 h after reperfusion. The inflammatory markers of liver injury, including monocyte chemoattractant protein 1 (MCP-1), IL-6, C–X–C motif chemokine ligand 1 (CXCL1), interferon-γ (IFN-γ), IL-1β, and Toll-like receptor 4 (TLR4), were significantly upregulated in the fatty liver post IRI (Fig. S3A). In addition, alanine transaminase and aspartate aminotransferase were significantly increased in fatty liver after IRI (Fig. S3B). This verified the finding that the inflammatory injury was more severe in the fatty graft post liver transplantation. Regarding the MDSCs in the mouse liver IRI model, previous studies mainly focused on their mobilisation upon the surgical stress.[18,30] Our preliminary data demonstrated that hepatic and splenic MDSCs were obviously accumulated post normal liver IRI in contrast with sham control, whereas NLRP3 levels in hepatic MDSCs were not significantly increased. In the current study, the hepatic NLRP3 (Hour 2: p = 0.0465; Hour 6: p = 0.0077) and IL-1β (Hour 2: p = 0.0482; Hour 6: p = 0.0141) mRNA levels were significantly upregulated at the early stage after fatty liver IRI (Fig. S3C). Interestingly, only monocytic MDSCs (M-MDSCs; p = 0.0353) were significantly accumulated in the fatty liver after IRI (Fig. 3A and Fig. S4A). Importantly, NLRP3 was increased in total MDSCs (T), M-MDSCs (M), and G-MDSCs (G) of the liver (T: p = 0.0397; M: p = 0.0353; G: p = 0.0288) and spleen (T: p = 0.0327; M: p = 0.0403; G: p = 0.0395) post fatty liver IRI (Fig. 3B). The lipid uptake was further detected in MDSCs. FL C16 was significantly increased in MDSCs (T: p = 0.0071; M: p = 0.002; G: p = 0.0206) of the fatty liver with no obvious change in the spleen (Fig. 3C). Moreover, MDSCs (T: p = 0.0317; M: p = 0.0344; G: p = 0.0370) accumulated more neutral lipids using 493/503 staining in the fatty liver post IRI (Fig. 3D). Dil-LDL and free fatty acid in MDSCs were not changed obviously during fatty liver IRI (Fig. S4B and C).

Arachidonic acid activated NLRP3 inflammasome in MDSCs through FATP2

The fatty acids in fatty and normal grafts of the rat model were analysed by GC-MS. C18:0 and C20:4 n6 (arachidonic acid) were identified through screening after getting rid of the changed fatty acids as a result of the diet (Fig. 4A). To identify the ones that might mediate the activation of NLRP3 by fatty acids, the mRNA levels of potential lipid uptake receptors (as listed) in splenic and hepatic MDSCs isolated from the mouse model were evaluated. FATP2 and CD36 (two lipid uptake receptors) was increased in both liver and spleen MDSCs after fatty liver IRI in contrast with normal ones (Fig. 4B). To further explore the inflammasome activation by fatty acid, MDSCs were sorted from the bone marrow. FL C16 was found significantly accumulated in MDSCs (T: p = 0.0097; M: p = 0.0156; G: p = 0.0080) in a dosage-dependent manner with arachidonic acid stimulation (<100 μM). Lipofermata, an FATP2 inhibitor, could significantly reduce the arachidonic acid uptake of MDSCs (T: p = 0.0071; M: p = 0.0106; G: p = 0.006) (Fig. 4C). Moreover, NLRP3 levels in MDSCs were significantly increased by arachidonic acid stimulation (T: p = 0.0164; M: p = 0.0123; G: p = 0.0130) and decreased by lipofermata (T: p <0.0001; M: p <0.0001; G: p = 0.0004), as demonstrated by immunostaining and flow cytometry analysis (Fig. 4D). However, C18:0 could not change the levels of FL C16, NLRP3, and CD36 in a consistent manner, and sulfosuccinimidyl oleate sodium (an inhibitor of CD36) treatment could not decrease their expressions (Fig. S5A–C). These results indicated that arachidonic acid could be absorbed through FATP2 in MDSCs, which further activated the NLRP3 inflammasome.

FATP2 in MDSCs was also significantly upregulated by arachidonic acid stimulation (T: p <0.0001; M: p <0.0001; G: p <0.0001) and diminished by its inhibitor (T: p <0.0001; M: p <0.0001; G: p = 0.002) (Fig. 4E). Interestingly, CD36 in MDSCs was increased by arachidonic acid (T: p = 0.0147; M: p = 0.0405; G: p = 0.0104) and could be also reduced by lipofermata (T: p = 0.0017; M: p = 0.0051; G: p = 0.002) (Fig. S6A). The results from Western blot confirmed that the FATP2/NLRP3 inflammasome activation pathway could be enhanced by arachidonic acid and

NLRP3 activation pathway was enhanced by AA and inhibited by FATP2 blockade through Western blot analysis. Scale bars: 10 μm. Error bars indicate SEM; *p <0.05, **p <0.01, ***p <0.001. AA, arachidonic acid; ASC, apoptosis-associated speck-like protein containing a caspase recruitment domain; FATP2, fatty acid transport protein 2; FMO, fluorescence minus one; G-MDSC, granulocytic MDSC; GC-MS, gas chromatography–mass spectrometry; IRI, ischaemia/reperfusion injury; Lipo, lipofermata; M-MDSC, monocytic MDSC; MDSC, myeloid-derived suppressor cell; MFI, mean fluorescence intensity; NC, negative control (the primary MDSCs without stimulation); NLRP3, nucleotide-binding oligomerisation domain-like receptor family pyrin domain containing 3; T-MDSC, total MDSC.

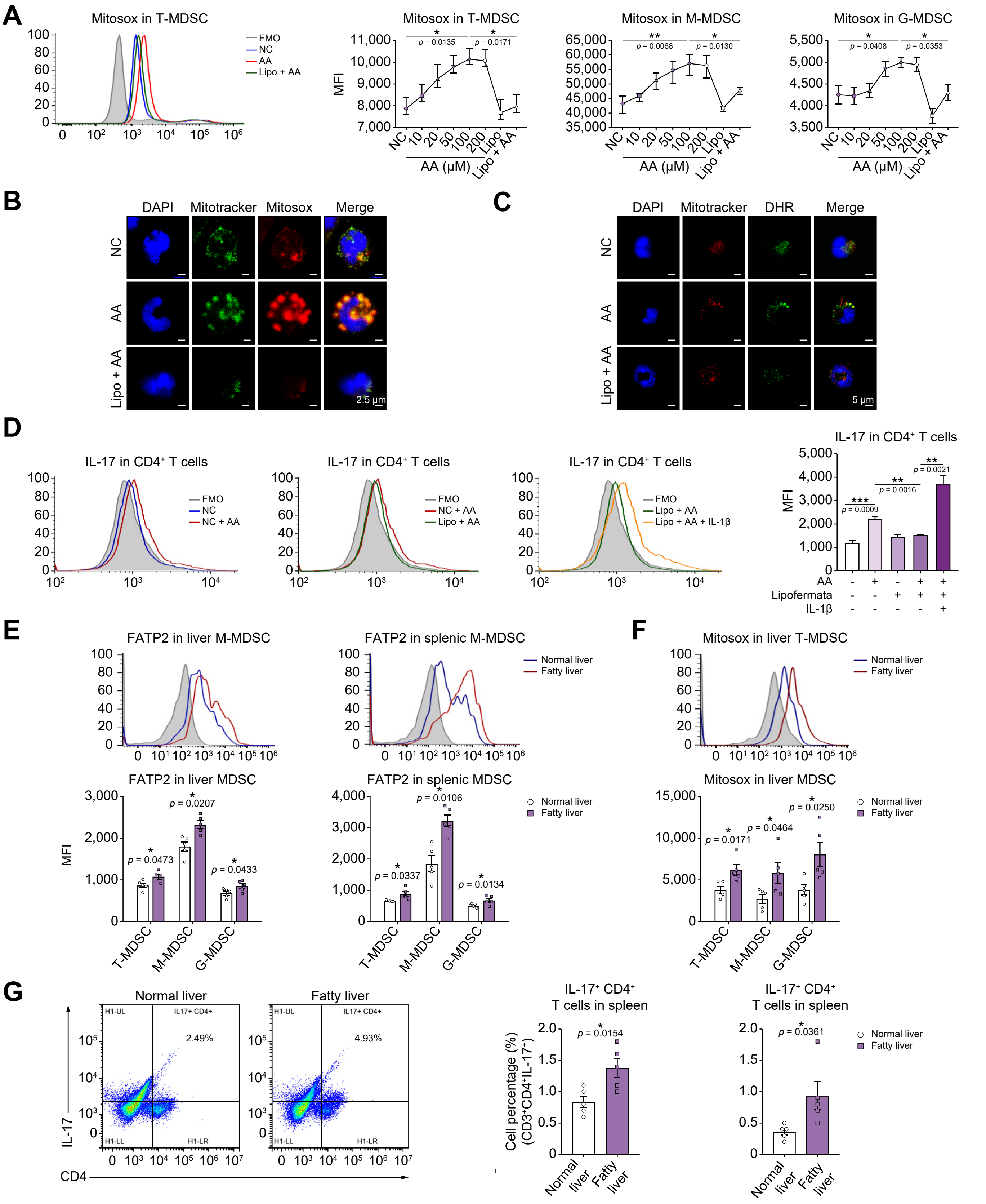

Fig. 5. NLRP3 inflammasome activation with mitochondrial dysfunction in MDSCs stimulated naive CD4$^+$ T cells producing IL-17. (A) AA increased the ROS (MitoSOX) of primary MDSCs, which was diminished by Lipo through flow cytometry analysis. (B) The MitoSOX was raised by AA and reduced by FATP2 inhibition by immunostaining. Scale bars: 2.5 μm. (C) DHR was enhanced and decreased by AA and Lipo, respectively. Scale bars: 5 μm. (D) The production of IL-17 in primary naive CD4$^+$ T cells was increased, decreased, and restored after coculture with MDSCs treated by AA, Lipo, and IL-1β recombinant protein, respectively. (E)

reduced by lipofermata (Fig. 4F). These data suggested that arachidonic acid could not only activate the NLRP3 inflammasome but also modulate the levels of lipid uptake receptors.

Activation of NLRP3 inflammasome with mitochondrial dysfunction in MDSCs stimulated CD4$^+$ T cells producing IL-17 *in vitro* and *in vivo*

Next, we explored the mechanisms of lipid uptake activating the NLRP3 inflammasome via FATP2 in MDSCs. The reactive oxygen species (ROS) production (mitochondrial superoxide indicator, MitoSOX), instead of mitochondrial counts (MitoTracker) in MDSCs, was significantly increased in a dosage-dependent manner (T: p = 0.0135; M: p = 0.0068; G: p = 0.0408). Moreover, the MitoSOX was decreased by lipofermata (T: p = 0.0171; M: p = 0.0130; G: p = 0.0353). Such phenotype was consistent with FL C16 and inflammasome expressions by arachidonic acid stimulation (Fig. 5A and Fig. S6B). The immunofluorescent staining further indicated that arachidonic acid enhanced the ROS of MDSCs, which might subsequently activate the NLRP3 inflammasome (Fig. 5B and C). The major mitochondrial protein levels, including NADH dehydrogenase (ubiquinone) 1 beta subcomplex subunit 8 (NDUFB8, complex I), succinate dehydrogenase B (SDHB, complex II), ubiquinol–cytochrome C reductase core protein 2 (UQCRC2, complex III), mitochondrially encoded cytochrome C oxidase I (MT-CO1, complex IV), ATP synthase alpha-subunit (ATP5A1, complex V), voltage-dependent anion channel (VDAC), prohibitin 1 (PHB1), and superoxide dismutase 1 (SOD1) in MDSCs were obviously downregulated by arachidonic acid stimulation but increased through the inhibition of FATP2. However, dynamin-related protein 1 (DRP1), the damage marker of mitochondria, was increased by arachidonic acid but reduced by FATP2 inhibition (Fig. S6C). Furthermore, we found that the ATPs produced by MDSCs were decreased by arachidonic acid (p = 0.0029) but enhanced by FATP2 blocking (p = 0.0004) (Fig. S6D). Therefore, the increased levels of ROS in MDSCs by arachidonic acid stimulation damaged the mitochondrial membrane and function, and diminished the production of ATPs.

The immune cell infiltration analysis from our RNA-seq data showed that CD4$^+$ T cells were increased in steatotic grafts post transplantation. The effects of MDSCs on CD4$^+$ T-cell differentiation were further explored. MDSCs were treated with arachidonic acid or lipofermata, and then cocultured with naive CD4$^+$ T cells isolated from the mouse spleen. The IL-17 levels in CD4$^+$ T cells were significantly increased (p = 0.0009) or reduced (p = 0.0016) after coculture with arachidonic acid- or lipofermata-treated MDSCs, respectively. Adding the IL-1β recombinant protein reversed the inhibition of lipofermata and raised much more IL-17 production of CD4$^+$ T cells (p = 0.0021) (Fig. 5D and Fig. S6E). The data indicated that bioactive IL-1β release through inflammasome activation in MDSCs caused naive CD4$^+$ T cells preferred to differentiate into T helper 17 (Th17) cells. An increase of Th17 cells was reported to promote HCC.[31] Accumulated MDSCs and Th17 cells may lead to the microenvironment of the fatty liver graft favouring tumour recurrence post transplantation.

Based on our *in vitro* findings, the FATP2 levels and mitochondrial function in MDSCs were further verified in mice. Consistent with NLRP3, FATP2 was significantly increased in MDSCs of the liver (T: p = 0.0473; M: p = 0.0207; G: p = 0.0433) and spleen (T: p = 0.0337; M: p = 0.0106; G: p = 0.0134) after fatty liver IRI (Fig. 5E). The ROS in MDSCs was significantly enhanced (T: p = 0.0171; M: p = 0.0464; G: p = 0.0250), whereas the counts of mitochondria were not obviously changed in fatty liver after IRI, which was echoed with the *in vitro* findings (Fig. 5F and Fig. S6F and G). Moreover, IL-17-positive CD4$^+$ T cells were significantly increased in the liver (p = 0.0154) and spleen (p = 0.0361) (Fig. 5G and Fig. S7A). CD36 in MDSCs was also upregulated in the liver (T: p = 0.0410; M: p = 0.0459; G: p = 0.0427) and spleen (T: p = 0.0034; M: p = 0.0004; G: p = 0.0405) (Fig. S7B). These data confirmed that NLRP3 inflammasome in MDSCs was activated through FATP2 with mitochondrial dysfunction and subsequently increased the IL-17 production of CD4$^+$ T cells.

Blockade of FATP2 inhibited NLRP3 activation in MDSCs and IL-17 production in CD4$^+$ T cells post fatty liver IRI in mice

As a lipid uptake receptor, FATP2 might be the target to reduce the NLRP3 activation in MDSCs. The MDSC populations in fatty liver were significantly decreased by lipofermata (an FATP2 inhibitor) treatment after IRI (T: p = 0.0476; M: p = 0.0153; G: p = 0.0393) (Fig. 6A and Fig. S4A). Inhibition of FATP2 reduced the NLRP3 levels only in M-MDSCs of the liver (p = 0.0244) and spleen (p = 0.0249) after fatty liver IRI (Fig. 6B). FATP2 in M-MDSCs was significantly diminished in both the liver (p = 0.0271) and the spleen (p = 0.0210) by lipofermata (Fig. 6C). CD36 expression was also downregulated in M-MDSCs of the liver (p = 0.0126) and spleen (p = 0.0419) (Fig. S7C). Consistently, FATP2 inhibition reduced the ROS in M-MDSCs of the liver (p = 0.0454) and spleen (p = 0.0190) (Fig. 6D). Furthermore, the population of IL-17-positive CD4$^+$ T cells in the fatty liver was significantly decreased by blockade of FATP2 (p = 0.0144) (Fig. 6E). These results suggested that FATP2 inhibition could effectively decrease NLRP3 inflammasome activation in MDSCs and IL-17 production in CD4$^+$ T cells during fatty liver IRI, which paved the way to study the effects of FATP2 blockade on tumour recurrence post transplantation.

Tumour recurrence was inhibited by therapeutic targeting of FATP2

To further explore the effects of targeting FATP2, mouse hepatic IRI with tumour recurrence models (the liver tumour cells were injected into the portal vein immediately after reperfusion) were established with four treatment groups: (1) normal liver, (2) fatty liver, (3) fatty liver + lipofermata, and (4) fatty liver + lipofermata + IL-1β recombinant protein. The tumour size was significantly increased (p = 0.0144) in the fatty liver and reduced by lipofermata treatment (p = 0.0063). IL-1β offset the inhibition

FATP2 in hepatic and splenic MDSCs was upregulated after fatty liver IRI in mice. (F) The ROS in MDSCs of the fatty liver was enhanced post IRI in mice. (G) The population of IL-17$^+$ CD4$^+$ T cells was accumulated after fatty liver IRI. (E–G) n = 5/group. Error bars indicate SEM; *p <0.05, **p <0.01, ***p <0.001. AA, arachidonic acid; DHR, dihydrorhodamine 123; FATP2, fatty acid transport protein 2; FMO, fluorescence minus one; G-MDSC, granulocytic MDSC; IRI, ischaemia/reperfusion injury; Lipo, lipofermata; M-MDSC, monocytic MDSC; MDSC, myeloid-derived suppressor cell; MFI, mean fluorescence intensity; NC, negative control (the primary MDSCs without stimulation); NLRP3, nucleotide-binding oligomerisation domain-like receptor family pyrin domain containing 3; ROS, reactive oxygen species; T-MDSC, total MDSC.

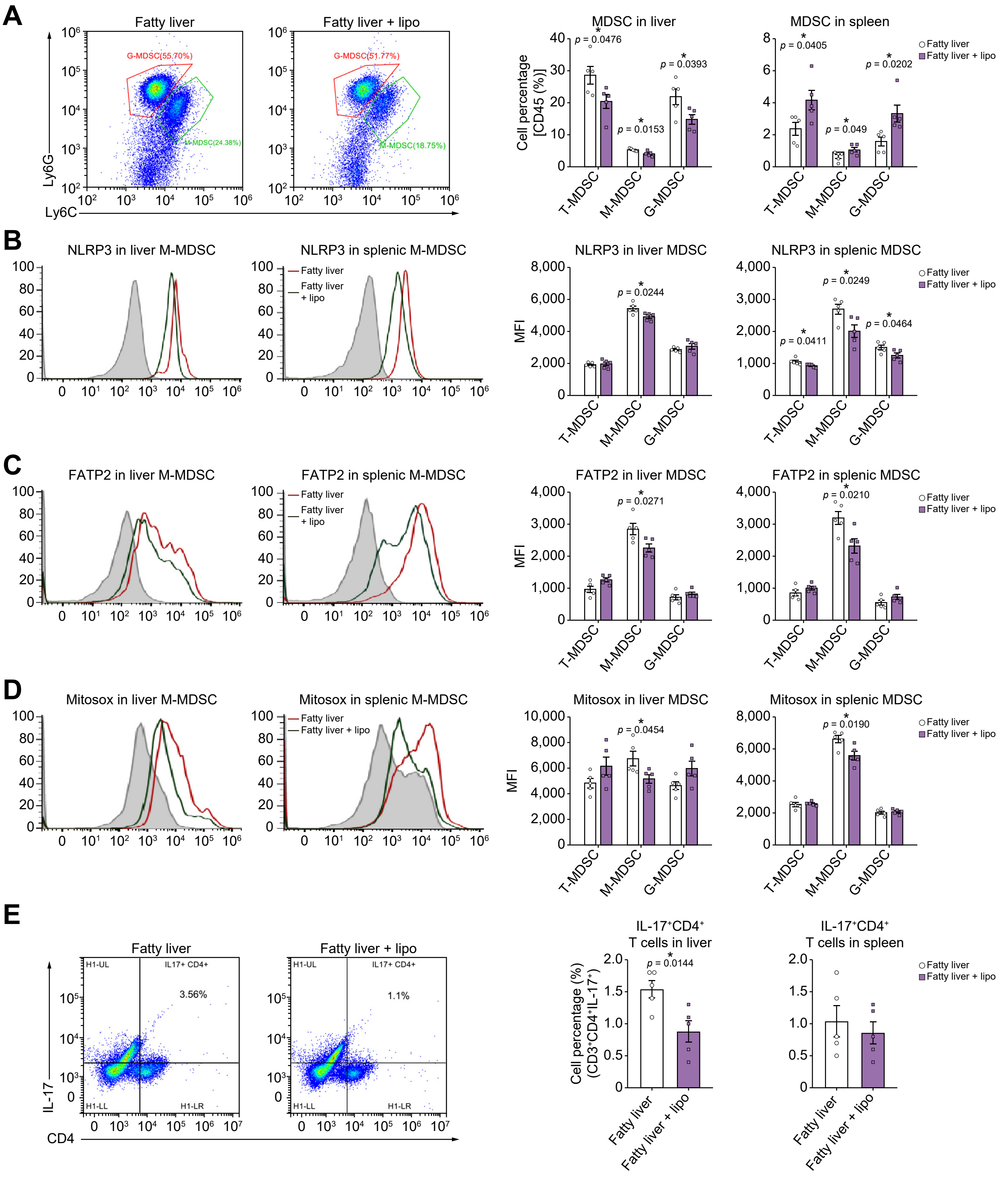

Fig. 6. FATP2 blockade diminished NLRP3 activation in MDSCs and IL-17 production in CD4$^+$ T cells after mouse fatty liver IRI. (A) The counts of hepatic MDSCs were reduced by FATP2 blockade after fatty liver IRI in mice. (B) NLRP3 in M-MDSCs of the fatty liver was decreased by FATP2 inhibition. (C) Lipo (an FATP2 inhibitor) reduced the FATP2 levels of M-MDSCs post fatty liver IRI. (D) ROS in M-MDSCs was diminished by FATP2 blockade. (E) The population of liver IL-17$^+$CD4$^+$ T cells was decreased by FATP2 inhibition. (A–E) n = 5/group. Error bars indicate SEM; **p* <0.05. FATP2, fatty acid transport protein 2; G-MDSC, granulocytic MDSC; IRI, ischaemia/reperfusion injury; Lipo, lipofermata; M-MDSC, monocytic MDSC; MDSC, myeloid-derived suppressor cell; MFI, mean fluorescence intensity; NLRP3, nucleotide-binding oligomerisation domain-like receptor family pyrin domain containing 3; ROS, reactive oxygen species; T-MDSC, total MDSC.

of lipofermata and expanded the tumour size through evaluating the luminescent intensity ($p = 0.0094$) (Fig. 7A and B). Moreover, the increased liver weight/body weight and spleen weight/body weight ratios in the fatty liver group were suppressed by FATP2 inhibition and raised by IL-1β (Fig. S8A). The immunohistochemistry staining showed that alpha foetoprotein and CD31-positive cells were accumulated in the fatty liver group, decreased by FATP2 inhibition and obviously increased by IL-1β treatment. These results demonstrated that not only the tumour burden but also the angiogenesis was increased in the fatty liver, and both were inhibited by FATP2 blockade and restored by IL-1β (Fig. S8B). Consistently, the hepatic IL-17^{+}CD4^{+} T cells were significantly accumulated ($p = 0.0401$) in the fatty liver group, decreased by FATP2 inhibition ($p = 0.0179$) and obviously increased by IL-1β ($p = 0.0175$) (Fig. 7C and Figs. S7A and S9A). Only the hepatic M-MDSCs were significantly increased ($p = 0.0259$) in the fatty liver but were decreased by FATP2 blockade ($p = 0.037$) (Fig. 7D and Fig. S9B and C). Moreover, NLRP3 in MDSCs was enhanced in both the liver (T: $p = 0.035$; M: $p = 0.0059$; G: $p = 0.0411$) and spleen (T: $p = 0.0296$; M: $p = 0.0269$) of the fatty liver group. Lipofermata reduced NLRP3 in MDSCs of the liver (T: $p = 0.0273$; M: $p = 0.0001$; G: $p = 0.0021$) and spleen (T: $p = 0.0466$; M: $p = 0.0269$; G: $p = 0.0053$). IL-1β recombinant protein inhibited NLRP3 more in MDSCs probably owing to the feedback of IL-1β (Fig. 7E and Fig. S9D).

Consistent with NLRP3, FATP2 in MDSCs was upregulated in both the liver (T: $p = 0.0219$; M: $p = 0.0397$; G: $p = 0.0445$) and spleen (T: $p = 0.0063$; M: $p = 0.0433$; G: $p = 0.0033$) of the fatty liver group. However, FATP2 was inhibited by lipofermata only in M-MDSCs of the liver ($p = 0.0420$) and spleen ($p = 0.0217$) (Fig. 7F and Fig. S9E). The ROS in MDSCs was also upregulated in the liver (T: $p = 0.039$; M: $p = 0.0002$) and spleen (T: $p = 0.0258$; M: $p = 0.0175$; G: $p = 0.0293$) of the fatty liver group. Consistently, only the ROS in hepatic M-MDSCs was inhibited by FATP2 blockade ($p = 0.0009$) (Fig. 7G and Fig. S9F). In the fatty liver group, CD36 in M-MDSCs was enhanced in the liver ($p = 0.0069$) and spleen ($p = 0.0233$). FATP2 inhibition could significantly reduce the CD36 levels in hepatic MDSCs (T: $p = 0.0347$; M: $p = 0.0175$; G: $p = 0.0429$) (Fig. S9G). FATP2 levels in liver tumour are lower than those in non-tumour liver tissues in the Gene Expression Profiling Interactive Analysis database, and the viability of tumour cells (Hepa1-6) was not obviously affected by lipofermata in our preliminary data. Therefore, these results excluded the effects of lipofermata on tumour cells. These data suggested that FATP2 blockade could reduce the tumour recurrence through suppressing the activation of NLRP3 inflammasome in MDSCs, which further inhibited the IL-17 production of CD4^{+} T cells.

Discussion

In the current study, we demonstrated that the inflammasome activation in MDSCs bridged the acute-phase steatotic liver graft injury and tumour recurrence. Our finding provided the new insight that fatty liver graft injury induced the immunosuppressive environment, which contributed to tumour recurrence after transplantation. Our study indicated the casual effect of inflammation induced by steatotic liver graft injury on tumour recurrence from the perspective of immunosuppressive environment formation post transplantation.

We first identified that arachidonic acid activated NLRP3 inflammasome in MDSCs post transplantation using fatty grafts. MDSCs represent a heterogeneous population of myeloid progenitor cells, whose immune function was influenced by lipid metabolism.[32] For example, the uptake of triacylglycerol substrates and subsequent lipolysis are essential for M2 activation.[33] The lipid accumulation in dendritic cells impairs their ability to process and present antigens in tumour.[34] The fatty acid uptake reprogrammes the neutrophils into G-MDSCs to promote tumour progression.[17] However, previous studies mainly focused on the role of lipid metabolism promoting the immunosuppressive phenotype of MDSCs.[15–17] Our current study first explored the mechanism for lipid metabolism on inflammasome activation in MDSCs. Arachidonic acid was firstly found to activate inflammasome in the current study, although other lipids have been reported, including palmitic acid.[35] The alteration of arachidonic acid metabolism was found by both GC-MS and RNA-seq analysis in our study, suggesting its critical role in steatotic liver graft injury. It was recently reported that arachidonic acid also induced the immunosuppressive activity acquisition of neutrophils.[17] The accumulation of arachidonic acids in hepatic MDSCs could enhance NLRP3 and FATP2 in both liver and splenic MDSCs. It indicated the significant impact of fatty acid metabolism alteration in hepatic MDSCs. The detailed alterations of lipid metabolism pathways are worthwhile for further independent studies.

The fatty liver graft injury was associated with impairment of hepatic microcirculation and dysfunction of mitochondria.[4,36–38] NLRP3 inflammasome activation was found to be associated with mitochondrial dysfunction in the present study. The hepatic IRI induced the mitophagy and permeability change of mitochondria.[6] Our recent study also demonstrated that a compromised AMPK–PGC1α axis exacerbated steatotic liver graft injury by dysregulating mitochondrial homoeostasis.[4] The dysfunction of mitochondria produced excessive ROS, resulting in the oxidative stress and cytotoxicity.[39] Mitochondrial DNA release was demonstrated as the link between palmitic acid stimulation and inflammasome activation.[35] The previous reports showed that succinate was a universal metabolic signature of ischaemia in transplanted organs (including the liver) and is responsible for mitochondrial ROS production during reperfusion at complex I although there are controversial findings in humans.[40,41] In the current study, ROS was increased in MDSCs by arachidonic acid uptake through FATP2 in the fatty graft post liver transplantation. We found that the complexes of mitochondria were impaired with reduced levels of ATP by arachidonic acid stimulation, but the effects could be rescued by FATP2 inhibition. Our finding might contribute to the mechanism of fatty acid metabolism on mitochondrial dysfunction in MDSCs.

FATP2, as the most critical member of the FATP family in the liver, is identified as a major contributor to peroxisomal very-long-chain acyl-CoA synthetase and hepatic fatty acid uptake.[42] In our study, FATP2 was found as the transporter that mediated arachidonic acid uptake. The enhanced CD36, the classical lipid uptake receptor, was also reduced by FATP2 blockade, which demonstrated its critical role in lipid uptake. Interestingly, FATP2 on M-MDSCs was obviously higher than FATP2 on G-MDSCs, which might explain that NLRP3 activation in M-MDSCs was more sensitive by FATP2 inhibition. Moreover, the hepatic M-MDSCs were distinctly increased in fatty liver IRI. The previous findings reported that FATP2 blockade could abrogate the activity of G-MDSCs and substantially delayed tumour progression.[17] The mechanisms of FATP2 overexpression on M-MDSCs found in the current study need to be further explored. Based on our findings, the treatment of FATP2 inhibitors before and after

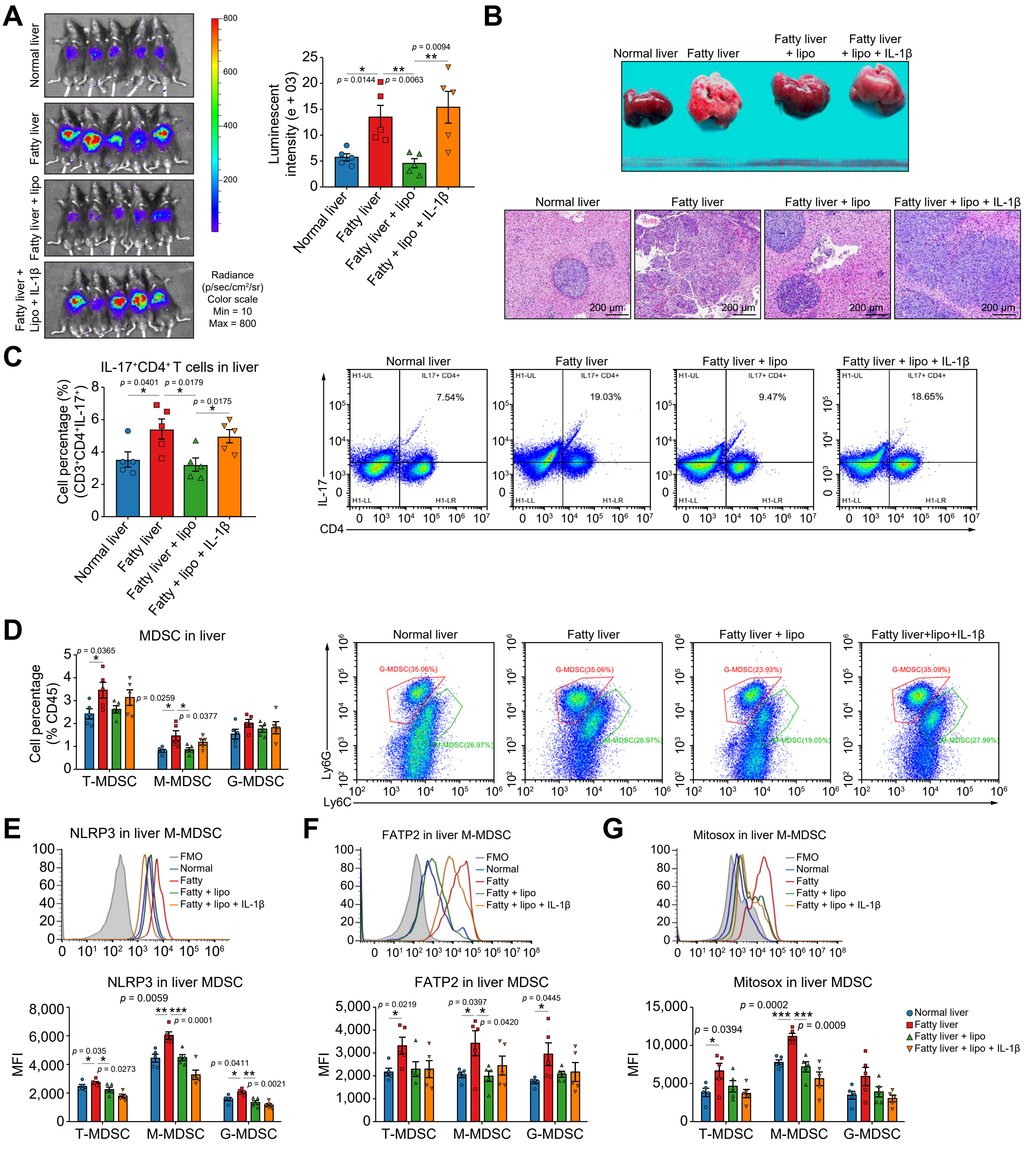

Fig. 7. Targeting FATP2 inhibited liver tumour recurrence in mice. (A) The tumour size was increased in the fatty liver, and decreased and restored by Lipo and IL-1β recombinant protein injection, respectively. (B) FATP2 blockade inhibited the increased tumour size in the fatty liver, whereas IL-1β injection offset the inhibition effects in the mouse tumour recurrence model. (C) IL-17$^+$ CD4$^+$ T cells were accumulated in the fatty liver, and reduced and raised by Lipo and IL-1β, respectively. (D) The population of liver M-MDSCs was increased in the fatty liver and decreased by FATP2 blockade. (E) NLRP3 in liver MDSCs was upregulated in the fatty liver and reduced by FATP2 inhibition. (F) FATP2 in hepatic MDSCs was enhanced in the fatty liver, whereas Lipo inhibited its levels in M-MDSCs. (G) The ROS in liver M-MDSCs was increased in the fatty liver and diminished by FATP2 inhibition. (A–G) n = 5/group. Error bars indicate SEM; *p <0.05, **p <0.01, ***p <0.001. FATP2, fatty acid transport protein 2; FMO, fluorescence minus one; G-MDSC, granulocytic MDSC; Lipo, lipofermata; M-MDSC, monocytic MDSC; MDSC, myeloid-derived suppressor cell; MFI, mean fluorescence intensity; NLRP3, nucleotide-binding oligomerisation domain-like receptor family pyrin domain containing 3; ROS, reactive oxygen species; T-MDSC, total MDSC.

transplantation for the recipients decreased the harmful effects of aberrant fatty acid metabolism on liver graft immune environment during acute injury. We previously also found that inhibiting lipocalin-2 could attenuate fatty liver graft injury.[43] Our recent study showed that metformin could alleviate steatotic liver graft injury by restoring mitochondrial function through AMPK reactivation.[4] Probably, the combination therapy or personalised treatments targeting at FATP2 and lipocalin-2, together with metformin, might be the solutions to attenuate steatotic graft injury and subsequently reduce cancer recurrence after transplantation. Moreover, combine machine perfusion and FATP2 inhibitor might be the direction to the clinical practice owing to the observed effect of hypothermic machine perfusion on HCC recurrence in livers from donation after circulatory death and the overall protection from complications and graft loss in recipients of steatotic livers from deceased donors.[44,45]

As more primitive cells, more infiltrated M-MDSCs with higher FATP2 might have profound impact on the immune microenvironment, such as T cells. In the current study, the inflammasome activation by arachidonic acid produced more bioactive IL-1β, which skewed the naive $CD4^+$ T cells differentiated into Th17 cells. This was consistent with the report that the chemotherapy triggered inflammasome activation in MDSCs, which induced the IL-17 secretion of $CD4^+$ T cells.[26] Th17 cells not only can switch to a Th1 phenotype and exert antitumour activities but also are capable of expressing a regulatory T cell phenotype, which can perform protumour and antitumour activities, depending on the context of the immune response.[46,47] In HCC, accumulation of intratumour Th17 cells promote tumour progression through fostering angiogenesis, and associated with poor survival in patients.[31] The mechanism of Th17 promoting liver tumour progression is not well understood. One study demonstrated that IL-17 has a direct effect over HCC with the induction of IL-6/Janus kinase 2 (JAK2)/signal transducer and activator of transcription 3 (STAT3) by activating the protein kinase B (AKT) pathway.[48] The STAT3 signalling promotes tumour growth through the regulation of pro-angiogenic genes,[49] which is echoed by our findings that angiogenesis was significantly enhanced in fatty grafts post liver transplantation. Our previous data showed that the counts of MDSCs were obviously increased after hepatic IRI, and the current study demonstrated that fatty liver grafts recruited more M-MDSCs. Therefore, accumulated MDSCs and the higher inflammasome activation co-ordinately induced more IL-17 production of naive $CD4^+$ T cells, contributing to the impairment of tumour surveillance. The recruitment of M-MDSCs to fatty liver grafts might be associated with more bioactive IL-1β release as a result of the inflammasome activation.

Taken together, arachidonic acid activated NLRP3 inflammasome in MDSCs through FATP2 during fatty liver graft injury, which led to more IL-17 secretion of $CD4^+$ T cells and promoted tumour recurrence post transplantation through our integrated study with clinical analyses, animal models, and *in vitro* experiments. Targeting FATP2 represents the novel therapeutic strategies to reduce tumour recurrence post liver transplantation and to potentially expand the donor pool.

Abbreviations

AA, arachidonic acid; ATP5A1, ATP synthase alpha-subunit; CXCL1, C–X–C motif chemokine ligand 1; DHR, dihydrorhodamine 123; DRP1, dynamin-related protein 1; FATP2, fatty acid transport protein 2; FMO, fluorescence minus one; G-MDSC, granulocytic MDSC; GC-MS, gas chromatography–mass spectrometry; HCC, hepatocellular carcinoma; IFN-γ, interferon-γ; IRI, ischaemia/reperfusion injury; JAK2, Janus kinase 2; KC, keratinocyte chemoattractant; KEGG, Kyoto Encyclopedia of Genes and Genomes; Lipo, lipofermata; M-MDSC, monocytic MDSC; MCP-1, monocyte chemoattractant protein 1; MDSC, myeloid-derived suppressor cell; MFI, mean fluorescence intensity; MT-CO1, mitochondrially encoded cytochrome C oxidase I; NC, negative control; NDUFB8, NADH dehydrogenase (ubiquinone) 1 beta subcomplex subunit 8; NLRP3, nucleotide-binding oligomerisation domain-like receptor family pyrin domain containing 3; PBMC, peripheral blood mononuclear cell; PHB1, prohibitin 1; RAP1, telomere-independent repressor activator protein 1; RNA-seq, RNA sequencing; SDHB, succinate dehydrogenase B; SOD1, superoxide dismutase 1; STAT3, signal transducer and activator of transcription 3; T-MDSC, total MDSC; Th17, T helper 17; TLR4, Toll-like receptor 4; UQCRC2, ubiquinol–cytochrome C reductase core protein 2; VDAC, voltage-dependent anion channel; AKT, protein kinase B.

Financial support

This work was supported by the General Research Funding (GRF: 17106921&17124219), Theme-based Research Scheme (TRS: T12-703/19R), and Collaborative Research Funding (C7026-18 GF&C7021-21G) from the Research Grant Council, Hong Kong. This work was also supported by the National Science Foundation of China (NSFC) grants (82203794 and 82072757).

Conflicts of interest

All authors have no conflicts of interest to be declared.

Please refer to the accompanying ICMJE disclosure forms for further details.

Authors' contributions

Designed the study, performed the experiments and interpretation of data, and wrote/revised the manuscript: HL, KM. Obtained fundings and supervised the study: KM, CL. Was involved in technical support: HL, WY, LP, JL, XL, KN, QZ, WQ, YZ, TD, ZW, JZ. Had final approval of the submitted version: all authors.

Data availability statement

The data generated in this study are available upon request from the corresponding author. The RNA-seq data have been submitted and are available through the NCBI's Gene Expression Omnibus (GEO GSE204919).

Supplementary data

Supplementary data to this article can be found online at https://doi.org/10.1016/j.jhepr.2023.100895.

References

Author names in bold designate shared co-first authorship

[1] Younossi Z, Tacke F, Arrese M, Chander Sharma B, Mostafa I, Bugianesi E, et al. Global perspectives on nonalcoholic fatty liver disease and nonalcoholic steatohepatitis. Hepatology 2019;69:2672–2682.

[2] **Linares I**, **Hamar M**, Selzner N, Selzner M. Steatosis in liver transplantation: current limitations and future strategies. Transplantation 2019;103:78–90.

[3] Jackson KR, Long J, Philosophe B, Garonzik-Wang J. Liver transplantation using steatotic grafts. Clin Liver Dis 2019;14:191–195.

[4] Liu J, Pang L, Ng KTP, Chiu TLS, Liu H, Liu X, et al. Compromised AMPK-PGC1α axis exacerbated steatotic graft injury by dysregulating

mitochondrial homeostasis in living donor liver transplantation. Ann Surg 2022;276:e483–e492.
[5] Orci L, Lacotte S, Oldani G, Slits F, De Vito C, Crowe L, et al. Effect of ischaemic preconditioning on recurrence of hepatocellular carcinoma in an experimental model of liver steatosis. Br J Surg 2016;103:417–426.
[6] Liu H, Man K. New insights in mechanisms and therapeutics for short- and long-term impacts of hepatic ischemia reperfusion injury post liver transplantation. Int J Mol Sci 2021;22:8210.
[7] Van Der Bilt JD, Kranenburg O, Nijkamp MW, Smakman N, Veenendaal LM, Te Velde EA, et al. Ischemia/reperfusion accelerates the outgrowth of hepatic micrometastases in a highly standardized murine model. Hepatology 2005;42:165–175.
[8] **Li CX**, **Ling CC**, Shao Y, Xu A, Li XC, Ng KT-P, et al. CXCL10/CXCR3 signaling mobilized-regulatory T cells promote liver tumor recurrence after transplantation. J Hepatol 2016;65:944–952.
[9] Gabrilovich DI, Nagaraj S. Myeloid-derived suppressor cells as regulators of the immune system. Nat Rev Immunol 2009;9:162–174.
[10] Liu C, Yu S, Kappes J, Wang J, Grizzle WE, Zinn KR, et al. Expansion of spleen myeloid suppressor cells represses NK cell cytotoxicity in tumor-bearing host. Blood 2007;109:4336–4342.
[11] Huang B, Pan P-Y, Li Q, Sato AI, Levy DE, Bromberg J, et al. Gr-1$^+$ CD115$^+$ immature myeloid suppressor cells mediate the development of tumor-induced T regulatory cells and T-cell anergy in tumor-bearing host. Cancer Res 2006;66:1123–1131.
[12] Sinha P, Clements VK, Bunt SK, Albelda SM, Ostrand-Rosenberg S. Cross-talk between myeloid-derived suppressor cells and macrophages subverts tumor immunity toward a type 2 response. J Immunol 2007;179:977–983.
[13] Condamine T, Ramachandran I, Youn J-I, Gabrilovich DI. Regulation of tumor metastasis by myeloid-derived suppressor cells. Annu Rev Med 2015;66:97–110.
[14] **Hossain F**, **Al-Khami AA**, Wyczechowska D, Hernandez C, Zheng L, Reiss K, et al. Inhibition of fatty acid oxidation modulates immunosuppressive functions of myeloid-derived suppressor cells and enhances cancer therapies. Cancer Immunol Res 2015;3:1236–1247.
[15] Al-Khami AA, Zheng L, Del Valle L, Hossain F, Wyczechowska D, Zabaleta J, et al. Exogenous lipid uptake induces metabolic and functional reprogramming of tumor-associated myeloid-derived suppressor cells. Oncoimmunology 2017;6:e1344804.
[16] Condamine T, Dominguez GA, Youn J-I, Kossenkov AV, Mony S, Alicea-Torres K, et al. Lectin-type oxidized LDL receptor-1 distinguishes population of human polymorphonuclear myeloid-derived suppressor cells in cancer patients. Sci Immunol 2016;1:aaf8943.
[17] Veglia F, Tyurin VA, Blasi M, De Leo A, Kossenkov AV, Donthireddy L, et al. Fatty acid transport protein 2 reprograms neutrophils in cancer. Nature 2019;569:73–78.
[18] Liu H, Ling CC, Yeung WHO, Pang L, Liu J, Zhou J, et al. Monocytic MDSC mobilization promotes tumor recurrence after liver transplantation via CXCL10/TLR4/MMP14 signaling. Cell Death Dis 2021;12:489.
[19] Martinon F, Burns K, Tschopp J. The inflammasome: a molecular platform triggering activation of inflammatory caspases and processing of proIL-β. Mol Cell 2002;10:417–426.
[20] Szabo G, Petrasek J. Inflammasome activation and function in liver disease. Nat Rev Gastroenterol Hepatol 2015;12:387–400.
[21] Csak T, Ganz M, Pespisa J, Kodys K, Dolganiuc A, Szabo G. Fatty acid and endotoxin activate inflammasomes in mouse hepatocytes that release danger signals to stimulate immune cells. Hepatology 2011;54:133–144.
[22] **Mridha AR**, **Wree A**, Robertson AA, Yeh MM, Johnson CD, Van Rooyen DM, et al. NLRP3 inflammasome blockade reduces liver inflammation and fibrosis in experimental NASH in mice. J Hepatol 2017;66:1037–1046.
[23] Liu H, Lo CM, Yeung OWH, Li CX, Liu XB, Qi X, et al. NLRP3 inflammasome induced liver graft injury through activation of telomere-independent RAP1/KC axis. J Pathol 2017;242:284–296.
[24] Zhu P, Duan L, Chen J, Xiong A, Xu Q, Zhang H, et al. Gene silencing of NALP3 protects against liver ischemia–reperfusion injury in mice. Hum Gene Ther 2011;22:853–864.
[25] **Wei Q**, **Mu K**, Li T, Zhang Y, Yang Z, Jia X, et al. Deregulation of the NLRP3 inflammasome in hepatic parenchymal cells during liver cancer progression. Lab Invest 2014;94:52–62.
[26] **Bruchard M**, **Mignot G**, Derangère V, Chalmin F, Chevriaux A, Végran F, et al. Chemotherapy-triggered cathepsin B release in myeloid-derived suppressor cells activates the Nlrp3 inflammasome and promotes tumor growth. Nat Med 2013;19:57–64.
[27] Man K, Lo C-M, Ng IO-L, Wong Y-C, Qin L-F, Fan S-T, et al. Liver transplantation in rats using small-for-size grafts: a study of hemodynamic and morphological changes. Arch Surg 2001;136:280–285.
[28] Cheng Q, Ng K, Fan S, Lim Z, Guo D, Liu X, et al. Distinct mechanism of small-for-size fatty liver graft injury – Wnt4 signaling activates hepatic stellate cells. Am J Transpl 2010;10:1178–1188.
[29] Ling C-C, Ng KT, Shao Y, Geng W, Xiao J-W, Liu H, et al. Post-transplant endothelial progenitor cell mobilization via CXCL10/CXCR3 signaling promotes liver tumor growth. J Hepatol 2014;60:103–109.
[30] Pang L, Yeung OW, Ng KT, Liu H, Zhu J, Liu J, et al. Postoperative plasmacytoid dendritic cells secrete IFNα to promote recruitment of myeloid-derived suppressor cells and drive hepatocellular carcinoma recurrence. Cancer Res 2022;82:4206–4218.
[31] **Zhang J-P**, **Yan J**, Xu J, Pang X-H, Chen M-S, Li L, et al. Increased intratumoral IL-17-producing cells correlate with poor survival in hepatocellular carcinoma patients. J Hepatol 2009;50:980–989.
[32] Bronte V, Brandau S, Chen S-H, Colombo MP, Frey AB, Greten TF, et al. Recommendations for myeloid-derived suppressor cell nomenclature and characterization standards. Nat Commun 2016;7:12150.
[33] Huang SC-C, Everts B, Ivanova Y, O'Sullivan D, Nascimento M, Smith AM, et al. Cell-intrinsic lysosomal lipolysis is essential for alternative activation of macrophages. Nat Immunol 2014;15:846–855.
[34] **Herber DL**, **Cao W**, Nefedova Y, Novitskiy SV, Nagaraj S, Tyurin VA, et al. Lipid accumulation and dendritic cell dysfunction in cancer. Nat Med 2010;16:880–886.
[35] **Pan J**, **Ou Z**, Cai C, Li P, Gong J, Ruan XZ, et al. Fatty acid activates NLRP3 inflammasomes in mouse Kupffer cells through mitochondrial DNA release. Cell Immunol 2018;332:111–120.
[36] Ijaz S, Yang W, Winslet MC, Seifalian AM. Impairment of hepatic microcirculation in fatty liver. Microcirculation 2003;10:447–456.
[37] Fukumori T, Ohkohchi N, Tsukamoto S, Satomi S. Why is fatty liver unsuitable for transplantation? Deterioration of mitochondrial ATP synthesis and sinusoidal structure during cold preservation of a liver with steatosis. Transpl Proc 1997;29:4125.
[38] Chu MJ, Hickey AJ, Phillips AR, Bartlett AS. The impact of hepatic steatosis on hepatic ischemia–reperfusion injury in experimental studies: a systematic review. Biomed Res Int 2013;2013:192029.
[39] Videla LA, Fernández V. Biochemical aspects of cellular oxidative stress. Arch Biol Med Exp 1988;21:85–92.
[40] **Chouchani ET**, **Pell VR**, Gaude E, Aksentijević D, Sundier SY, Robb EL, et al. Ischaemic accumulation of succinate controls reperfusion injury through mitochondrial ROS. Nature 2014;515:431–435.
[41] Wijermars L, Schaapherder A, Kostidis S, Wüst R, Lindeman J. Succinate accumulation and ischemia–reperfusion injury: of mice but not men, a study in renal ischemia–reperfusion. Am J Transpl 2016;16:2741–2746.
[42] **Falcon A**, **Doege H**, Fluitt A, Tsang B, Watson N, Kay MA, et al. FATP2 is a hepatic fatty acid transporter and peroxisomal very long-chain acyl-CoA synthetase. Am J Physiol Endocrinol Metab 2010;299:E384–E393.
[43] **Cheng Q**, **Ng KT**, Xu A, Li C-X, Liu X-B, Guo D-Y, et al. The roles of lipocalin-2 in small-for-size fatty liver graft injury. Ann Surg 2014;260:1062–1072.
[44] **Mueller M**, **Kalisvaart M**, **Joanne OR**, Shetty S, Parente A, Muller X, et al. Hypothermic oxygenated liver perfusion (HOPE) prevents tumor recurrence in liver transplantation from donation after circulatory death. Ann Surg 2020;272:759–765.
[45] **Kron P**, **Schlegel A**, Mancina L, Clavien P-A, Dutkowski P. Hypothermic oxygenated perfusion (HOPE) for fatty liver grafts in rats and humans. J Hepatol 2018;68:82–91.
[46] **Bending D**, **De La Peña H**, Veldhoen M, Phillips JM, Uyttenhove C, Stockinger B, et al. Highly purified Th17 cells from BDC2.5NOD mice convert into Th1-like cells in NOD/SCID recipient mice. J Clin Invest 2009;119:565–572.
[47] Ye J, Su X, Hsueh EC, Zhang Y, Koenig JM, Hoft DF, et al. Human tumor-infiltrating Th17 cells have the capacity to differentiate into IFN-γ^+ and FOXP3$^+$ T cells with potent suppressive function. Eur J Immunol 2011;41:936–951.
[48] **Gu F-M**, **Li Q-L**, **Gao Q**, Jiang J-H, Zhu K, Huang X-Y, et al. IL-17 induces AKT-dependent IL-6/JAK2/STAT3 activation and tumor progression in hepatocellular carcinoma. Mol Cancer 2011;10:150.
[49] Yu H, Kortylewski M, Pardoll D. Crosstalk between cancer and immune cells: role of STAT3 in the tumour microenvironment. Nat Rev Immunol 2007;7:41–51.

Journal of Hepatology, Volume *5*

Supplemental information

Arachidonic acid activates NLRP3 inflammasome in MDSCs via FATP2 to promote post-transplant tumour recurrence in steatotic liver grafts

Hui Liu, Wai Ho Oscar Yeung, Li Pang, Jiang Liu, Xiao Bing Liu, Kevin Tak Pan Ng, Qingmei Zhang, Wen Qi Qiu, Yueqin Zhu, Tao Ding, Zhe Wang, Ji Ye Zhu, Chung Mau Lo, and Kwan Man

Arachidonic acid activates NLRP3 inflammasome in MDSCs via FATP2 to promote post-transplant tumor recurrence in steatotic liver grafts

Hui Liu, Wai Ho Oscar Yeung, Li Pang, Jiang Liu, Xiao Bing Liu, Kevin Tak Pan Ng, Qingmei Zhang, Wen Qi Qiu, Yueqin Zhu, Tao Ding, Zhe Wang, Ji Ye Zhu, Chung Mau Lo, Kwan Man

Table of contents

Supplementary figures……………………………………............……………………………….2

Supplementary tables………………………………………………...........……………….… 11

Supplementary figure legends…………………………………………….........……….…..14

Supplementary materials and methods…………………………………………........……19

Supplementary references...22

Supplementary figures

Fig. S1

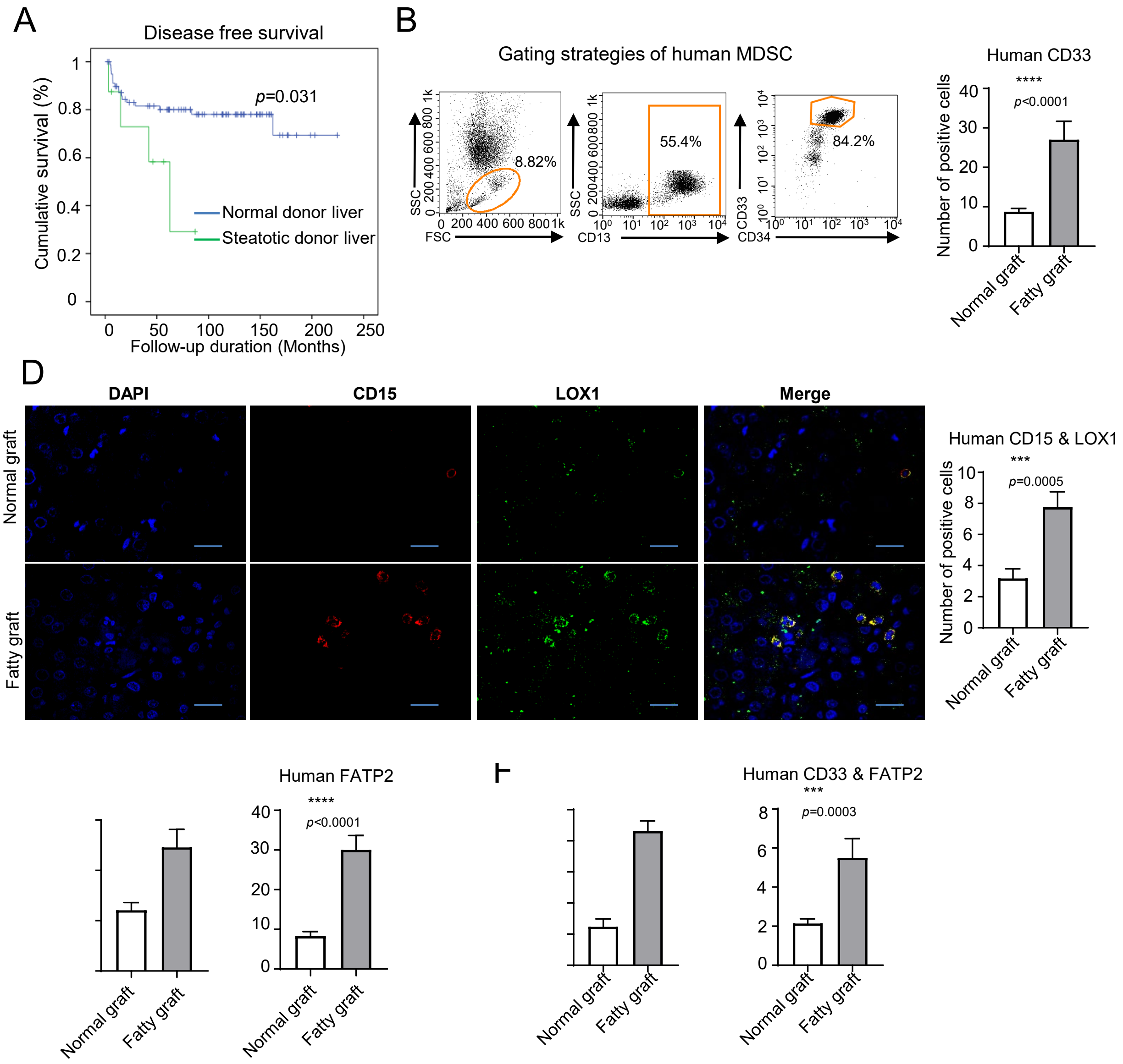

Fig. S1 The tumor recurrent rate was higher in HCC patients underwent liver transplantation using steatotic donor accompanied with the increase of MDSCs and NLRP3/FATP2 levels. A The tumor recurrence free survival was relatively poor in HCC patients after liver transplantation using steatotic donor (n=88). **B** The gating strategies of human MDSCs (CD33$^+$CD13$^+$CD34$^+$) by flow cytometry (n=45). **C** The number of MDSCs (CD33$^+$) were significantly accumulated in steatotic grafts by immunohistochemistry (n=23). **D** More MDSCs were infiltrated in fatty grafts by co-staining of CD15 & LOX1 (n=23). Scale bars: 20μm. **E** The increased numbers of NLRP3 and FATP2 positive cells in fatty grafts by immunohistochemistry (n=23). **F** More CD33$^+$NLRP3$^+$ and CD33$^+$FATP2$^+$ cells were infiltrated in fatty grafts post liver transplantation by co-staining (n=23). Error bars indicate Standard Error of Mean; *$p<0.05$, **$p<0.01$, ***$p<0.001$, ****$p<0.0001$. MDSC, myeloid-derived suppressor cell; NLRP3: nucleotide-binding oligomerization domain-like receptor family pyrin domain containing 3; FATP2: fatty acid transport protein 2.

Fig. S2

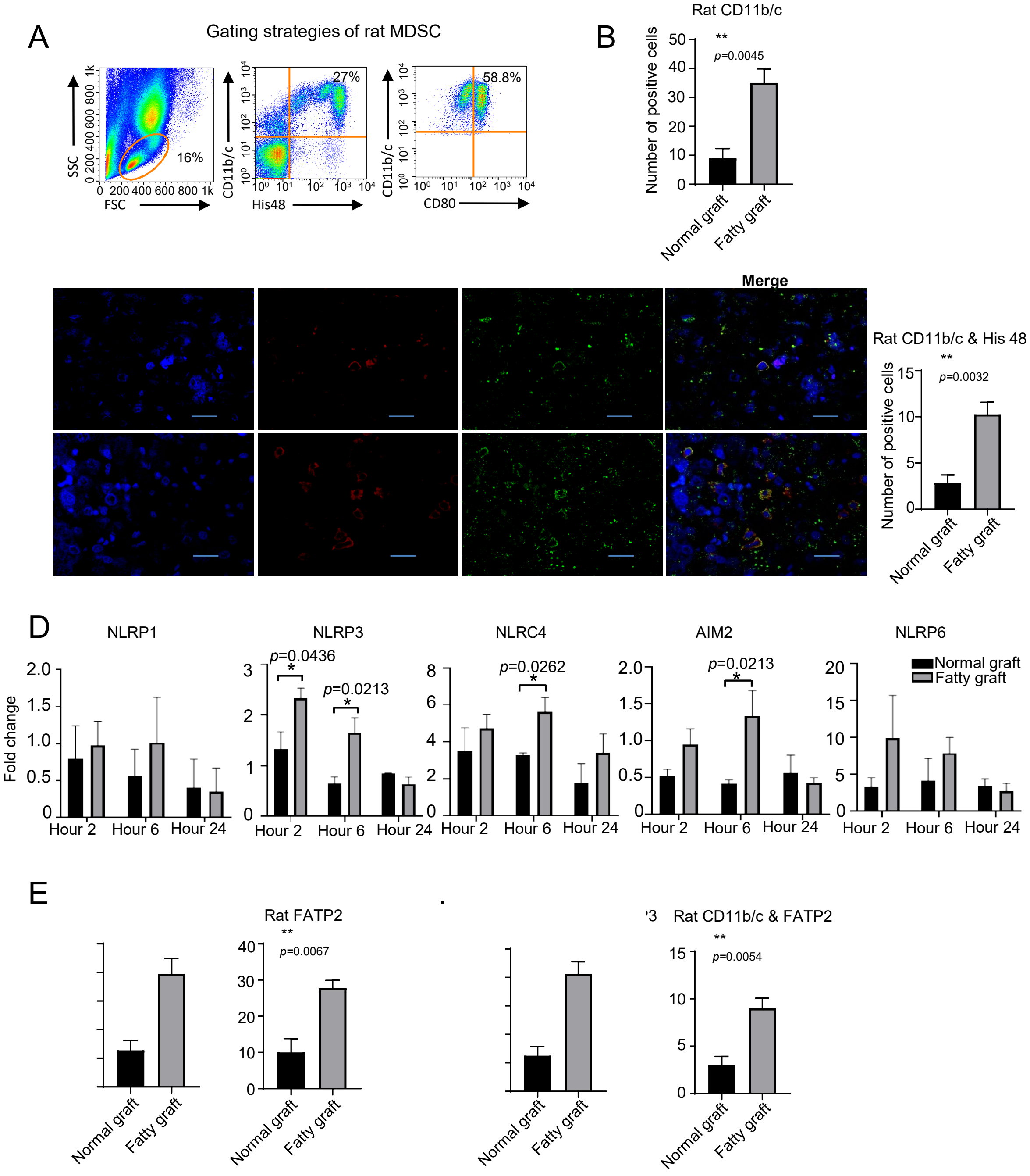

Fig. S2 The levels of NLRP3 and FATP2 were higher with accumulation of MDSCs in fatty liver grafts of rat model. A The gating strategies of rat MDSCs ($CD11b/c^+CD80^+His48^+$) by flow cytometry. **B** The number of $CD11b/c^+$ (MDSC marker) cells were obviously increased in rat fatty liver grafts by immunohistochemistry. **C** Accumulated MDSCs in fatty grafts by co-staining of CD11b/c & His48. Scale bars: 20μm. **D** NLRP3 was upreglated in fatty grafts through the inflammsome (NLRP1, NLRP3, NLRC4, AIM2, NLRP6) mRNA screen of rat liver transplantation model. **E** NLRP3 and FATP2 positive cells were more in fatty liver grafts by immunohistochemistry. **F** The immunofluorescent co-staining showed increased $CD11b/c^+NLRP3^+$ and $CD11b/c^+FATP2^+$ cells in fatty grafts post liver transplantation. A-F: n=4/group; Error bars indicate Standard Error of Mean; **p<0.05, **p<0.01, ***p<0.001, ****p<0.0001.* MDSC, myeloid-derived suppressor cell; NLRP: nucleotide-binding oligomerization domain-like receptor family pyrin domain containing; FATP2: fatty acid transport protein 2; NLRC4: NLR Family CARD Domain Containing 4; AIM2: Absent In Melanoma 2.

Fig. S3

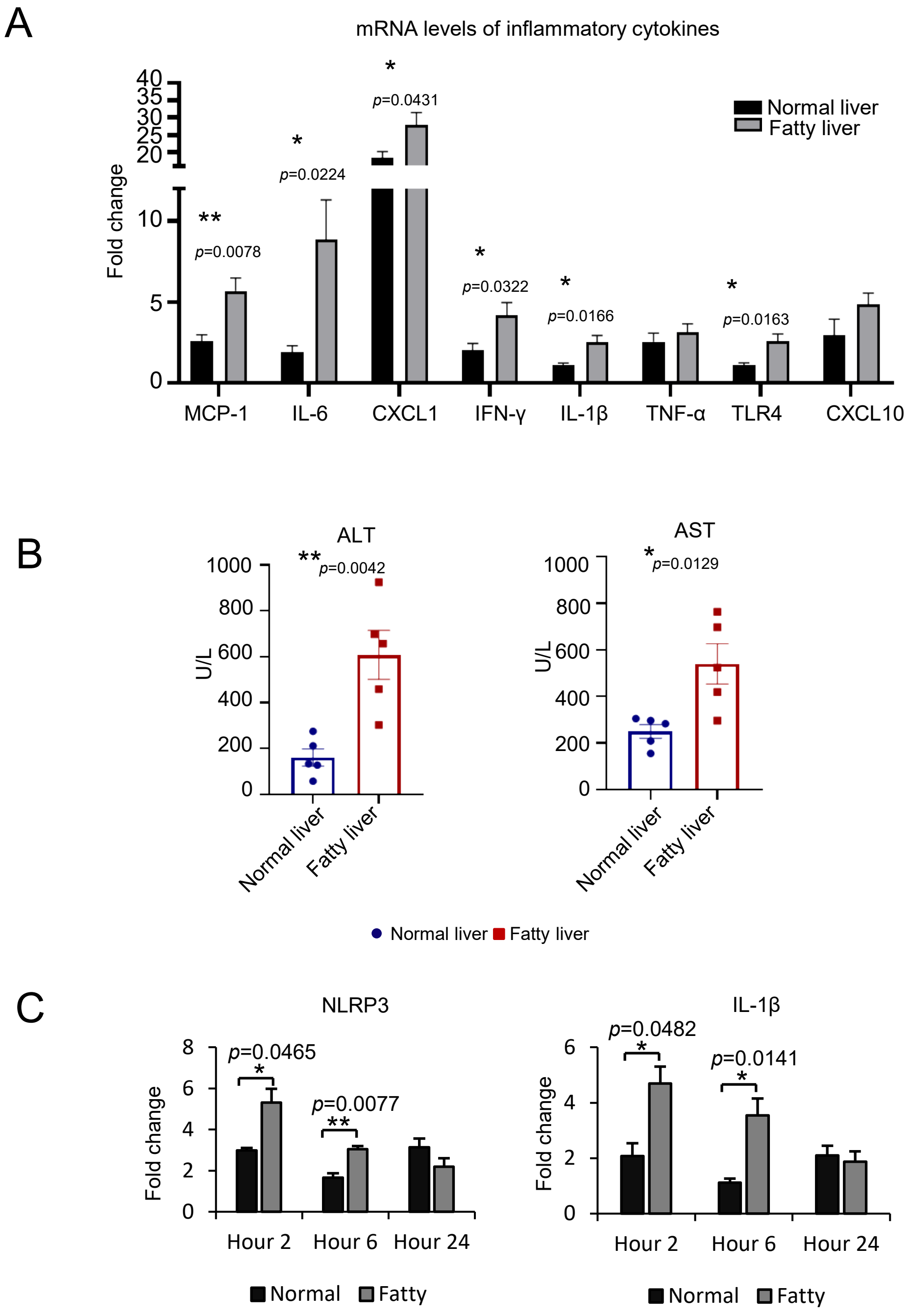

Fig. S3 The severer inflammatory injury and poorer liver function with enhanced NLRP3 and IL-1β expressions in mice post fatty liver ischemia/reperfusion injury (IRI). A The inflammatory markers, included MCP-1, IL-6, CXCL1, IFN-γ, IL-1β and TLR4, were significantly upregulated post fatty liver IRI. **B** The liver function was poorer with increased alanine transaminase (ALT) and aspartate aminotransferase (AST) in fatty liver after IRI. **C** The mRNA levels of NLRP3 and IL-1β were increased in fatty liver post IRI in mice**.** A-C: n=5/group; Error bars indicate Standard Error of Mean; **p<0.05, **p<0.01.* NLRP3: nucleotide-binding oligomerization domain-like receptor family pyrin domain containing 3; IRI, ischemia/reperfusion injury; MCP-1: Monocyte Chemoattractant Protein 1; IL-6: Interleukin-6; CXCL1: C-X-C Motif chemokine ligand 1; IFN-γ: Interferon-γ; TNF-α: tumor necrosis factor α; TLR4: Toll Like Receptor 4; ALT: alanine transaminase; AST, aspartate aminotransferase.

Fig. S4

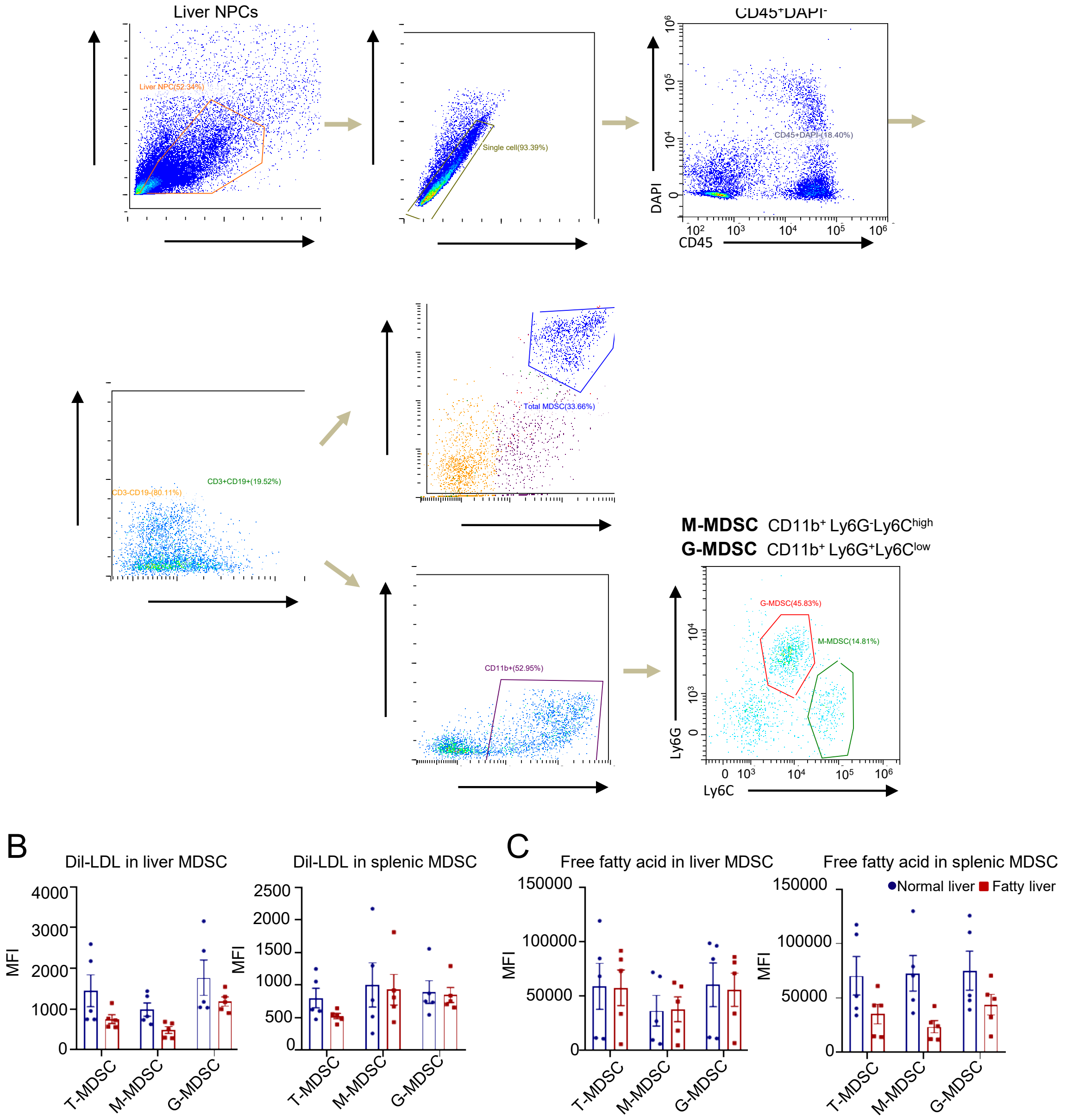

Fig. S4 No obvious change of Dil-LDL and free fatty acid in MDSCs of mice post fatty liver IRI. A The gating strategies of Total MDSC (T-MDSC, CD11b$^+$Gr1$^+$), Granulocytic MDSC (G-MDSC, CD11b$^+$ Ly6G$^+$Ly6C^{low}) and Monocytic MDSC (M-MDSC, CD11b$^+$ Ly6G$^-$Ly6C^{high}) in mice. **B** Dil-LDL in MDSCs was not significantly changed in mice after fatty liver IRI. **C** Free fatty acids were not altered obviously post fatty liver IRI. A-C: n=5/group; Error bars indicate Standard Error of Mean; *$*p<0.05$, **$p<0.01$.* IRI, ischemia/reperfusion injury; MDSC, myeloid-derived suppressor cell; LDL: low density lipoprotein; T-MDSC: total-MDSC; M-MDSC: monocytic-MDSC; G-MDSC: granulocytic-MDSC.

Fig. S5

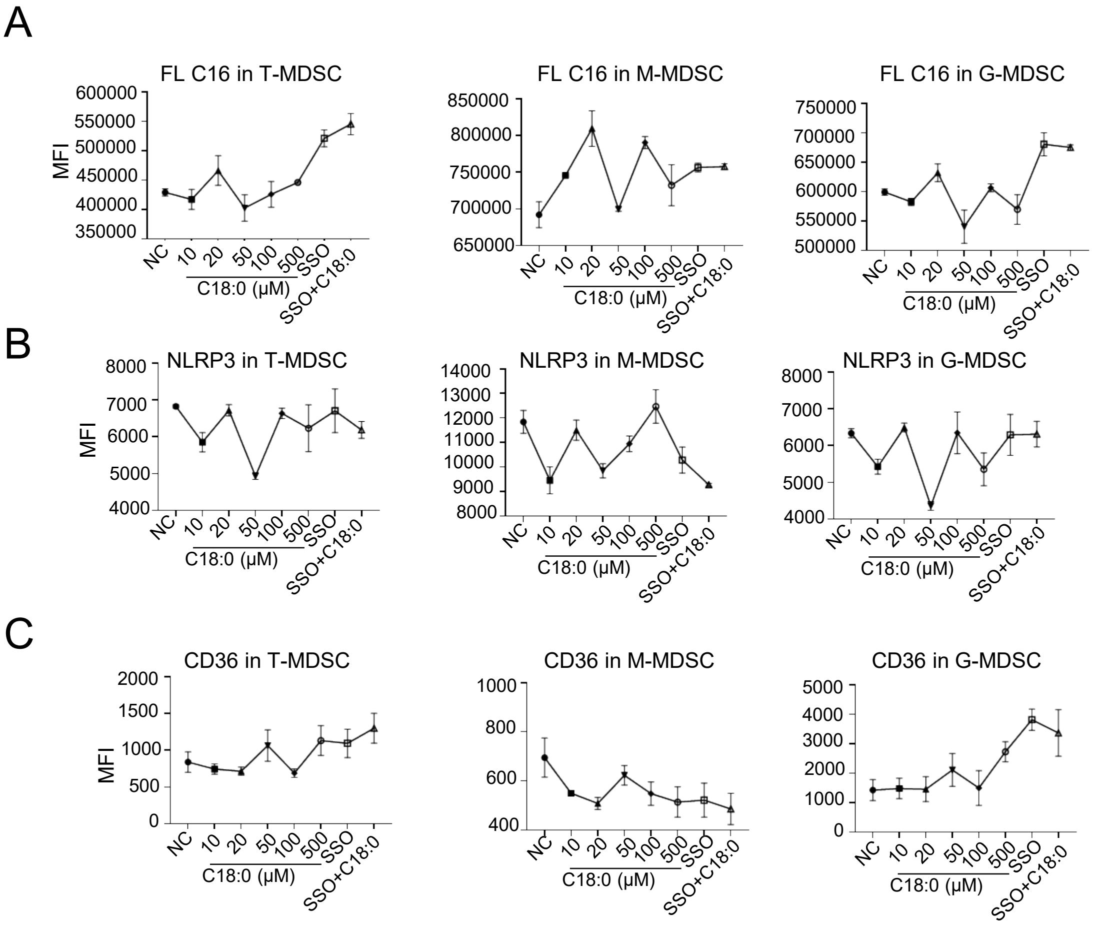

Fig. S5 NLRP3 in primary MDSCs was not changed by C18:0 stimulation. A FL C16 in primary MDSCs was not obviously altered by C18:0 or SSO (CD36 inhibitor) treatment. **B** C18:0 or SSO could not activate or inhibit NLRP3 in MDSCs, respectively. **C** CD36 in MDSCs was not significantly changed by C18:0 or SSO treatment. Error bars indicate Standard Error of Mean; NLRP3: nucleotide-binding oligomerization domain-like receptor family pyrin domain containing 3; MDSC, myeloid-derived suppressor cell; SSO: Sulfosuccinimidyl oleate sodium; T-MDSC: total-MDSC; M-MDSC: monocytic-MDSC; G-MDSC: granulocytic-MDSC.

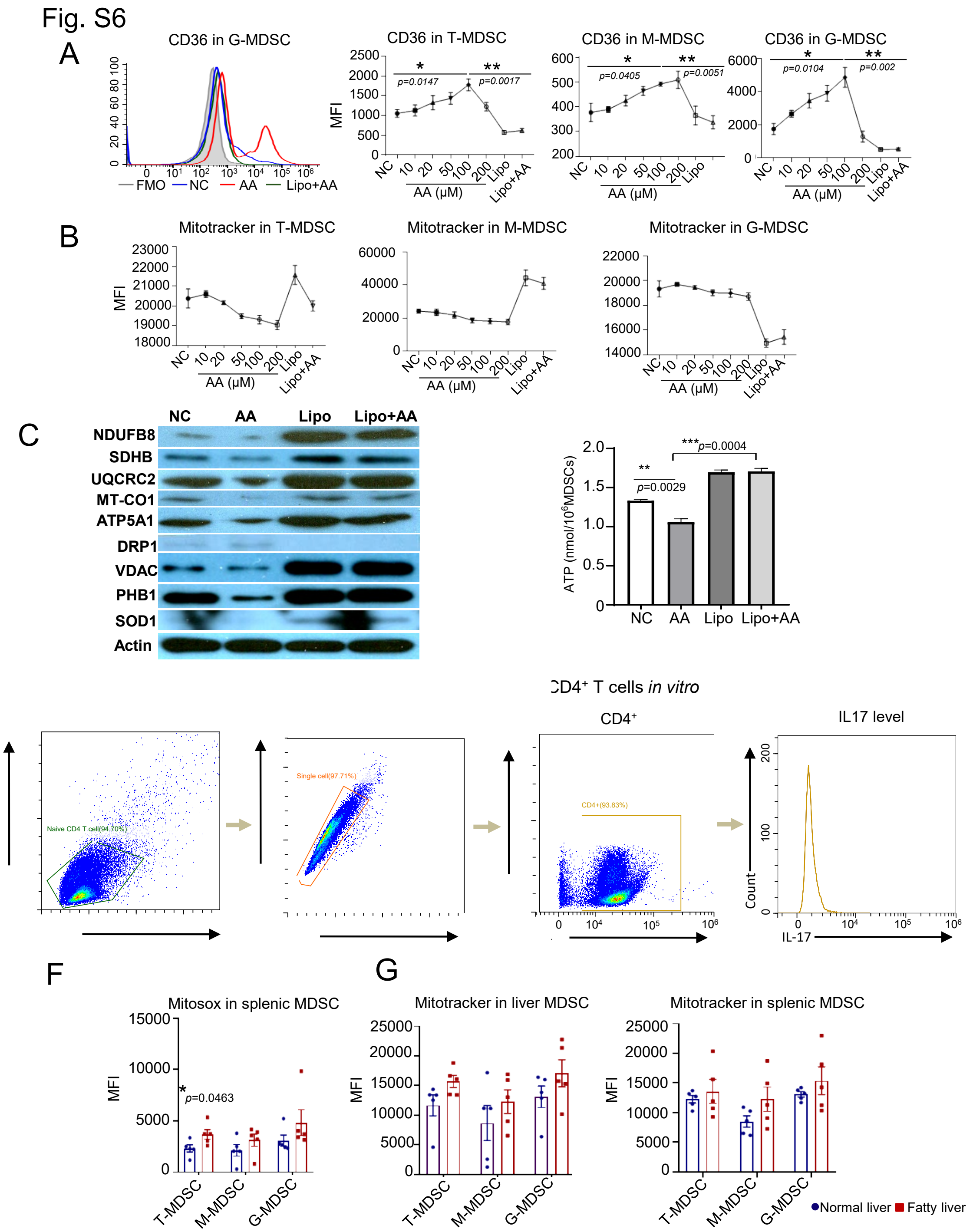

Fig. S6 The major proteins of mitochondria and ATPs were inhibited/enhanced by arachidonic acid or FATP2 blockade while mitochondrial counts were not changed obviously. A CD36 in primary MDSCs was upregulated and decreased by arachidonic acid (AA) and lipofermata (Lipo, FATP2 inhibitor), respectively. **B** The counts of mitochondria were not obviously changed by AA or lipofermata treatment. **C** The major mitochondrial protein levels, including NDUFB8 (complex I), SDHB (complex II), UQCRC2 (complex III), MT-CO1 (complex IV), ATP5A1 (complex V), VDAC, PHB1 and SOD1 in MDSCs were obviously decreased by AA but increased through the inhibition of FATP2. DRP1, the damage marker of mitochondria, was increased by AA but reduced by FATP2 inhibition. **D** The adenosine triphosphates (ATPs) produced by MDSCs were decreased by AA but enhanced by FATP2 blocking. **E** The gating strategies of IL-17 levels in $CD4^+$ T cells *in vitro*. **F** The mitosox was only increased in splenic total MDSCs. **G** The counts of mitochondria were not significantly altered post fatty liver IRI. F-G: n=5/group; Error bars indicate Standard Error of Mean; **p<0.05, **p<0.01, ***p<0.001, ****p<0.0001*. NC: negative control, the primary MDSCs without stimulation; FMO: fluorescence minus one; AA: arachidonic acid; Lipo: lipofermata; MDSC, myeloid-derived suppressor cell; T-MDSC: total-MDSC; M-MDSC: monocytic-MDSC; G-MDSC: granulocytic-MDSC; NDUFB8: NADH dehydrogenase (ubiquinone) 1 beta subcomplex subunit 8; SDHB: succinate dehydrogenase B; UQCRC2: ubiquinol-cytochrome C reductase core protein 2; MT-CO1: mitochondrially encoded cytochrome C oxidase I; ATP5A1: ATP synthase alpha-subunit; DRP1: dynamin-related protein 1; VDAC: voltage-dependent anion channel; PHB1: prohibitin 1; SOD1: superoxide dismutase 1; ATPs: adenosine triphosphates.

Fig. S7

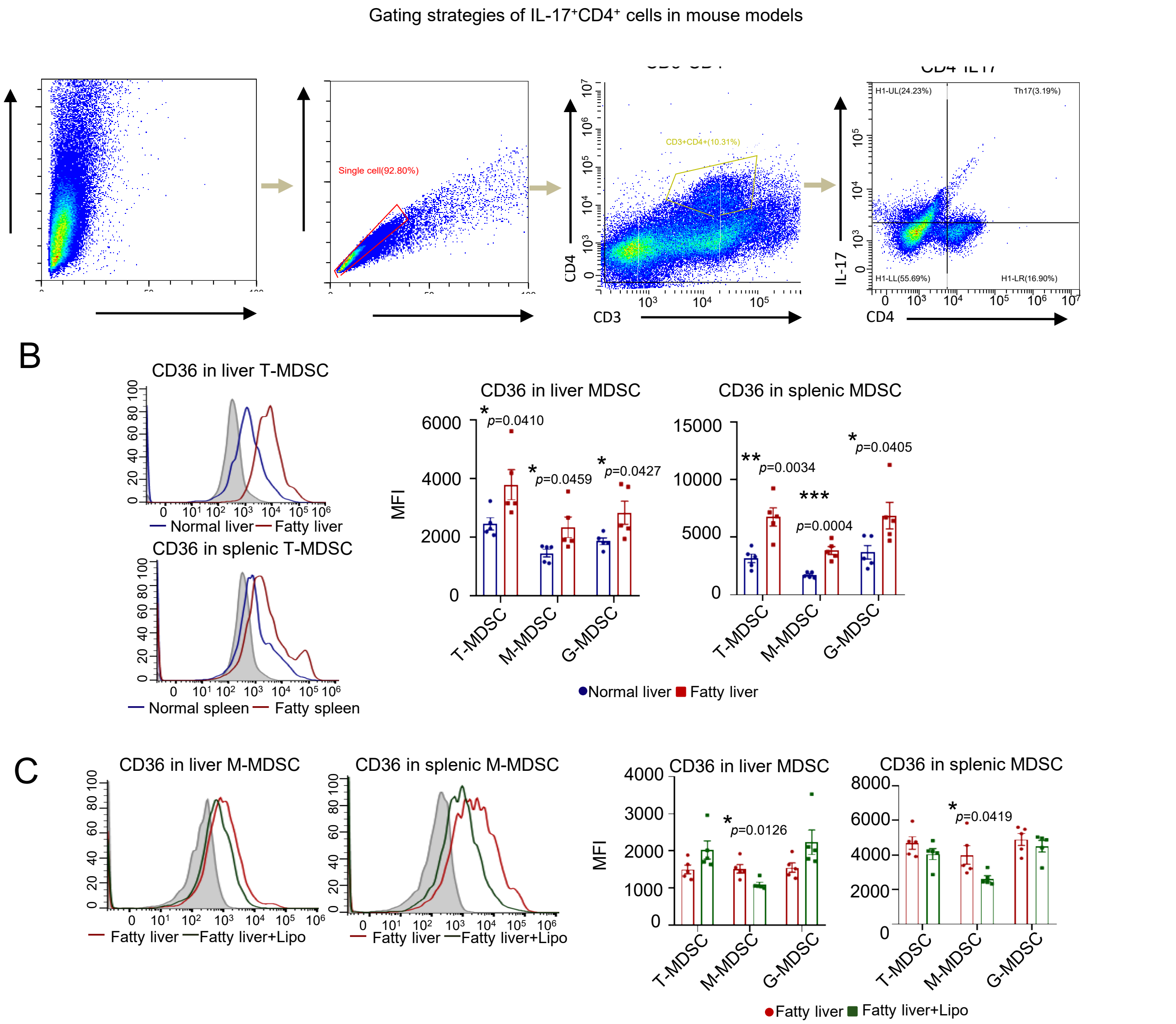

Fig. S7 CD36 in MDSCs was enhanced in fatty liver IRI and inhibited by FATP2 blockade. A The gating strategies of IL-17^{+}CD4^{+} T cells in mouse model; **B** CD36 in MDSCs was increased post fatty liver IRI. **C** FATP2 blockade inhibited CD36 in monocytic MDSCs. Error bars indicate Standard Error of Mean; **p<0.05, **p<0.01, ***p<0.001, ****p<0.0001.* A-C, n=5/group; Lipo: lipofermata; MDSC, myeloid-derived suppressor cell; T-MDSC: total-MDSC; M-MDSC: monocytic-MDSC; G-MDSC: granulocytic-MDSC.

Fig. S8

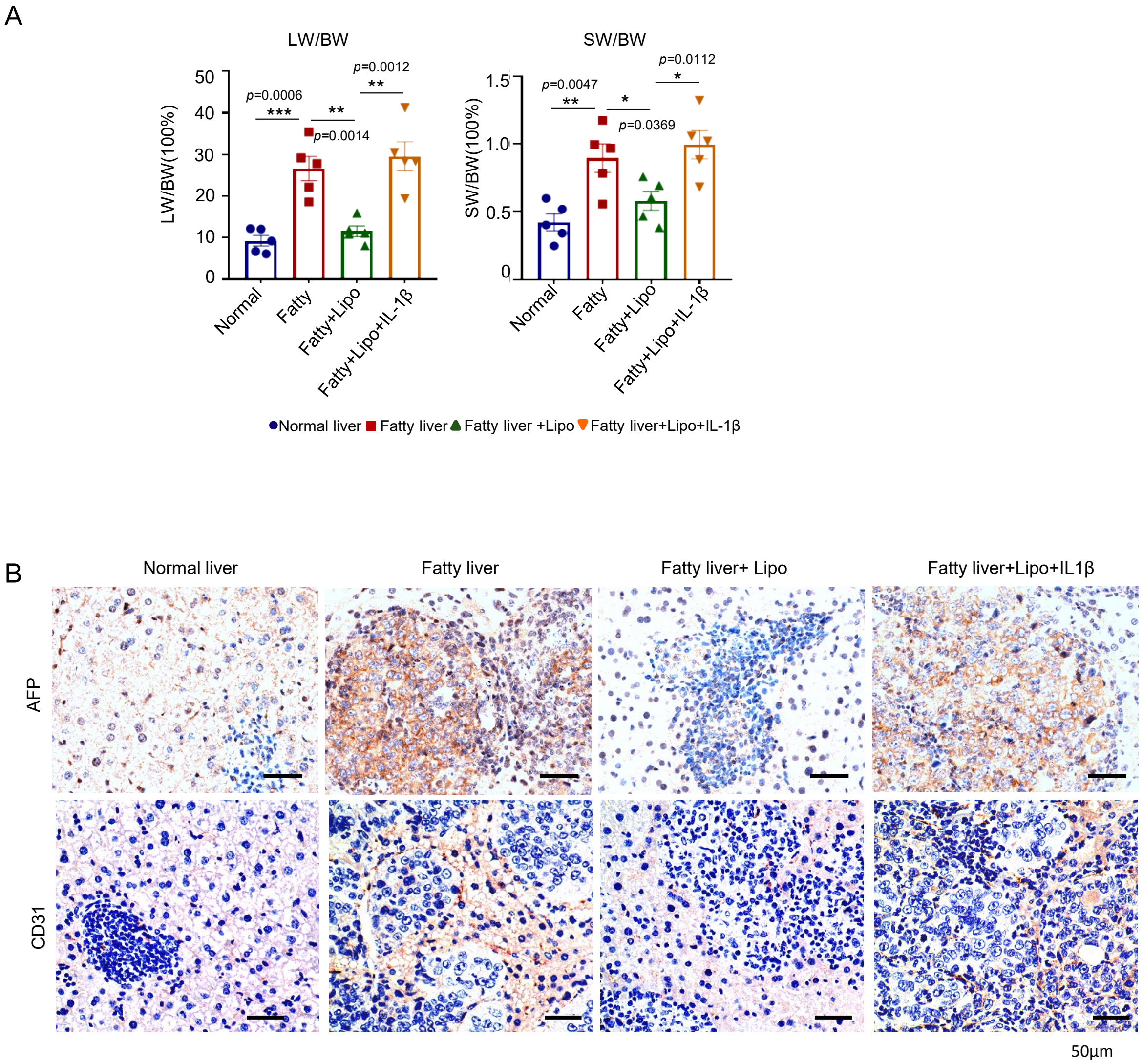

Fig. S8 The increase of tumor burden in fatty liver and reduced by FATP2 inhibition in mouse tumor recurrence model. A The increased liver weight/body weight (LW/BW) and spleen weight/body weight (SW/BW) in fatty liver group was suppressed by FATP2 inhibition and raised by IL-1β. **B** The liver infiltrated alpha fetoprotein (AFP) and CD31 positive cells were increased, decreased and restored in fatty liver, by lipofermata and IL-1β treatment, repectively. Scale bars: 50µm. A-B: n=5/group; Error bars indicate Standard Error of Mean; *$*p<0.05$, **$p<0.01$, ***$p<0.001$, ****$p<0.0001$*. Lipo: lipofermata; 2; LW/BW: liver weight/body weight; SW/BW: spleen weight/body weight; AFP: alpha fetoprotein.

Fig. S9

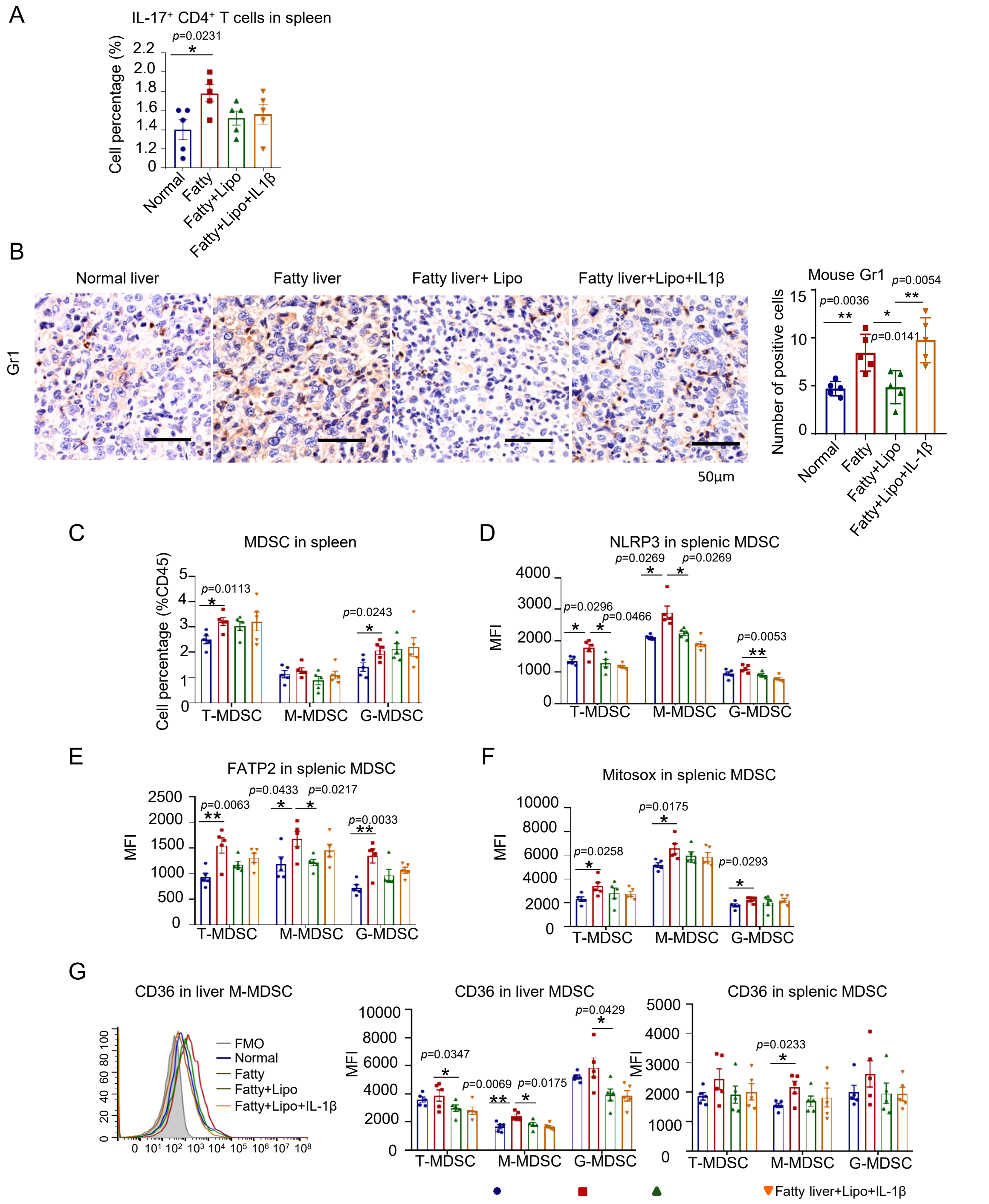

Fig. S9 The alteration of IL-17^{+}CD4^{+}T cells, MDSCs and NLRP3/FATP2/mitosox/CD36 levels in MDSCs of mouse tumor recurrence model. A Splenic CD4^{+}T cells produced more IL-17 in fatty liver tumor recurrence model. **B** The liver infiltrated MDSCs (Gr1^{+}) were increased, decreased and restored in fatty liver, by lipofermata and IL-1β treatment, repectively. Scale bars: 50μm. **C** Splenic T/G-MDSCs were accumulated in fatty liver tumor recurrence. **D** NLRP3 was upregualted in splenic T/M-MDSCs and diminished by lipofermata in fatty liver tumor recurrence. **E** FATP2 was raised in MDSCs while reduced in M-MDSCs by FATP2 inhibition in the spleen of fatty liver tumor recurrence model. **F** The ROS levels were enhanced in splenic MDSCs of fatty liver tumor recurrence model. **G** CD36 was increased in M-MDSCs and decreased in liver MDSCs by FATP2 blockade in fatty liver tumor recurrence model. A-G: n=5/group; Error bars indicate Standard Error of Mean; *$p<0.05$, **$p<0.01$, ***$p<0.001$, ****$p<0.0001$. FMO: fluorescence minus one; AA: arachidonic acid; Lipo: lipofermata; MDSC, myeloid-derived suppressor cell; T-MDSC: total-MDSC; M-MDSC: monocytic-MDSC; G-MDSC: granulocytic-MDSC; NLRP3: nucleotide-binding oligomerization domain-like receptor family pyrin domain containing 3; FATP2: fatty acid transport protein 2. ROS: reactive oxygen species.

Supplementary tables

Table S1 Univariate and multivariate analyses of risk factors for tumor recurrence in HCC patients post living donor liver transplantation.

Table S2 Primers used for the amplification of genes.

	HR	95% CI	*p*
Univariate analysis			
Fatty change (⩾10% vs none)	3.147	(1.046, 9.463)	0.041*
Number of tumors	1.201	(1.103, 1.308)	<0.01**
Largest size of tumour (cm)	1.295	(1.025, 1.636)	0.03*
Vascular permeation	2.920	(1.200, 7.104)	0.018*
Macrovascular invasion	6.366	(1.406, 28.817)	0.016*
New TNM staging	3.212	(1.627, 6.340)	<0.01**
Graft weight to Recipient ESLV (⩾40 vs <40%)	1.073	(0.416, 2.771)	0.884
AFP （⩾20 vs <20 ng/ml）	1.619	(0.671, 3.907)	0.284
Cold ischemic time (⩾120 vs <120 min)	0.993	(0.400, 2.464)	0.988
Recipient warm ischemic time (⩾50 vs <50 min)	1.729	(0.667, 4.483)	0.26
Child-pugh score	0.872	(0.725, 1.050)	0.149
History of treatment for HCC before Tx (treatment vs none)	4.533	(1.506, 13.649)	0.007**
Multivariate analysis			
Fatty change (>10% vs none)	3.901	(1.039, 14.639)	0.044*
Number of tumors	1.153	(1.025, 1.297)	0.018*
Largest size of tumour (cm)	1.279	(0.903, 1.813)	0.166
Vascular permeation	0.896	(0.280, 2.875)	0.854
Macrovascular invasion	4.761	(0.666, 34.040)	0.120
New TNM staging	1.395	(0.476, 4.083)	0.544
Graft weight to Recipient ESLV cater (⩾40 vs <40%)	1.519	(0.436, 5.288)	0.512
AFP （⩾20 vs <20 ng/ml）	2.024	(0.676, 6.060)	0.208
Cold ischemic time (⩾120 vs <120 min)	0.588	(0.190, 1.823)	0.358
Recipient warm ischemic time (⩾50 vs <50 min)	2.506	(0.689, 1.057)	0.104
Child-pugh score	0.853	(0.739, 1.063)	0.146
History of treatment for HCC before Tx (treatment vs none)	2.466	(0.639, 6.519)	0.190

Primers	Forward sequence (5' to 3')	Reverse sequence (5' to 3')
Human NLRP3	CCAAAAGGAAGTGGACTGCG	TCAAACGACTCCCTGGAACG
Human IL-1β	TCAAACGACTCCCTGGAACG	GGAGCGAATGACAGAGGGTT
Rat NLRP3	CCAGGAGTTCTTTGCGGCTA	GCCTTTTTCGAACTTGCCGT
Rat IL-1β	TGACCCATGTGAGCTGAAAG	CAGGGATTTTGTCGTTGCTT
Rat NLRP1	GGACCCCATCACTATGGGAGA	AAAGACTGCTCGACTTGGGG
Rat NLRC4	TCCCAGTAGGGCTGTGTCTT	GCTGGCGGAGCTGAATATCT
Rat AIM2	AAATGCTGTTGTTGACCGGC	CTCCGTCCTGTCTGCAATGT
Rat NLRP6	CTGAGACTGGTGAGCTGTGG	ATTGCCTCACAGAGTGGACG
Mouse NLRP3	TCCCAGACACTCATGTTGCC	GTCCAGTTCAGTGAGGCTCC
Mouse IL-1β	GGCTGGACTGTTTCTAATGCC	TCTTGGCCGAGGACTAAGGA
Mouse ACSL1	ATCTGGTGGAACGAGGCAAG	TCCTTTGGGGTTGCCTGTAG
Mouse ACSL3	AGACCAGGGCTGAGTGGATGAT	CAGACGTGGGACCAAAGAGACTAT
Mouse ACSL5	AAGACGATCATCCTCATGGACC	CCTATATTCTCCGCATCATGCA
Mouse ACSL6	CGGAAGCCAGAGCAACCTTA	ATCCCAGAACCGTTGGTGAC
Mouse FATP1	GCAGCATTGCCAACATGGAC	GTGTCCTCATTGACCTTGACCAGA
Mouse FATP2	CATCGTGGTTGGGGCTACTT	GGTACCGAAGCAGTTCACCA
Mouse FATP3	CTAGAGGAAAAGGGCACCATGGCGGC	GAGCCCCTCCCTCAAGTGGAAGGATT
Mouse FATP4	TGCCCAGTCACCCAGACAAG	CATGCGGAATCCATAGTACACCAG
Mouse FATP5	AGCTATACCAGCATGTCCGC	ACCAGCCGTGACTTTACCAG
Mouse FATP6	ATCGGGTACGTTTGGCAGTT	TCCTTCGGTAGCTCCGTACA
Mouse FASN	GGAGGTGGTGATAGCCGGTAT	TGGGTAATCCATAGAGCCCAG
Mouse CD36	GATGACGTGGCAAAGAACAG	AAAGGAGGCTGCGTCTGTG
Mouse FFAR1	CCATTCTGCTCTTCTTTCTG	GGGTTTATGAAACTAGCCAC
Mouse FFAR2	CCCTGTGCACATCCTCCTGC	GCGTTCCATGCTGATGCCCG
Mouse FFAR3	TGTCCAATACTCTGCATCTGT	AGGTCCGAAATGGTCAGGTT
Mouse HCAR2	GCACAACCAGAAGTATTCCAG	CCAAATCGCCTCTCCAG
Mouse OR51E2	ACGCTGCTGTCCTCAACAAT	ACAGAAAGCCAGTCGCTTGA
Mouse SLC16A1	GTGCCATTTGCTTGCCCCT	TGGTTTTGGATGTCGTGGG
Mouse SLC16A3	ACTGACCTGACAGGCTCCAC	CTTTGGAATGACGCGGTTCC
Mouse SLC16A7	ATGCCATCTCTTATGCCC	TCTGGACGCGTGTGAAGCT
Mouse SLC22A7	CTGGTTGGGTACCTGATACG	CCTGTCTGTCTGAGCACAGT
Mouse SLC21A9	CTTCATGCTCATCCTGAG	GAGGCCTATGAATCGGTT
Mouse SLC26A3	CTGTCTCCTAGAACAGGACTGC	GCCACTGATTAGGGACTCGG
Mouse SLC21A12	TTCGGTCCCAAGTTCTTCG	CACTTCCCTGCGGTAGCAT
Mouse SLC5A8	GCCCCTTGAAACCTATGGCT	CAGTGGAGTCCTTTCCGCAT
Mouse FABP1	GGAAGGACATCAAGGGGGTG	GTCATGGTCTCCAGTTCGCA
Mouse FABP2	AGTTGAGGCCAAGCGATTCT	GCCTGGCATTAGCATGATGG
Mouse FABP3	TGCTGCCTCATGGTTTTCCC	GACCTTGGAGCACCCTTTGG
Mouse FABP4	TGAAATCACCGCAGACGACA	ACACATTCCACCACCAGCTT
Mouse FABP5	GCTGCTTTTGTGCTCTCCCT	GCAGACCGTCTAGCTCTTTCAT
Mouse FABP6	GTGGCAGAGTTCCCCAACTA	TGCTTACGCGCTCATAGGTC
Mouse MSR1	AGGGAAGTGGATAAATCAGTGCT	TGCAGTCAGCATCCTCTTGT
Mouse MARCO	GGGAACATCTGGCTGGACAA	GCTCCCAAGTCAGGAGCATT
Mouse Scarb1	ATAAAGCCTCTGGCCACCTG	ACCTGAAGGAGACGGAGACA
Mouse CD68	TGTTCAGCTCCAAGCCCAAA	GTACCGTCACAACCTCCCTG
Mouse LRP1	GGCGGTGTGACAACGACAAT	CACGTCCAGTGCTCGGGG
Mouse LRP8	TGTCCACACACGGATTGGTT	TTCACAGACAGCAAGTGGCA
Mouse VLDLR	TGACTGTCGGTGTGTGTTGT	CTCAGAGCTGAGTGCCCTTG
Mouse LDLR	GCCAGGAAAGTGACTCGTGA	AAGGGAGAATGGCGACTTGG

Table S3 Antibodies used for the staining.

Name	Citation (PMID)	Supplier	Cat no.	Clone no.
Anti-CD33	8822961	BD Biosciences	555450	WM53
Anti-CD13	2665173	BD Biosciences	557454	WM15
Anti-CD34	26395069	Militenyi Biotech	130-081-001	AC136
Anti-His48	2046327	BD Pharmingen	554907	HIS48
Anti-CD80	9237108	BD Biosciences	555012	3H5
Anti-CD11b/c	-	Invitrogen	MA5-17507	OX-42
Anti-CD11b	6184305	BD Biosciences	557397	M1/70
Anti-CD11b	29276143	Biolegend	101226	M1/70
Anti-Gr1	-	Stem Cell	60028AZ	RB6-8C5
Anti-Gr1	29262351	Biolegend	108406	RB6-8C5
Anti-Ly6G	30955887	Biolegend	127622	1A8
Anti-Ly6C	11754008	BD Biosciences	563011	AL-21
Anti-CD3	29548673	Biolegend	100330	145-2C11
Anti-CD19	29101162	Biolegend	115530	6D5
Anti-CD45	22547694	Biolegend	103138	30-F11
Anti-CD36	11560944	BD Biosciences	562744	CRF D-2712
Anti-NLRP3	31558756	R&D	IC7578A	768319
Anti-SLC27A2	-	Invitrogen	PA5-102343	Polyclonal
Anti-CD4	30610104	Biolegend	116005	RM4-4
Anti-IL-17A	18606690	Biolegend	506909	TC11-18H10.1
Anti-CD44	30194420	Biolegend	103011	IM7
Anti-CD62L	33440157	Invitrogen	63-0621-80	MEL-14
Anti-NLRP3	19364881	Santa Cruz Biotech	sc-66846	Polyclonal
Anti-NLRP3	34651582	Cell signaling	15101S	D4D8T
Anti-ASC	27882934	Sigma-Aldrich	SAB4501315	Polyclonal
Anti-CD36	-	Cell signaling	74002	Polyclonal
Anti-C-caspase-1	33621216	Cell signaling	89332	E2G2I
Anti-CD33	23708142	Leica	NCL-L-CD33	PWS44
Anti-CD11b/c	1672643	BD Pharmingen	550299	OX-42
Anti-CD15	8236092	BD Pharmingen	559045	MMA
Anti-Lox1	32235836	Abcam	ab60178	Polyclonal
Anti-His48	32109414	Abcam	ab33760	HIS48
Anti-VDAC	35505004	Cell signaling	4661p	D73D12
Anti-SDHB	33431792	Abcam	ab14714	21A11AE7
Anti-PHB1	32778760	Cell signaling	2426	Polyclonal
Anti-SOD1	32402267	Cell signaling	4266	71G8
Anti-DRP1	18838687	Cell signaling	14647	4E11B11
Anti-NDUFB8	34697471	Invitrogen	459210	20E9DH10C12
Anti-UQCRC2	-	Invitrogen	PA5-53939	Polyclonal
Anti-MT-CO1	24360282	Cell signaling	62101	Polyclonal
Anti-ATP5A1	35900274	Cell signaling	18023	Polyclonal
Anti-AFP	34958137	Abcam	ab46799	Polyclonal
Anti-CD31	25079331	Invitrogen	PA5-16301	Polyclonal

Supplementary figure legends

Fig. S1 The tumor recurrent rate was higher in HCC patients underwent liver transplantation using steatotic donor accompanied with the increase of MDSCs and NLRP3/FATP2 levels. A The tumor recurrence free survival was relatively poor in HCC patients after liver transplantation using steatotic donor (n=88). **B** The gating strategies of human MDSCs ($CD33^+CD13^+CD34^+$) by flow cytometry (n=45). **C** The number of MDSCs ($CD33^+$) were significantly accumulated in steatotic grafts by immunohistochemistry (n=23). **D** More MDSCs were infiltrated in fatty grafts by co-staining of CD15 & LOX1 (n=23). Scale bars: 20μm. **E** The increased numbers of NLRP3 and FATP2 positive cells in fatty grafts by immunohistochemistry (n=23). **F** More $CD33^+NLRP3^+$ and $CD33^+FATP2^+$ cells were infiltrated in fatty grafts post liver transplantation by co-staining (n=23). Error bars indicate Standard Error of Mean; *$p<0.05$, **$p<0.01$, ***$p<0.001$, ****$p<0.0001$. MDSC, myeloid-derived suppressor cell; NLRP3: nucleotide-binding oligomerization domain-like receptor family pyrin domain containing 3; FATP2: fatty acid transport protein 2.

Fig. S2 The levels of NLRP3 and FATP2 were higher with accumulation of MDSCs in fatty liver grafts of rat model. A The gating strategies of rat MDSCs ($CD11b/c^+CD80^+His48^+$) by flow cytometry. **B** The number of $CD11b/c^+$ (MDSC marker) cells were obviously increased in rat fatty liver grafts by immunohistochemistry. **C** Accumulated MDSCs in fatty grafts by co-staining of CD11b/c & His48. Scale bars: 20μm. **D** NLRP3 was upreglated in fatty grafts through the inflammsome (NLRP1, NLRP3, NLRC4, AIM2, NLRP6) mRNA screen of rat liver transplantation model. **E** NLRP3 and FATP2 positive cells were more in fatty liver grafts by immunohistochemistry. **F** The immunofluorescent co-staining showed increased

CD11b/c$^+$NLRP3$^+$ and CD11b/c$^+$FATP2$^+$ cells in fatty grafts post liver transplantation. A-F: n=4/group; Error bars indicate Standard Error of Mean; $^*p<0.05$, $^{**}p<0.01$, $^{***}p<0.001$, $^{****}p<0.0001$. MDSC, myeloid-derived suppressor cell; NLRP: nucleotide-binding oligomerization domain-like receptor family pyrin domain containing; FATP2: fatty acid transport protein 2; NLRC4: NLR Family CARD Domain Containing 4; AIM2: Absent In Melanoma 2.

Fig. S3 The severer inflammatory injury and poorer liver function with enhanced NLRP3 and IL-1β expressions in mice post fatty liver ischemia/reperfusion injury (IRI). A The inflammatory markers, included MCP-1, IL-6, CXCL1, IFN-γ, IL-1β and TLR4, were significantly upregulated post fatty liver IRI. **B** The liver function was poorer with increased alanine transaminase (ALT) and aspartate aminotransferase (AST) in fatty liver after IRI. **C** The mRNA levels of NLRP3 and IL-1β were increased in fatty liver post IRI in mice**.** A-C: n=5/group; Error bars indicate Standard Error of Mean; $^*p<0.05$, $^{**}p<0.01$. NLRP3: nucleotide-binding oligomerization domain-like receptor family pyrin domain containing 3; IRI, ischemia/reperfusion injury; MCP-1: Monocyte Chemoattractant Protein 1; IL-6: Interleukin-6; CXCL1: C-X-C Motif chemokine ligand 1; IFN-γ: Interferon-γ; TNF-α: tumor necrosis factor α; TLR4: Toll Like Receptor 4; ALT: alanine transaminase; AST, aspartate aminotransferase.

Fig. S4 No obvious change of Dil-LDL and free fatty acid in MDSCs of mice post fatty liver IRI. A The gating strategies of Total MDSC (T-MDSC, CD11b$^+$Gr1$^+$), Granulocytic MDSC (G-MDSC, CD11b$^+$ Ly6G$^+$Ly6C^{low}) and Monocytic MDSC (M-MDSC, CD11b$^+$ Ly6G$^-$Ly6C^{high}) in mice. **B** Dil-LDL in MDSCs was not significantly changed in mice after fatty liver IRI. **C** Free fatty acids were not altered obviously post fatty liver IRI. A-C: n=5/group; Error bars indicate Standard Error of Mean; $^*p<0.05$, $^{**}p<0.01$. IRI, ischemia/reperfusion injury; MDSC, myeloid-

derived suppressor cell; LDL: low density lipoprotein; T-MDSC: total-MDSC; M-MDSC: monocytic-MDSC; G-MDSC: granulocytic-MDSC.

Fig. S5 NLRP3 in primary MDSCs was not changed by C18:0 stimulation. **A** FL C16 in primary MDSCs was not obviously altered by C18:0 or SSO (CD36 inhibitor) treatment. **B** C18:0 or SSO could not activate or inhibit NLRP3 in MDSCs, respectively. **C** CD36 in MDSCs was not significantly changed by C18:0 or SSO treatment. Error bars indicate Standard Error of Mean; NLRP3: nucleotide-binding oligomerization domain-like receptor family pyrin domain containing 3; MDSC, myeloid-derived suppressor cell; SSO: Sulfosuccinimidyl oleate sodium; T-MDSC: total-MDSC; M-MDSC: monocytic-MDSC; G-MDSC: granulocytic-MDSC.

Fig. S6 The major proteins of mitochondria and ATPs were inhibited/enhanced by arachidonic acid or FATP2 blockade while mitochondrial counts were not changed obviously. **A** CD36 in primary MDSCs was upregulated and decreased by arachidonic acid (AA) and lipofermata (Lipo, FATP2 inhibitor), respectively. **B** The counts of mitochondria were not obviously changed by AA or lipofermata treatment. **C** The major mitochondrial protein levels, including NDUFB8 (complex I), SDHB (complex II), UQCRC2 (complex III), MT-CO1 (complex IV), ATP5A1 (complex V), VDAC, PHB1 and SOD1 in MDSCs were obviously decreased by AA but increased through the inhibition of FATP2. DRP1, the damage marker of mitochondria, was increased by AA but reduced by FATP2 inhibition. **D** The adenosine triphosphates (ATPs) produced by MDSCs were decreased by AA but enhanced by FATP2 blocking. **E** The gating strategies of IL-17 levels in $CD4^+$ T cells *in vitro*. **F** The mitosox was only increased in splenic total MDSCs. **G** The counts of mitochondria were not significantly altered post fatty liver IRI. F-G: n=5/group; Error bars indicate Standard Error of Mean; $^*p<0.05$, $^{**}p<0.01$, $^{***}p<0.001$,

$^{****}p<0.0001$. NC: negative control, the primary MDSCs without stimulation; FMO: fluorescence minus one; AA: arachidonic acid; Lipo: lipofermata; MDSC, myeloid-derived suppressor cell; T-MDSC: total-MDSC; M-MDSC: monocytic-MDSC; G-MDSC: granulocytic-MDSC; NDUFB8: NADH dehydrogenase (ubiquinone) 1 beta subcomplex subunit 8; SDHB: succinate dehydrogenase B; UQCRC2: ubiquinol-cytochrome C reductase core protein 2; MT-CO1: mitochondrially encoded cytochrome C oxidase I; ATP5A1: ATP synthase alpha-subunit; DRP1: dynamin-related protein 1; VDAC: voltage-dependent anion channel; PHB1: prohibitin 1; SOD1: superoxide dismutase 1; ATPs: adenosine triphosphates.

Fig. S7 CD36 in MDSCs was enhanced in fatty liver IRI and inhibited by FATP2 blockade. **A** The gating strategies of IL-17^{+}CD4^{+} T cells in mouse model; **B** CD36 in MDSCs was increased post fatty liver IRI. **C** FATP2 blockade inhibited CD36 in monocytic MDSCs. Error bars indicate Standard Error of Mean; $^{*}p<0.05$, $^{**}p<0.01$, $^{***}p<0.001$, $^{****}p<0.0001$. A-C, n=5/group; Lipo: lipofermata; MDSC, myeloid-derived suppressor cell; T-MDSC: total-MDSC; M-MDSC: monocytic-MDSC; G-MDSC: granulocytic-MDSC.

Fig. S8 The increase of tumor burden in fatty liver and reduced by FATP2 inhibition in mouse tumor recurrence model. **A** The increased liver weight/body weight (LW/BW) and spleen weight/body weight (SW/BW) in fatty liver group was suppressed by FATP2 inhibition and raised by IL-1β. **B** The liver infiltrated alpha fetoprotein (AFP) and CD31 positive cells were increased, decreased and restored in fatty liver, by lipofermata and IL-1β treatment, repectively. Scale bars: 50μm. A-B: n=5/group; Error bars indicate Standard Error of Mean; $^{*}p<0.05$, $^{**}p<0.01$, $^{***}p<0.001$, $^{****}p<0.0001$. FATP2: fatty acid transport protein 2; LW/BW: liver weight/body weight; SW/BW: spleen weight/body weight; AFP: alpha fetoprotein.

Fig. S9 The alteration of IL-17$^+$CD4$^+$T cells, MDSCs and NLRP3/FATP2/mitosox/CD36 levels in MDSCs of mouse tumor recurrence model. **A** Splenic CD4$^+$ T cells produced more IL-17 in fatty liver tumor recurrence model. **B** The liver infiltrated MDSCs (Gr1$^+$) were increased, decreased and restored in fatty liver, by lipofermata and IL-1β treatment, repectively. Scale bars: 50μm. **C** Splenic T/G-MDSCs were accumulated in fatty liver tumor recurrence. **D** NLRP3 was upregualted in splenic T/M-MDSCs and diminished by lipofermata in fatty liver tumor recurrence. **E** FATP2 was raised in MDSCs while reduced in M-MDSCs by FATP2 inhibition in the spleen of fatty liver tumor recurrence model. **F** The ROS levels were enhanced in splenic MDSCs of fatty liver tumor recurrence model. **G** CD36 was increased in M-MDSCs and decreased in liver MDSCs by FATP2 blockade in fatty liver tumor recurrence model. A-G: n=5/group; Error bars indicate Standard Error of Mean; *$p<0.05$, **$p<0.01$, ***$p<0.001$, ****$p<0.0001$. FMO: fluorescence minus one; AA: arachidonic acid; Lipo: lipofermata; MDSC, myeloid-derived suppressor cell; T-MDSC: total-MDSC; M-MDSC: monocytic-MDSC; G-MDSC: granulocytic-MDSC; NLRP3: nucleotide-binding oligomerization domain-like receptor family pyrin domain containing 3; FATP2: fatty acid transport protein 2; ROS: reactive oxygen species.

Supplementary materials and methods

Quantitative Real-Time Polymerase Chain Reaction (qRT-PCR)

Total RNA was extracted from human liver specimens, rat/mouse liver tissues and MDSCs isolated from mouse liver/spleen using Trizol Reagent (Invitrogen, CA, USA). Complementary DNA was synthesized from 1μg or 500ng total RNA using High Capacity cDNA Reverse Transcription Kits (Applied Biosystems, CA, USA). qRT-PCR was performed using Viia7 Fast Real-time PCR system (Life Technologies, CA, USA) with primers listed in **Table S2** and Fast Start SYBR Green Master Mix (Promega, WI, USA).

Flow Cytometry Analysis

Flow cytometry was carried out with a modified version of the previous protocol(1). Human MDSCs were detected by CD33, CD13 (BD Pharmingen, CA, USA) and CD34 (Militenyi Biotech, Bergisch Gladbach, Germany). Rat MDSC population was detected by incubation with His48, CD80 (BD Pharmingen), CD11b/c (Invitrogen) antibodies. Mouse total MDSCs was labeled by Gr1 (Biolegend, CA, USA/Stem cell, Stemcell Technologies, BC, Canada), CD11b (BD Pharmingen/ Biolegend). The monocytic/granulocytic MDSCs of mouse was checked by CD11b (BD Pharmingen/Biolegend), Ly6G (Biolegend) and Ly6C (BD Horizon). Moreover, mouse CD45, CD3 and CD19 (Biolegend) were stained to gate the cells and avoid the lymphocytes. NLRP3 (R&D, MN, USA), FATP2 (Invitrogen), CD36 (BD Pharmingen), IL17 (Biolegend) antibodies were used to investigate their levels. BODIPY FL C16, 493/503, low density lipoprotein from human plasma-DiI complex (Dil LDL), MitoTracker green FM, MitoSox red mitochondrial superoxide indicator (Invitrogen) and free fatty acid (Abcam, Cambridge, MA, USA) was stained to investigate the lipid level and mitochondrial alteration. The purity of primary MDSCs and naïve

$CD4^+$ T cells were determined by $Gr1^+CD11b^+$ (Biolegend) and $CD4^+CD44^+CD62L$ (Biolegend/Invitrogen), respectively. Before detecting the IL-17 levels in $CD4^+$ T cells, the single cell suspension from liver and spleen of mouse model was stimulated with cell activation cocktail (Brefeldin A, Biolegend) for at least 4 hours at 37 °C. The cells were implemented to permeabilization for 30 mins. After that, IL-17 was stained to explore the levels by flow cytometry. Labeled cells were analyzed by BD FACS Calibur (BD Biosciences, CA, USA), Cytoflex S (Beckman Coulter, CA, USA), FlowJo (Treestar, San Carlos, CA, USA).

Immunostaining

The detailed protocols of immunohistochemistry and immunofluorescent staining was performed using the previous methods with modification(1-3). Antibodies against human CD33 (Leica, Milton Keynes, UK), human CD15 (BD Pharmingen), LOX1 (Abcam), rat CD11b/c (BD Pharmingen), rat His 48 (Abcam), human/rat/mouse NLRP3 (Santa Cruz, CA, USA) and FATP2 (Invitrogen) were used. Mitochondria was detected by MitoTracker red CMXRos/green FM, MitoSox red mitochondrial superoxide indicator (Invitrogen) and Dihydrorhodamine 123 (Sigma-Aldrich, Darmstadt, Germany). The Hamamatsu imaging system (Hamamatsu Photonics, Shizuoka, Japan) and Carl Zeiss LSM 780 imaging System (Carl Zeiss, Jena, Germany) was used to acquire the images and count the positive cells of five randomly chosen views in each slide (×400) for statisctics.

RNA sequencing

Total RNA was extracted from rat liver graft at 6 hours after transplantation using normal or fatty donor (n=3). RNA high throughout sequencing was performed using Illumina PE150. RNA-seq data have been submmited and are available through the NCBI's Gene Expression Omnibus (GEO

GSE204919). The results were analyzed by Kyoto Encyclopedia of Genes and Genomes and Gene Set Enrichment Analysis.

Gas chromatography-mass spectrometry analysis of fatty acids

The tissues of rat normal/fatty liver and grafts at 6 hours after liver transplantation using normal/fatty donor were prepared for gas chromatography-mass spectrometry analysis (n=4). The whole procedure was finished by Center for Proteomics and Metabolomics core, Core PanorOmic Sciences, LKS faculty of Medicine, The University of Hong Kong. The detailed protocols could be acquired from the core. Total 36 types of fatty acids (medium to long chain) were detected including C4:0, C8:0, C10:0, C11:0, C12:0, C13:0, C14:0, C14:1, C15:0, C15:1, C16:0, C16:1, C17:0, C17:1, C18:0, C18:1, trans, C18:1, cis, C18:2, trans, C18:2, cis, C18:3 n6, C18:3 n3, C20:0, C20:1 n9, C20:2, C21:0, C20:3 n6, C20:4 n6, C20:3 n3, C20:5, C22:0, C22:1, C22:2, C23:0, C24:0, C22:6, C24:1, C6:0, C26:0. The changed fatty acids were analyzed.

Western blot

Western blot was carried out according to the protocol described previously(3). Anti-mouse FATP2 (Invitrogen), ASC (Sigma-Aldrich), NLRP3, cleaved-caspase-1, CD36, MT-CO1, ATP5A1, DRP1, VDAC, PHB1, SOD1 (Cell Signaling Technology, MA, USA), NDUFB8, UQCRC2 (Invitrogen), SDHB (Abcam) antibodies were used.

ATP quantification assay

The evaluation of total ATPs in MDSCs was performed according to the manufacturer's instructions of ATP colorimetric assay (Abcam). Briefly, the isolated MDSCs with arachidonic acid/lipofermata stimulation was homogenized in 100 µl ATP assay buffer and the homogenate was centrifuged (13,000g, 5min, 4°C) to harvest supernatant. Add cold 4M perchloric acid to a

final concentration 1M in the supernatant, mix well and centrifuge (13,000g, 5min, 4℃). One-third volume of 2M KOH was added to the supernatant and the mixture pH was adjusted to 6.5-8.0. The mixture was centrifuged (13,000g, 15min, 4℃) again to obtain supernatant. Add 50μL reaction mixture or background reaction mixture with equal volume of the last supernatant to each well and incubate at room temperature for 30min. The result was read from the absorbance at OD 570nm.

Supplementary references

1. Ling C-C, Ng KT, Shao Y, Geng W, Xiao J-W, Liu H*, et al.* Post-transplant endothelial progenitor cell mobilization via CXCL10/CXCR3 signaling promotes liver tumor growth. J Hepatol **2014**;60(1):103-9.
2. Yeung OW, Lo C-M, Ling C-C, Qi X, Geng W, Li C-X*, et al.* Alternatively activated (M2) macrophages promote tumour growth and invasiveness in hepatocellular carcinoma. J Hepatol **2015**;62(3):607-16.
3. Liu H, Lo CM, Yeung OWH, Li CX, Liu XB, Qi X*, et al.* NLRP3 inflammasome induced liver graft injury through activation of telomere-independent RAP1/KC axis. The Journal of pathology **2017**;242(3):284-96.
